# Synthesis of 4” manipulated Lewis X trisaccharide analogues

**DOI:** 10.3762/bjoc.8.126

**Published:** 2012-07-23

**Authors:** Christopher J Moore, France-Isabelle Auzanneau

**Affiliations:** 1 Department of Chemistry, University of Guelph, 50 Stone Rd. East, Guelph, Ontario, N1G 2W1, Canada

**Keywords:** chlorodeoxygalactose, fluorodeoxygalactose, Lewis X analogues, oligosaccharide synthesis

## Abstract

Three analogues of the Le^x^ trisaccharide antigen (β-D-Gal*p*(1→4)[α-L-Fuc*p*(1→3)]-D-GlcNAc*p*) in which the galactosyl residue is modified at O-4 as a methyloxy, deoxychloro or deoxyfluoro, were synthesized. We first report the preparation of the modified 4-OMe, 4-Cl and 4-F trichloroacetimidate galactosyl donors and then report their use in the glycosylation of an *N*-acetylglucosamine glycosyl acceptor. Thus, we observed that the reactivity of these donors towards the BF_3_·OEt_2_-promoted glycosylation at O-4 of the *N*-acetylglucosamine glycosyl acceptors followed the ranking 4-F > 4-OAc ≈ 4-OMe > 4-Cl. The resulting disaccharides were deprotected at O-3 of the glucosamine residue and fucosylated, giving access to the desired protected Le^x^ analogues. One-step global deprotection (Na/NH_3_) of the protected 4”-methoxy analogue, and two-step deprotections (removal of a *p*-methoxybenzyl with DDQ, then Zemplén deacylation) of the 4”-deoxychloro and 4”-deoxyfluoro protected Le^x^ analogues gave the desired compounds in good yields.

## Introduction

A glycolipid displaying the dimeric Le^x^ hexasaccharide (dimLe^x^) has been identified as a cancer associated carbohydrate antigen, particularly prevalent in colonic and liver adenocarcinomas. In addition, an association between the fucosylation of internal GlcNAc residues in polylactosamine chains, and metastasis and tumor progression in colorectal cancers has been suggested [1–6]. Unfortunately, dimLe^x^ displays the Le^x^ trisaccharide (β-D-Gal*p*(1→4)[α-L-Fuc*p*(1→3)]-D-GlcNAc*p*), at the nonreducing end, and this antigenic determinant is also present at the surface of many normal cells and tissues, which include kidney tubules, gastrointestinal epithelial cells, and cells of the spleen and brain [7–11]. Indeed, anti-Le^x^ monoclonal antibodies (e.g., FH3, SH1) have been shown to recognize this Le^x^ antigenic determinant as it exists in the hexasaccharide [1–6]. Therefore, as our group embarks on the development of a therapeutic anticancer vaccine utilizing the Tumor Associated Carbohydrate Antigen (TACA) dimLe^x^ as a target, an important factor to consider is an expected autoimmune response to the native Le^x^ antigen. Fortunately an internal epitope displayed by dimLe^x^ was discovered when monoclonal antibodies (mAbs) FH4 and SH2, raised against dimLe^x^, were isolated. Indeed, these mAbs were shown to bind to the dimLe^x^ and trimLe^x^ antigens but only weakly recognise Le^x^ trisaccharide antigen [1–6]. With this in mind, we focus our research on the discovery of analogues of dimLe^x^ that can be used as safe vaccine candidates. Ideally, these analogues should display the internal epitopes that are recognized by anti-dimLe^x^ SH2-like antibodies while being free of those that are recognized by anti-Le^x^ SH1-like antibodies.

**Figure 1:**
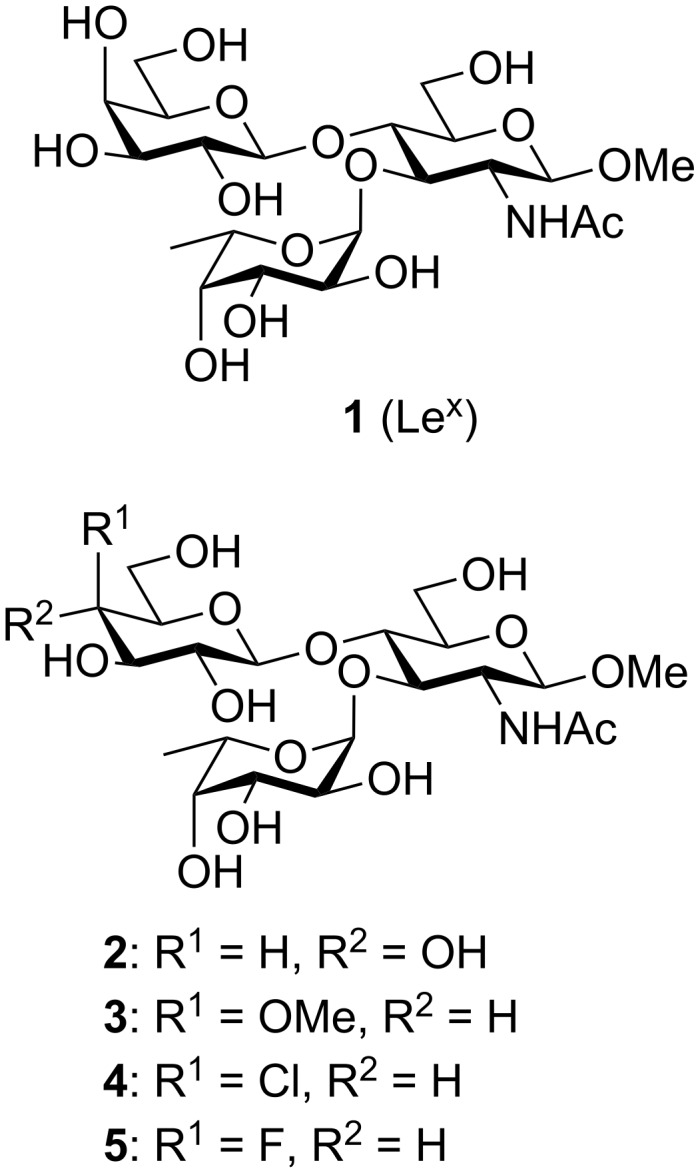
Structure of Le^x^ and analogues **2**–**5**.

In order to abolish cross-reactivity with the Le^x^ antigen, we have prepared [12–14] several analogues in an attempt to identify a suitable replacement for the nonreducing end Le^x^ trisaccharide. In turn, we have compared the conformational behaviour of these analogues to that of the natural Le^x^-OMe **1** (Figure 1) through a mixture of stochastic searches and NMR analyses [15]. The results pointed toward the preferential adoption, by all of analogues, of the stacked conformation that has been assigned for the Le^x^ trisaccharide [16–21]. The relative affinity of the anti-Le^x^ monoclonal antibody SH1 [6] for the native Le^x^ antigen **1** and our Le^x^ analogues [12–14] was examined by competitive ELISA experiments using a Le^x^-BSA glycoconjugate as the immobilized ligand [15,22–24].

It was discovered that the Le^x^ analogue **2**, with a glucose unit in place of the galactose residue (Figure 1), did not bind to the SH1 mAb, even at high concentrations [15]. This discovery suggests that, to conserve cross-reactivity with the natural Le^x^ antigen, the nonreducing end galactose is essential, and that modifying this residue, particularly at O-4, may lead to the complete loss of this cross-reactivity [15]. Currently, it is not known what the reason for this observed loss of binding is, since the binding affinities between proteins and carbohydrates are the result of a combination of factors [25–29]. One of the main interactions is the formation of hydrogen bonds, either direct or water-mediated, between the amino acid residues of the protein and the key binding hydroxy groups of the ligand, which are arranged in clusters presented by different monosaccharide units. Other factors include favourable interactions of the nonpolar amino acid residues with the hydrophobic patches exhibited by the ligand, as well as high-energy water molecules being favourably displaced from the combining site. Binding affinity is therefore a result of combined enthalpic, entropic and solvation effects, frequently leading to a balance between favourable enthalpic and unfavourable entropic contributions [25–29]. Thus, only additional competitive ELISA studies with Le^x^-OMe analogue inhibitors containing strategic manipulations at the 4” site will provide further insight into specific binding interactions [15]. The synthesis of a 4”-deoxy Le^x^ trisaccharide analogue was reported recently by Dong et al. [30]. Here, we report the synthesis of 4”-methyloxy, 4”-deoxychloro and 4”-deoxyfluoro Le^x^-OMe analogue inhibitors **3**–**5**.

## Results and Discussion

**Figure 2:**
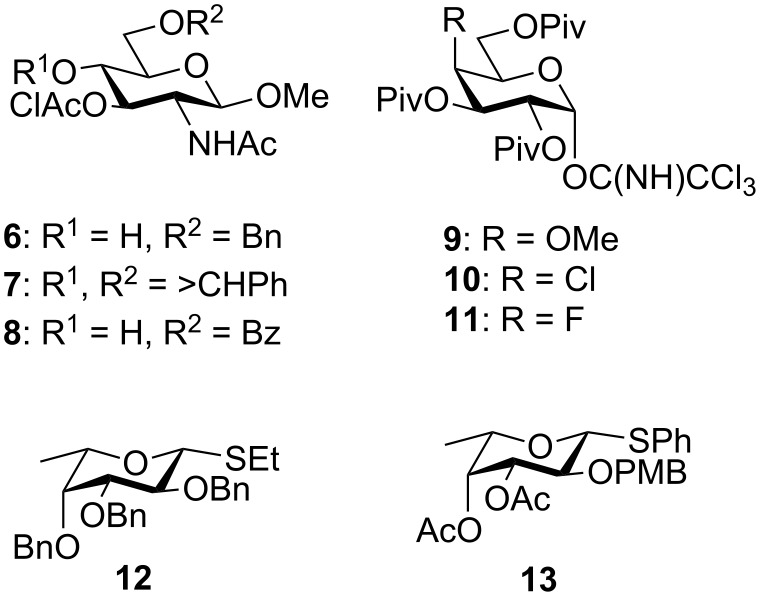
Monosaccharide building blocks **6**–**13**.

There are numerous reports in the literature of the chemical preparation of Le^x^ analogues [30–55], including one recent report of the synthesis of a Le^x^ pentasaccharide 4-deoxy at the nonreducing end galactosyl residue [30]. These syntheses follow one of three strategies: (1) the galactosylation then fucosylation of a glucosamine acceptor [30–47]; (2) the fucosylation then galactosylation of a glucosamine acceptor [48–53]; or (3) the fucosylation at O-3 of a lactosamine derivative prepared from lactose [54–55]. Our synthetic approach to prepare analogues **3**–**5** followed the first strategy [30–47] and involved the use of *N*-acetylglucosamine glycosyl acceptors **6** [14] and **8**, galactosyl donors **9**–**11**, and known fucosyl donors **12** [56] and **13** [12] (Figure 2).

### Synthesis of monosaccharide building blocks **8**–**11**

Glucosamine acceptor **8** was prepared in two steps from the known [14] benzylidene **7**: the benzylidene acetal was first hydrolyzed, and then the resulting diol (79%) was selectively benzoylated at O-6 (BzCl-collidine) giving acceptor **8** in 62% yield.

**Scheme 1:**
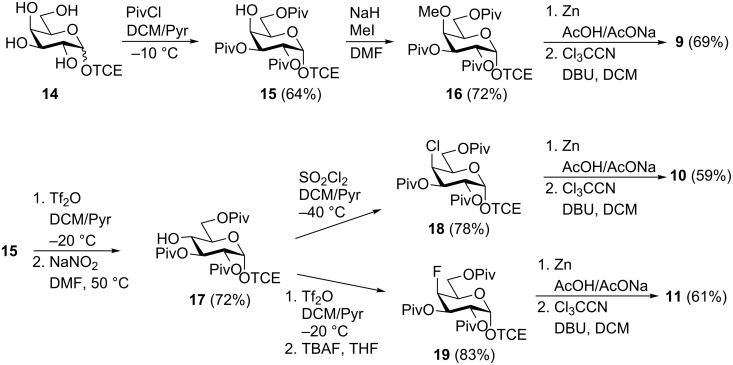
Synthesis of trichloroacetimidate donors **9**–**11**.

The syntheses of trichloroacetimidate donors **9**–**11** are described on Scheme 1; they were all prepared from the known trichloroethyl galactoside **14** [57]. Galactoside **14** was first prepared in three steps from galactose: (1) peracetylation (Ac_2_O-pyridine); (2) BF_3_·OEt_2_ activation of the anomeric acetate and glycosidation with trichloroethanol; and (3) Zemplén deacetylation. This sequence of reactions gave the desired galactoside **14** in 78% yield and as a 9:1 α/β mixture, as assessed by ^1^H NMR. It is important to point out that the second step in this sequence of reactions used conditions very similar to those used by Risbood et al. to prepare peracetylated trichloroethyl galactopyranoside from peracetylated galactose. Indeed, in agreement with their work [57], the ratio of α-anomer isolated here suggests a late anomerization of the β-glycoside during our extended reaction time (20 h) at reflux. The 4-methyloxy trichloroacetimidate donor **9** was then prepared in four steps from the anomeric mixture of galactoside **14**. Tetraol **14** was stirred in a mixture of pyridine and dichloromethane at −10 °C and treated with 3.1 equivalents of pivaloyl chloride. Under these conditions the α-tripivaloate **15**, selectively acylated at O-2, O-3 and O-6, was obtained pure and free of β-anomer (64%). The free hydroxy group in alcohol **15** was then deprotonated with sodium hydride and allowed to react with methyl iodide, yielding the 4-OMe galactoside **16** in very good yield. In turn, trichloroethyl galactoside **16** was converted to the trichloroacetimidate donor **9** in two steps: the anomeric trichloroethyl group was removed (Zn/AcOH), and then the resulting hemiacetal was treated with trichloroacetonitrile in the presence of DBU giving the desired α-trichloroacetimidate in good yield.

A Lattrell-Dax nitrite mediated inversion [58–60] of the 4-OH in galactoside **15** provided the glucoside **17**, which was used as the common precursor to analogues **18** and **19**. Treatment with sulfuryl chloride [61] gave the 4-chloro galactoside **18** in good yield, whereas triflation at O-4 followed by S_N_2 displacement of the triflate by using tetrabutylammonium fluoride [62–63] gave the 4-fluoro galactoside analogue **19** in excellent yields. As described above for the preparation of donor **9** from glycoside **16**, trichloroethyl galactosides **18** and **19** were, in turn, converted in two steps (Zn/AcOH then Cl_3_CCN/DBU) to the trichloroacetimidate donors **10** and **11**, respectively, which were obtained in acceptable yields over the two steps.

### Galactosylation at O-4 of *N*-acetylglucosamine acceptors

It has been well established that the hydroxy group at C-4 of *N*-acetylglucosamine is a poor nucleophile, with reduced reactivity toward glycosylation [64–66]. However, we have reported the successful O-4 glycosylation of an *N*-acetylglucosamine monosaccharide acceptor using peracetylated gluco- and galactopyranose α-trichloroacetimidate donors under activation with 2 equivalents of BF_3_·OEt_2_ at room temperature or 40 °C [14,67]. We applied similar conditions to prepare disaccharides **20**–**22** (Table 1). Glycosylation of methyl glycoside **6** with the 4-methoxy donor **9** gave the best results when the reaction was carried out at 40 °C and left to proceed for 2 hours. Under these conditions, the desired disaccharide **20** was isolated in acceptable yields (Table 1, entries 1 and 2). Our attempts to reduce the number of equivalents of donor **9** used in the reaction always resulted in a lower yield of the desired disaccharide. Glycosylation of acceptor **8** with the 4-chloro galactosyl donor **10** appeared to be slower (Table 1, entries 3–5) than that of acceptor **6** with donor **9**. The best results were obtained when the reaction was left to proceed for 3 rather than 2 hours (Table 1, entry 4), and the desired disaccharide **21** was then obtained in acceptable yield. Increasing the temperature to 60 °C did not increase the yield, presumably due to the degradation of the glycosyl donor at this higher temperature (Table 1, entry 5). Of the three glycosylations considered here, the coupling of acceptor **8** with the 4-fluoro donor **11** gave the highest yields (Table 1, entries 6 and 7). Indeed the desired disaccharide **22** was obtained in very good yields when the reaction was allowed to proceed for 2 hours at 40 °C. Once again, increasing the temperature to 60 °C offered no advantage and in fact led to a lower yield of the desired product.

**Table 1:** Glycosylation at O-4 of glucosamine with donors **9**–**11**.^a^

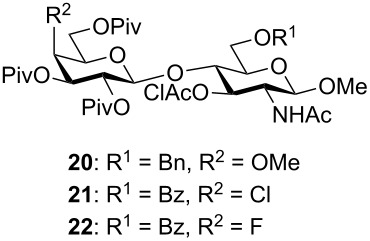					

Entry	Acc.	Don.	*T* (°C)	Time (h)	Product (%)

1	**6**	**9**	rt^b^	2	**20** (65)
2	**6**	**9**	40^b^	2	**20** (70)
3	**8**	**10**	40^b^	2	**21** (55)
4	**8**	**10**	40^b^	3	**21** (63)
5	**8**	**10**	60^c^	2	**21** (62)
6	**8**	**11**	40^b^	2	**22** (77)
7	**8**	**11**	60^c^	2	**22** (73)

^a^Reagents and conditions: BF_3_·Et_2_O (2 equiv), donor **9**–**11** (5 equiv); ^b^solvent: CH_2_Cl_2_; ^c^solvent: 1,2-dichloroethane.

From these three reactions, it is clear that the substituent at O-4 of a galactosyl donor impacts the outcome of glycosylation at O-4 of *N*-acetylglucosamine acceptors. Indeed, we have previously observed that galactosylations of such acceptors [68–69] usually result in lower yields (~70%) than those for analogous glucosylations, which usually provided around 90% of the desired disaccharides [14,67]. The results described here indicate that 4-OAc galactosyl donors perform better than the 4-Cl donor **10**, whereas the 4-OMe donor **9** performs as well as the 4-OAc analogues. In addition, of all of the galactosyl donors employed thus far in such reactions, the 4-fluorinated analogue seemed to perform the best. Thus the reactivity of galactosyl trichloroacetimidate donors towards the BF_3_·OEt_2_-promoted glycosylation at O-4 of *N*-acetylglucosamine glycosyl acceptors seems to follow the ranking 4-F > 4-OAc ≈ 4-OMe > 4-Cl.

### Preparation of the protected Le^x^ trisaccharide analogues

**Scheme 2:**
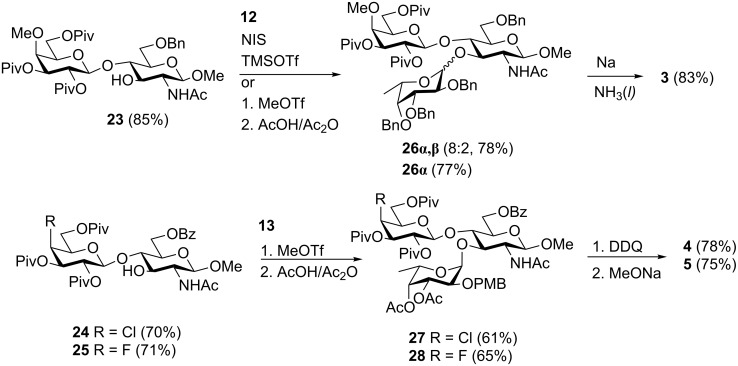
Synthesis of trisaccharides **26**–**28** and deprotection reactions giving **3**–**5**.

The synthesis of protected trisaccharide intermediates **26**–**28** is described in Scheme 2. First, acceptors **23**–**25** were prepared in good yields through the selective removal of the chloroacetate (thiourea) in disaccharides **20**–**22**. Fucosylation of acceptor **23** with ethylthioglycoside **12** was first attempted under NIS and TMSOTf activation at low temperature (−30 °C). Under these conditions, the desired trisaccharide **26** was isolated in 78% yield but as an 8:2 mixture of the α and β-anomers as estimated by ^1^H NMR. Although the desired anomer **26α** could be obtained pure upon purification by HPLC, it was isolated in a less than desirable yield of 48%. We thus attempted the coupling of acceptor **23** and donor **12** under activation with excess MeOTf (5 equiv). Indeed, we have reported that such conditions allow glycosylation at O-4 of *N*-acetylglucosamine acceptors through the in situ formation of the corresponding *N*-methylimidate, temporarily masking the *N*-acetyl group in the acceptor [70–71]. Thus, we expected a similar in situ formation of the methyl imidate in acceptor **23**, which would further undergo fucosylation at O-3. However, since methylimidates are unstable when purified on silica gel, they were converted back to the acetamido before purification. Thus, once TLC had shown that all of the acceptor had been consumed, the reaction was worked up and the crude mixture was treated with Ac_2_O–AcOH prior to purification by chromatography [70–71]. Under these conditions, the desired trisaccharide **26α** was obtained pure and free of the β-anomer in 77% yield.

Similar reaction conditions were applied for the glycosylation of acceptors **24** and **25** with ethylthioglycoside **13**. Interestingly, these fucosylation reactions proved to be slower and required additional equivalents of donor **13** to proceed. However, after treatment of the reaction mixtures with AcOH–Ac_2_O, the desired trisaccharides **27** and **28** were isolated in good yields (Scheme 2) and exclusively as the α-fucosylated trisaccharides. As previously observed for other similar analogues [66,72–73], careful analysis of the ^1^H NMR spectra acquired for trisaccharides **26α**, **27**, **28** indicated that the glucosamine residue adopted a conformation distorted from the usual ^4^C_1_ chair conformation. The distorted conformations of the *N*-acetylglucosamine ring in analogues **26α**, **27**, **28** were characterized by vicinal coupling constants of 6.2–6.6 Hz measured between the ring hydrogens H-2 to H-5 of this residue, rather than the expected values of 8.0–8.3 Hz as observed, when measurable, for the same hydrogens in disaccharides **20**–**25**. In addition, although signal overlap precluded its measurement in trisaccharides **26α** and **28**, the vicinal coupling constant measured between H-1 and H-2 in trisaccharide **27** (5.2 Hz) also supported a distorted conformation for this residue (compare to the same coupling constant in compounds **23**–**25**, ~7.4 Hz).

### Deprotection of trisaccharides **26**–**28**

As described previously, the removal of various protecting groups, such as pivaloyl and benzyl groups here, can be accomplished efficiently in one step under Birch reduction conditions [15,68–69,74–75]. Thus, treatment of trisaccharide **26α** with sodium in liquid ammonia at −78 °C was followed by neutralization of the reaction mixture with AcOH and purification by gel permeation chromatography on a Biogel P2 column (water) to give the desired deprotected 4”-methoxy trisaccharide analogue **3** pure in 83% yield. Since such conditions were not compatible with the 4-chloro and 4-fluoro substituents in trisaccharides **27** and **28**, these intermediates were converted in two steps to the desired deprotected analogues **4** and **5**, respectively. Thus, removal of the *p*-methoxybenzyl group with DDQ in CH_2_Cl_2_/H_2_O (15:1 v/v) was followed by Zemplén deacylation, giving the target Le^x^ analogues **4** and **5** in 78% and 75%, respectively, over the two steps. The structures of the final deprotected trisaccharides **3**–**5** were confirmed by HR–ESI mass spectrometry and NMR.

## Conclusion

We describe here the efficient synthesis of three Le^x^ methyl glycoside derivatives (**3**–**5**) in which the galactosyl 4-hydroxy group is either methylated (**3**) or replaced by a chlorine (**4**) or fluorine (**5**). Our results seem to indicate that galactosylation at O-4 of an *N*-acetylglucosamine acceptor under activation with excess BF_3_·OEt_2_ can be significantly affected by the nature of the substituent present at C-4 of the galactosyl donor. Indeed, the best results were obtained with the 4-fluoro galactosyl donor, whereas the 4-chloro donor reacted less efficiently than the 4-O-methyl or 4-O-acetyl donors. Overall, this study also confirms our observation [68], that galactosylations at position 4 of *N*-acetylglucosamine acceptors are usually less successful than glucosylations [14,67]. The final Le^x^-OMe analogues will be used as competitive inhibitors in future ELISA experiments to provide a better understanding of the binding process between the anti-Le^x^ monoclonal antibody SH1 and the Le^x^ antigen.

## Experimental

**General Methods: **^1^H (400.13 MHz) and ^13^C NMR (100.6 MHz) spectra were recorded for compounds solubilized in CDCl_3_ (internal standard, for ^1^H: residual CHCl_3_ δ 7.24; for ^13^C: CDCl_3_ δ 77.0), DMSO-*d*_6_ (internal standard, for ^1^H: residual DMSO δ 2.54; for ^13^C: DMSO-*d*_6_ δ 40.45), CD_3_OD (internal standard, for ^1^H: residual MeOD δ 3.31; for ^13^C: CD_3_OD δ 49.15) or D_2_O [external standard 3-(trimethylsilyl)propionic acid-*d*_4_, sodium salt (TSP) for ^1^H δ 0.00; for ^13^C δ 0.00]. Chemical shifts and coupling constants were obtained from a first-order analysis of one-dimensional spectra. Assignments of proton and carbon resonances were based on COSY and ^13^C–^1^H heteronuclear correlated experiments. ^1^H NMR data are reported with standard abbreviations: singlet (s), doublet (d), triplet (t), doublet of doublet (dd), doublet of doublet of doublet (ddd), broad singlet (bs), broad triplet (bt), broad doublet of doublet (bdd) and multiplet (m). Mass spectra were obtained with electrospray ionization (ESI) on a high-resolution mass spectrometer. TLC were performed on precoated aluminum plates with Silica Gel 60 F_254_ and detected with UV light and/or by charring with a solution of 10% H_2_SO_4_ in EtOH. Compounds were purified by flash chromatography with Silica Gel 60 (230–400 mesh) unless otherwise stated. Solvents were distilled and dried according to standard procedures [76], and organic solutions were dried over Na_2_SO_4_ and concentrated under reduced pressure below 40 °C. HPLC purifications were run with HPLC grade solvents.

**Methyl 2-acetamido-2-deoxy-3-*O*-(α-L-fucopyranosyl)-4-*O*-(4-methoxy-β-D-galactopyranosyl)-β-D-glucopyranoside (3).** A solution of the protected trisaccharide **26α** (50 mg, 0.043 mmol) dissolved in THF (5 mL) was added to a solution of liquid NH_3_ (ca. 20 mL) containing Na (72 mg, 3.13 mmol, 73 equiv) at −78 °C. After 1 h the reaction was quenched with MeOH (5 mL) and the ammonia was allowed to evaporate at rt. The remaining solution was neutralized with AcOH (220 μL, ca. 1.2 equiv to Na), and the mixture was concentrated. The resulting solid was dissolved in water and passed through a Biogel P2 column eluted with H_2_O. After freeze drying, the deprotected 4”-methoxy-Le^x^ analogue **3** (20 mg, 0.0359 mmol, 83%) was obtained pure as a white solid. [α]_D_ = −75 (*c* 0.3, H_2_O); ^1^H NMR (400 MHz, D_2_O, 295 K) δ 4.95 (d, *J* = 4.0 Hz, 1H, H-1’), 4.67 (m, 1H, H-5’), 4.32 (d, *J* = 8.2 Hz, 1H, H-1), 4.27 (d, *J* = 7.7 Hz, 1H, H-1”), 3.85 (dd, *J* = 2.0, 12.2 Hz, 1H, H-6a), 3.78–3.68 (m, 5H, H-2, H-3, H-4, H-3’, H-6b), 3.63–3.60 (m, 3H, H-4’, H-6ab”), 3.55–3.50 (m, 2H, H-2’, H-3”), 3.47–3.44 (m, 2H, H-5, H5”), 3.39 (d, *J* = 3.4 Hz, 1H, H-4”), 3.35 (s, 3H, OCH_3_), 3.32 (s, 3H, OCH_3_), 3.29 (m, 1H, H-2”), 1.88 (s, 3H, C(O)CH_3_), 1.03 (d, *J* = 6.6 Hz, 3H, H-6’); ^13^C NMR (100 MHz, D_2_O, 295 K) δ 174.4 (C=O), 101.7 (C-1), 101.6 (C-1”), 98.7 (C-1’), 78.6 (C-4”), 75.3, 75.1, 75.0 (C-5, C-5”, C-3), 73.3 (C-4), 72.9 (C-3”), 71.9 (C-4’), 71.5 (C-2”), 69.2 (C-3’), 67.7 (C-2’), 66.7 (C-5’), 61.1 (OCH_3_), 61.0 (C-6”), 59.7 (C-6), 57.1 (OCH_3_), 55.6 (C-2), 22.2 (C(O)*C*H_3_), 15.2 (C-6’); HRMS–ESI (*m*/*z*): [M + Na]^+^ calcd for C_22_H_39_NNaO_15_, 580.2217; found, 580.2223.

**Methyl 2-acetamido-2-deoxy-4-*O*-(4-chloro-4-deoxy-β-D-galactopyranoside)-3-*O*-(α-L-fucopyranosyl)-β-D-glucopyranoside (4).** A solution of the protected trisaccharide **27** (39 mg, 0.0347 mmol) and DDQ (12 mg, 1.5 equiv) in CH_2_Cl_2_ (350 μL) and H_2_O (20 μL, 6% v/v) was stirred at room temperature for 2 h. The mixture was filtered over Celite and the solids were washed with CH_2_Cl_2_ (5 mL). The combined filtrate and washings were washed with aq saturated NaHCO_3_ (10 mL), the aq layer was re-extracted with CH_2_Cl_2_ (3 × 5 mL), and the combined organic layers were dried and concentrated. Flash chromatography (EtOAc/hexanes, 1:1 → 7:3) of the residue gave a white solid (27 mg, 0.0269 mmol, 78%), which was dissolved in a methanolic solution of NaOMe (1 mL, 0.13 M) and stirred for 3 h at 50 °C. The reaction mixture was diluted with MeOH (5 mL) and de-ionized with DOWEX H^+^ resin. The resin was filtered off and washed with MeOH (5 mL), and the combined filtrated and washings were concentrated. The crude product was dissolved in Milli Q water and washed with CH_2_Cl_2_ (5 mL). After freeze drying, the deprotected 4”-deoxychloro Le^x^ analogue **4** (15.1 mg, 0.0269 mmol, 78%) was obtained pure as an amorphous solid. [α]_D_ = −123 (*c* = 0.7, H_2_O); ^1^H NMR (400 MHz, D_2_O, 295 K) δ 4.97 (d, 1H, *J* = 4.0 Hz, H-1’), 4.61 (m, 1H, H-5’), 4.38–4.30 (m, 3H, H-1, H-1”, H-4”), 3.88 (dd, *J* = 1.9, 12.3 Hz, 1H, H-6a), 3.82 (dd, *J* = 3.8, 9.7 Hz, 1H, H-3”), 3.77–3.70 (m, 7H, H-2, H-3, H-4, H-6b, H-3’, H-4’, H-5”), 3.66 (dd, *J* = 7.1, 11.7 Hz, 1H, H-6a”), 3.59–3.53 (m, 2H, H-2’, H-6b”), 3.45 (m, 1H, H-5), 3.38–3.33 (m, 4H, H-2”, OCH_3_), 1.88 (s, 3H, C(O)CH_3_), 1.05 (d, *J* = 6.6 Hz, 3H, H-6’); ^13^C NMR (100 MHz, D_2_O, 295 K) δ 174.4 (C=O), 102.2 (C-1”), 101.7 (C-1), 98.5 (C-1’), 75.2 (C-5), 74.4 (C-3), 73.7 (C-4), 73.6 (C-5”), 71.9 (C-4’), 71.5 (C-3”), 70.8 (C-2”), 69.3 (C-3’), 67.6 (C-2’), 66.6 (C-5’), 62.2 (C-4”), 61.6 (C-6”), 59.5 (C-6), 57.1 (OCH_3_), 55.7 (C-2), 22.2 (C(O)CH_3_), 15.2 (C-6’); HRMS–ESI (*m*/*z*): [M + Na]^+^ calcd for C_21_H_36_ClNNaO_14_, 584.1722; found, 584.1733.

**Methyl 2-acetamido-2-deoxy-4-*O*-(4-deoxy-4-fluoro-β-D-galactopyranoside)-3-*O*-(α-L-fucopyranosyl)-β-D-glucopyranoside (5).** Trisaccharide **28** (30 mg, 0.0271 mmol) was deprotected in two steps as described above for the preparation of trisaccharide **4**. After freeze drying, the deprotected 4”-deoxyfluoro Le^x^ analogue **5** (11.1 mg, 0.0203 mmol, 75%) was obtained pure as an amorphous solid. [α]_D_ = −85 (*c* 0.5, H_2_O); ^1^H NMR (400 MHz, D_2_O, 295 K) δ 4.95 (d, *J* = 4.0 Hz, 1H, H-1’), 4.66 (m, 1H, H-5’), 4.65 (bdd, *J* = 2.7 Hz, *J_H,F_* = 50.4 Hz, 1H, H-4”), 4.39 (d, *J* = 7.8 Hz, 1H, H-1”), 4.33 (d, *J* = 8.0 Hz, 1H, H-1), 3.86 (dd, *J* = 2.0, 12.3 Hz, 1H, H-6a), 3.81–3.66 (m, 6H, H-2, H-3, H-4, H-6b, H-3’, H-3”), 3.64–3.58 (m, 4H, H-4’, H-5”, H-6ab”), 3.54 (dd, *J* = 4.0, 10.4 Hz, 1H, H-2’), 3.45 (m, 1H, H-5), 3.37–3.33 (m, 4H, H-2”, OCH_3_), 1.88 (s, 3H, C(O)CH_3_), 1.02 (d, *J* = 6.6 Hz, 3H, H-6’); ^13^C NMR (100 MHz, D_2_O, 295 K) δ 174.4 (C=O), 101.7 (C-1), 101.4 (C-1”), 98.7 (C-1’), 89.3 (d, *J_C,F_* = 177.7 Hz, C-4”), 75.2 (C-5), 74.9 (C-3), 73.4 (C-4), 73.2 (d, *J_C,F_* 17.6 Hz, C-5”), 71.9 (C-4’), 71.2 (C2”), 71.1 (d, *J_C,F_* = 18.5 Hz, C-3”), 69.2 (C-3’), 67.6 (C-2’), 66.5 (C-5’), 60.0 (C-6”), 59.6 (C-6), 57.1 (OCH_3_), 55.6 (C-2), 22.2 (C(O)*C*H_3_), 15.3 (C-6’); HRMS–ESI (*m*/*z*): [M + Na]^+^ calcd for C_21_H_36_FNO_14_, 568.2018; found, 568.2023.

**Methyl 2-acetamido-6-*O*-benzyl-3-*O*-chloroacetyl-2-deoxy-4-*O*-(4-*O*-methyl-2,3,6-tri-*O*-pivaloyl-β-D-galactopyranosyl)-β-D-glucopyranoside (20).** A solution of acceptor **6** (215 mg, 0.535 mmol) and trichloroacetimidate donor **9** (1.58 g, 5.0 equiv) in CH_2_Cl_2_ (30 mL) was stirred at 40 °C, and BF_3_·OEt_2_ (134 μL, 2.0 equiv) was added. The reaction was allowed to proceed for 2 h at 40 °C and then quenched with Et_3_N (179 μL, 2.4 equiv), and the mixture was diluted with CH_2_Cl_2_ (70 mL). The mixture was washed with aq saturated NaHCO_3_ (100 mL), the aq layer was re-extracted with CH_2_Cl_2_ (20 mL × 3), and the combined organic layers were dried and concentrated. Flash chromatography (EtOAc/hexanes, 2:8 → 6:4) of the residue gave disaccharide **20** (312 mg, 0.375 mmol, 70%) pure as a colourless glass. [α]_D_ = −11 (*c* 0.6, CH_2_Cl_2_); ^1^H NMR (400 MHz, CDCl_3_, 296 K) δ 7.29 (m, 5H, H_arom_), 5.95 (d, *J* = 9.2 Hz, 1H, NH), 5.08–4.99 (m, 2H, H-3, H-2’), 4.71–4.68 (m, 2H, H-3’, C*H*HPh), 4.40 (d, *J* = 12.1 Hz, 1H, C*H*HPh), 4.38 (d, *J* = 7.4 Hz, 1H, H-1), 4.24–4.19 (m, 2H, H-1’, H-6a’), 4.13–4.07 (m, 2H, H-6b’, C*H*HCl), 4.01 (d, *J* = 15.1 Hz, 1H, C*H*HCl), 3.96–3.92 (m, 2H, H-4, H-2), 3.71 (m, 2H, H-6ab), 3.50–3.40 (m, 3H, H-5, H-4’, H-5’), 3.43, 3.41 (2s, 6H, 2 × OCH_3_), 1.94 (s, 3H, C(O)CH_3_), 1.30, 1.15, 1.10 (3s, 27H, 3 × C(CH_3_)_3_); ^13^C NMR (100 MHz, CDCl_3_, 296 K) δ 177.9, 177.7, 176.2, 170.3, 167.3 (C=O), 137.7, 128.6, 128.1, 128.0 (Ar), 101.7 (C-1), 99.2 (C-1’), 76.3 (C-4’), 74.2 (C-5), 73.9 (C-3), 73.5 (*C*H_2_Ph), 73.4 (C-3’), 72.1 (C-4), 72.0 (C-5’), 69.5 (C-2’), 67.7 (C-6), 61.7 (C-6’), 61.5 (OCH_3_), 56.6 (OCH_3_), 52.6 (C-2), 40.8 (CH_2_Cl), 38.8, 38.7, 38.6 (*C*(CH_3_)_3_), 27.2, 27.1 (C(*C*H_3_)_3_), 23.3 (C(O)*C*H_3_); HRMS–ESI (*m*/*z*): [M + H]^+^ calcd for C_40_H_61_ClNO_15_, 830.3730; found, 830.3735.

**Methyl 2-acetamido-6-*O*-benzoyl-3-*O*-chloroacetyl-4-*O*-(4-chloro-4-deoxy-2,3,6-tri-*O*-pivaloyl-β-D-galactopyranosyl)-2-deoxy-β-D-glucopyranoside (21).** Glycosylation of acceptor **8** (97 mg, 0.233 mmol) with trichloroacetimidate **10** (694 mg, 5.0 equiv) was performed under BF_3_·OEt_2_ (59 μL, 2.0 equiv) activation as described above for the synthesis of disaccharide **20**. Work-up, as described above, and flash chromatography (EtOAc/hexanes, 2:8 → 6:4) of the residue gave disaccharide **21** (125 mg, 0.147 mmol, 63%) pure as a colourless glass. [α]_D_ = +9 (*c* 0.9, CH_2_Cl_2_); ^1^H NMR (400 MHz, CDCl_3_, 295 K) δ 8.00–7.41 (m, 5H, Ar), 5.87 (d, *J* = 9.3 Hz, 1H, NH), 5.27–5.16 (m, 2H, H-2’, H-3), 4.86 (dd, *J* = 3.9, 10.1 Hz, 1H, H-3’), 4.61 (dd, *J* = 2.9, 12.0 Hz, 1H, H-6a), 4.51–4.47 (m, 3H, H-1, H-6b, H-1’), 4.38 (d, *J* = 3.5 Hz, 1H, H-4’), 4.35–4.30 (dd, *J* = 7.2, 11.5 Hz, 1H, H-6a’), 4.16–4.02 (m, 4H, H-2, H-6b’, CH_2_CCl_3_), 3.91 (t, *J* = 8.3 Hz, 1H, H-4), 3.83 (bt, *J* = 6.2 Hz, 1H, H-5’), 3.72 (m, 1H, H-5), 3.45 (s, 3H, OCH_3_), 1.97 (s, 3H, C(O)CH_3_), 1.16, 1.14, 1.13 (3s, 27H, 3 × C(CH_3_)_3_); ^13^C NMR (100 MHz, CDCl_3_, 295 K) δ 177.8, 177.6, 176.3, 170.3, 167.3, 166.0 (C=O), 133.5, 129.6, 129.4, 128.7, 128.4 (Ar), 101.7 (C-1), 100.4 (C-1’), 73.5 (C-3), 73.4 (C-4), 72.5 (C-5), 71.6 (C-3’), 71.2 (C-5’), 68.3 (C-2’), 62.6 (C-6), 62.6 (C-6’), 57.2 (C-4’), 56.8 (OCH_3_), 52.6 (C-2), 40.7 (CH_2_Cl), 38.9, 38.8, 38.7 (*C*(CH_3_)_3_), 27.6, 27.1, 27.0, 26.9, 26.7 (C(*C*H_3_)_3_), 23.3 (C(O)*C*H_3_); HRMS–ESI (*m*/*z*): [M + H]^+^ calcd for C_39_H_56_Cl_2_NO_15_, 848.3027; found, 848.3009.

**Methyl 2-acetamido-6-*O*-benzoyl-3-*O*-chloroacetyl-2-deoxy-4-*O*-(4-deoxy-4-fluoro-2,3,6-tri-*O*-pivaloyl-β-D-galactopyranosyl)-β-D-glucopyranoside (22).** Glycosylation of acceptor **8** (91.5 mg, 0.220 mmol) with trichloroacetimidate **11** (637 mg, 5.0 equiv) was performed under BF_3_·OEt_2_ (134 μL, 2.0 equiv) activation as described above for the synthesis of disaccharide **20**. Work-up, as described above, and flash chromatography (EtOAc/hexanes, 2:8 → 6:4) of the residue gave disaccharide **22** (143 mg, 0.172 mmol, 77%) pure as a colourless glass. [α]_D_ = +9 (*c* 2.2, CH_2_Cl_2_); ^1^H NMR (400 MHz, CDCl_3_, 295 K) δ 7.98–7.46 (m, 5H, H_arom_), 6.00 (d, *J* = 9.3 Hz, 1H, NH), 5.21–5.16 (m, 2H, H-3, H-2’), 4.82 (ddd, *J* = 2.6, 10.3 Hz, *J_H,F_* = 26.9 Hz, 1H, H-3’), 4.70 (dd, *J* = 2.6 Hz, *J_H,F_* = 42.9 Hz, 1H, H-4’), 4.62 (m, 1H, H-6a), 4.51–4.46 (m, 3H, H-1, H-6b, H-1’), 4.29 (dd, *J* = 7.6, 11.4 Hz, 1H, H-6a’), 4.16 (dd, *J* = 5.6, 11.5 Hz, 1H, H-6b’), 4.09–4.00 (m, 3H, H-2, CH_2_Cl), 3.92 (t, *J* = 8.2 Hz, 1H, H-4), 3.72 (m, 1H, H-5), 3.66 (dt, *J* = 6.4 Hz, *J_H,F_* = 25.8 Hz, 1H, H-5’), 3.44 (s, 3H, OCH_3_), 1.97 (s, 3H, OCH_3_), 1.16, 1.13 (2s, 27H, 3 × C(CH_3_)_3_); ^13^C NMR (100 MHz, CDCl_3_, 295 K) δ 177.8, 177.6, 176.5, 170.46, 167.3, 165.9 (C=O), 133.5, 129.5, 129.4, 128.6 (Ar), 101.6 (C-1), 99.9 (C-1’), 85.3 (d, *J_C,F_* = 186.4 Hz, C-4’), 73.5 (C-3), 73.4 (C-4), 72.4 (C-5), 71.2 (d, *J_C,F_* = 18.0 Hz, C-3’), 71.1 (d, *J_C,F_* = 18.0 Hz, C-5’), 68.6 (C-2’), 62.6 (C-6), 61.2 (C-6’), 56.8 (OCH_3_), 52.5 (C-2), 40.6 (CH_2_Cl), 38.8, 38.8, 38.7 (*C*(CH_3_)), 27.1, 26.9 (C(*C*H_3_)), 23.2 (C(O)*C*H_3_); HRMS–ESI (*m*/*z*): [M + H]^+^ calcd for C_39_H_56_ClFNO_15_, 832.3323; found, 832.3344.

**Methyl 2-acetamido-6-*O*-benzyl-3-*O*-(2,3,4-tri-*O*-benzyl-α-L-fucopyranosyl)-2-deoxy-4-*O*-(4-*O*-methyl-2,3,6-tri-*O*-pivaloyl-β-D-galactopyranosyl)-β-D-glucopyranoside (26).** A mixture of disaccharide acceptor **23** (30 mg, 0.0398 mmol), known [56] thioethyl fucopyranoside **12** (76 mg, 0.159 mmol, 4.0 equiv), and activated powdered 4 Å molecular sieves (0.25 g) in Et_2_O (3.0 mL, 0.13 M), was stirred for 1 h at rt under N_2_. Then, MeOTf (23 μL, 5.0 equiv) was added, the reaction mixture was stirred for 30 min, and the reaction quenched with Et_3_N (33 μL, 6.0 equiv). Solids were filtered off on Celite and washed with CH_2_Cl_2_ (20 mL), and the combined filtrate and washings were washed with aq saturated NaHCO_3_ (15 mL). The aq layer was re-extracted with CH_2_Cl_2_ (3 × 10 mL), and the combined organic layers were dried and concentrated. The residue was dissolved in 25% AcOH in Ac_2_O (5 mL), and the solution was stirred at rt for 12 h and co-concentrated with toluene (3 × 10 mL). Flash chromatography (EtOAc/hexanes, 2:8 → 1:1) of the residue gave trisaccharide **26** (35.7 mg, 0.0305 mmol, 77%) pure as a colourless glass. [α]_D_ = −56 (*c* 0.7, CH_2_Cl_2_); ^1^H NMR (400 MHz, CDCl_3_, 296 K) δ 7.32–7.17 (m, 20H, H_arom_), 5.96 (d, *J* = 7.6 Hz, 1H, NH), 5.14 (dd, *J* = 8.1, 10.4 Hz, 1H, H-2”), 5.04 (d, *J* = 3.6 Hz, 1H, H-1’), 4.89 (d, *J* = 11.7 Hz, 1H, C*H*HPh), 4.78–4.66 (m, 6H, H-1, H-3”, 2 × C*H_2_*Ph), 4.60 (d, *J* = 11.7 Hz, 1H, C*H*HPh), 4.56 (d, *J* = 12.1 Hz, 1H, C*H*HPh), 4.31 (m, 2H, H-1”, C*H*HPh), 4.21 (m, 1H, H-5’), 4.10–4.00 (m, 4H, H-4, H-2’, H-6ab”), 3.89 (t, *J* = 6.3 Hz, 1H, H-3), 3.86 (dd, *J* = 2.6, 10.1 Hz, 1H, H-3’), 3.79 (dd, *J* = 4.9, 10.1 Hz, 1H, H-6a), 3.69 (dd, *J* = 3.7, 10.1 Hz, 1H, H-6b), 3.59 (bd, *J* = 1.4 Hz, 1H, H-4’), 3.46–3.40 (m, 4H, H-2, H-5, H-4”, H-5”), 3.29, 3.26 (2s, 6H, 2 × OCH_3_), 1.73 (s, 3H, C(O)CH_3_), 1.14–1.08 (m, 30H, H-6’, 3 × C(O)C(CH_3_)_3_); ^13^C NMR (100 MHz, CDCl_3_, 296 K) δ 177.7, 177.6, 176.8, 170.3 (C=O), 139.1, 139.0, 138.7, 138.0, 128.5–127.0 (Ar), 100.4 (C-1), 98.8 (C-1”), 96.2 (C-1’), 79.7 (C-3’), 77.0 (C-4’), 76.5 (C-4), 76.2 (C-4”), 74.7 (*C*H_2_Ph), 73.4 (C-5”), 73.3 (C-3”), 73.0, 72.6, 72.2 (3 × *C*H_2_Ph), 72.0 (C-3), 71.8 (C-2’), 71.6 (C-5), 69.1 (C-2”), 68.8 (C-6), 66.6 (C-5’), 61.5 (C-6”), 61.3 (OCH_3_), 56.5 (OCH_3_), 53.5 (C-2) 38.8, 38.7 (*C*(CH_3_)_3_), 27.2, 27.1 (C(*C*H_3_)_3_), 23.1 (C(O)*C*H_3_), 16.6 (C-6’); HRMS–ESI (*m*/*z*): [M + H]^+^ calcd for C_65_H_88_NO_18_, 1170.6001; found, 1170.6033.

**Methyl 2-acetamido-3-*O*-(3,4-acetyl-2-*O*-paramethoxybenzyl-α-L-fucopyranosyl)-6-*O*-benzoyl-4-*O*-(4-chloro-4-deoxy-2,3,6-pivaloyl-β-D-galactopyranosyl)-2-deoxy-β-D-glucopyranoside (27).** A mixture of disaccharide acceptor **24** (48 mg, 0.0622 mmol), known [12] thiophenyl fucopyranoside **13** (86 mg, 0.187 mmol, 3.0 equiv) and activated powdered 4 Å molecular sieves (0.3 g) in Et_2_O (2.0 mL) was stirred 1 h at rt under N_2_. MeOTf (35 μL, 5.0 equiv) was added and the reaction was allowed to proceed for 3 h at rt. More donor **13** (43 mg, 1.5 equiv) was added and the reaction was allowed to proceed for an additional 1 h at rt before being quenched with Et_3_N (52 μL, 6.0 equiv). Work up of the reaction and treatment of the crude product in 25% AcOH in Ac_2_O (6 mL), as well as the subsequent work-up, were carried out as described above for the synthesis of trisaccharide **26.** Flash chromatography (EtOAc/hexanes, 2:8 → 6:4) of the residue gave trisaccharide **27** (42.5 mg, 0.0379 mmol, 61%) pure as a colourless glass. [α]_D_ = –21 (*c* 0.8, CH_2_Cl_2_); ^1^H NMR (400 MHz, CDCl_3_, 295 K) δ 8.00–6.84 (m, 9H, H_arom_), 6.01 (d, *J* = 7.6 Hz, 1H, NH), 5.32–5.24 (m, 3H, H-3’, H-4’, H-2”), 5.18 (d, *J* = 3.6 Hz, 1H, H-1’), 4.90 (dd, *J* = 3.8, 10.0 Hz, 1H, H-3”), 4.80 (d, *J* = 5.2 Hz, 1H, H-1), 4.72 (dd, *J* = 3.6, 11.9 Hz, 1H,H-6a), 4.65–4.52 (m, 5H, H-6b, H-5’, H-1”, C*H_2_*Ph), 4.42 (bd, *J* = 3.4 Hz, 1H, H-4”), 4.39–4.34 (m, 2H, H-6ab”), 4.20 (t, *J* = 6.6 Hz, 1H, H-3), 3.96 (t, *J* = 6.4 Hz, 1H, H-4), 3.89 (dd, *J* = 3.7, 10.4 Hz, 1H, H-2’), 3.81–3.76 (m, 2H, H-5, H-5”), 3.76 (s, 3H, OCH_3_), 3.58 (m, 1H, H-2), 3.34 (s, 3H, OCH_3_), 2.11, 1.95, 1.86 (3s, 9H, 3 × C(O)CH_3_), 1.15–1.14 (m, 30H, H-6’, 3 × C(CH_3_)_3_); ^13^C NMR (100 MHz, CDCl_3_, 295 K) δ 177.6, 177.5, 170.5, 170.4, 169.7, 166.0, 159.2 (C=O), 133.4, 130.4, 129.7, 129.5, 128.9, 128.7 (Ar), 100.4 (C-1), 100.0 (C-1”), 96.1 (C-1’), 73.3 (C-4, C-2’), 72.6 (*C*H_2_Ph), 72.2 (C-5”), 71.7 (C-4’, C-3, C-5), 71.5 (C-3”), 70.4 (C-3’), 68.0 (C-2”), 65.0 (C-5’), 63.7 (C-6), 62.2 (C-6”), 57.6 (C-4”), 56.6, 55.3 (OCH_3_), 53.5 (C-2) 38.9, 38.8, 38.7 (*C*(CH_3_)_3_), 27.1, 27.0, 27.0 (C(*C*H_3_)_3_), 23.2, 20.9, 20.7 (C(O)*C*H_3_), 15.9 (C-6’); HRMS–ESI (*m*/*z*): [M + H]^+^ calcd for C_55_H_77_ClNO_21_, 1122.4677; found, 1122.4626.

**Methyl 2-acetamido-3-*O*-(3,4-di-*O*-acetyl-2-*O*-*p*-methoxybenzyl-α-L-fucopyranosyl)-6-*O*-benzoyl-2-deoxy-4-*O*-(4-deoxy-4-fluoro-2,3,6-pivaloyl-β-D-galactopyranosyl)-β-D-glucopyranoside (28).** A mixture of disaccharide acceptor **25** (24 mg, 0.0318 mmol), known [12] thiophenyl fucopyranoside **13** (44 mg, 0.0953 mmol, 3.0 equiv) and activated powdered 4 Å molecular sieves (0.15 g) in Et_2_O (1.5 mL) was stirred for 1 h at rt under N_2_. MeOTf (18 μL, 5.0 equiv) was added and the reaction was allowed to proceed for 30 min at rt. More donor **13** (44 mg, 3.0 equiv) was added and the reaction was allowed to proceed for an additional 2 h at rt before being quenched with Et_3_N (27 μL, 6.0 equiv). Work up of the reaction and treatment of the crude product in 25% AcOH in Ac_2_O (4 mL), as well as the subsequent work-up, were carried out as described above for the synthesis of trisaccharide **26.** Flash chromatography (EtOAc/hexanes, 2:8 → 6:4) of the residue gave trisaccharide **28** (22.8 mg, 0.0206 mmol, 65%) pure as a colourless glass. [α]_D_ = −37 (*c* 1.2, CH_2_Cl_2_); ^1^H NMR (400 MHz, CDCl_3_, 295 K) δ 8.00–6.84 (m, 9H, H_arom_), 6.08 (d, *J* = 7.9 Hz, 1H, NH), 5.30–5.22 (m, 3H, H-3’, H-4’, H-2”), 5.19 (d, *J* = 3.6 Hz, 1H, H-1’), 4.87 (ddd, *J* = 2.4, 10.3 Hz, *J_H,F_* = 27.1 Hz, 1H, H-3”), 4.79–4.53 (m, 7H, H-1, H-6ab, H-1”, H-4”, C*H*_2_Ph), 4.47 (m, 1H, H5’), 4.36–4.33 (m, 2H, H-6ab”), 4.14 (t, *J* = 6.2 Hz, 1H, H-3), 3.97 (t, *J* = 6.1 Hz, 1H, H-4), 3.90–3.83 (m, 2H, H-5, H-2’), 3.76 (s, 3H, OCH_3_), 3.69–3.61 (m, 2H, H-2, H-5”), 3.34 (s, 3H, OCH_3_), 2.11, 1.95, 1.87 (3s, 9H, 3 × C(O)CH_3_), 1.17–1.14 (m, 30H, H-6’, 3 × C(CH_3_)_3_); ^13^C NMR (100 MHz, CDCl_3_, 295 K) δ 177.6, 177.0, 170.5, 170.4, 169.8, 166.0, 159.2 (C=O), 133.3, 130.3, 129.7, 129.5–128.6, 113.8 (Ar), 100.5 (C-1’), 99.2 (C-1”), 95.9 (C-1), 85.4 (d, *J_C,F_* = 186.3 Hz, C-4”), 73.3 (C-2’), 72.9 (C-4), 72.6 (CH_2_Ph), 72.1 (C-3), 71.6 (C-5), 71.1 (d, *J_C,F_* = 18.1 Hz, C-5”), 70.9 (d, *J_C,F_* = 18.5 Hz , C-3”), 70.5 (C-3’, C-4’), 68.5 (C-2”), 64.9 (C-5’), 63.8 (C-6), 60.7 (C-6”), 56.6, 55.2 (OCH_3_), 52.7 (C-2), 38.9, 38.8, 38.7 (*C*(CH_3_)_3_), 23.1 (C(*C*H_3_)_3_), 20.9, 20.7 (C(O)*C*H_3_), 15.8 (C-6’); HRMS–ESI (*m*/*z*): [M + H]^+^ calcd for C_55_H_77_FNO_21_, 1106.4972; found, 1106.4956.

## Supporting Information

File 1 Experimental procedures and characteristics for compounds **8**–**11**, **14**–**19**, **23**–**25**.

File 2 ^1^H NMR and ^13^C NMR for compounds **3**–**5**, **8**–**11**, **14**–**28**.
