# Supplementary material for: Synthesis of 4” manipulated Lewis X trisaccharide analogues
Source: Beilstein J Org Chem. 2012 Jul 23;8:1134–43. doi: 10.3762/bjoc.8.126 (PMC3458731; doi:10.3762/bjoc.8.126)
Supplement: File 1 — Experimental procedures and characteristics for compounds 8–11, 14–19, 23–25. [file Beilstein_J_Org_Chem-08-1134-s001.pdf]

Supporting Information

for

Synthesis of 4” manipulated Lewis X trisaccharide analogues

Christopher J. Moore and France-Isabelle Auzanneau*

Address: Department of Chemistry, University of Guelph, 50 Stone Rd. East, Guelph, Ontario, N1G 2W1, Canada

Email: France-Isabelle Auzanneau - fauzanne@uoguelph.ca

*Corresponding author

Experimental procedures and characteristics for compounds **8–11**, **14–19**, **23–25**.

Experimental

Methyl 2-acetamido-6-*O*-benzoyl-3-*O*-chloroacetyl-2-deoxy-β-D-glucopyranoside (8).

A mixture of known [1] methyl 2-acetamido-4,6-*O*-benzylidene-3-*O*-chloroacetyl-2-deoxy-β-D-glucopyranoside (500 mg, 1.25 mmol) **7** and 85% aq AcOH (25 mL) was stirred at 80 ºC for 2 h. The mixture was co-concentrated with toluene (3 × 20 mL) and the crude product was purified by flash chromatography (CH_2Cl_2/MeOH, 50:1 → 95:5) to give methyl 2-acetamido-3-*O*-chloroacetyl-2-deoxy-β-D-glucopyranoside (306 mg, 0.983 mmol, 79%). It was dissolved in CH_2Cl_2 (20 mL), and then collidine (391 µL, 3 equiv) and benzoyl chloride (137 µL, 1.2 equiv) were added, and the mixture was stirred for 24 h at rt. The solution was diluted with CH_2Cl_2 (30 mL), washed with 2 M HCl (30 mL) and saturated aq $NaHCO_3$ (30 mL), and dried. The solvents were evaporated, and the acceptor **8** was filtered off from Et_2O (20 mL) and obtained pure as a white amorphous powder (252 mg, 0.606 mmol, 62% over 2 steps). Mp = 220 °C. $[\alpha]_D$ −25 (*c* 1.0, MeOH). ^{1}H NMR (400 MHz, DMSO-d_6, 295 K): δ 7.96–7.55 (m, 5H, H_{arom}), 5.70 (d, 1H, *J* = 5.6 Hz, NH), 4.93 (t, 1H, *J* = 10.1 Hz, H-3), 4.55 (dd, 1H, *J* = 1.7, 12.0 Hz, H-6a), 4.45 (d, 1H, *J* = 8.4 Hz, H-1), 4.41 (dd, 1H, *J* = 5.3, 12.0 Hz, H-6b), 4.34, 4.21 (2d AB_{system}, 2H, *J* = 15.2 Hz, C(O)CH_2Cl), 3.74–3.65 (m, 2H, H-2, H-5), 3.56 (m, 1H, H-4), 3.31 (s, 3H, OCH_3), 1.74 (s, 3H, C(O)CH_3). ^{13}C NMR (100 MHz, DMSO-d_6, 295 K): δ 169.5, 167.2, 165.7 (C=O), 133.6, 129.7, 129.3, 129.0 (Ar), 101.2 (C-1), 77.9 (C-3), 73.3 (C-5), 68.0 (C-4), 63.6 (C-6), 56.1 (OCH_3), 53.0 (C-2), 41.2 (CH_2Cl), 22.8 (C(O)CH_3). HRMS–ESI Calcd for $C_{18}H_{22}ClNO_8$ $[M + H]^+$ 416.1112, found 416.1128.

4-*O*-Methyl-2,3,6-tri-*O*-pivaloyl-*α*-D-galactopyranosyl trichloroacetimidate (9). Galactopyranoside **16** (6.02 g, 10.4 mmol) was dissolved in AcOH (115 mL), and then activated powdered Zn (10.2 g, 15 equiv) and AcONa (8.5 g, 10 equiv) were added, and the mixture was

stirred at 40 °C for 1 h. The solids were filtered off, washed with CH_2Cl_2 (150 mL) and the combined filtrate and washing was poured onto ice-cold saturated aq $NaHCO_3$ (400 mL). The organic layer was collected and washed with aq saturated $NaHCO_3$ (200 mL × 2). The aq layers were re-extracted with CH_2Cl_2 (50 mL × 3) and the combined organic layers were dried and concentrated. Flash chromatography (EtOAc/hexanes, 2:8) gave the corresponding hemiacetal (3.99 g, 8.89 mmol, 85%) pure as a white foam. This was dissolved in CH_2Cl_2 (110 mL), and trichloroacetonitrile (4.46 mL, 5.0 equiv) was added followed by DBU (332 μL, 0.25 equiv). The reaction mixture was left at rt for 3 h and concentrated. The crude product was purified by flash chromatography (EtOAc/hexanes, 1:9 with 0.1% Et_3N) to give pure trichloroacetimidate **9** (4.26 g, 7.2 mmol, 69% over two steps) as a white foam. $[\alpha]_D$ = +85 (*c* 1.3, CH_2Cl_2). ^{1}H NMR (400 MHz, $CDCl_3$, 296 K): δ 8.58 (s, 1H, NH), 6.53 (d, 1H, *J* = 3.5 Hz, H-1), 5.47 (dd, 1H, *J* = 3.5, 10.8 Hz, H-2), 5.38 (dd, 1H, *J* = 2.2, 10.8 Hz, H-3), 4.26–4.19 (m, 3H, H-5, H-6ab), 3.78 (bd, 1H, *J* = 2.2 Hz, H-4), 3.49 (s, 3H, OCH_3), 1.15 (3s, 27H, 3 x $C(CH_3)_3$). ^{13}C NMR (100 MHz, $CDCl_3$, 296 K): δ 178.0, 177.6, 177.3 (C=O), 160.7 (C=NH), 93.6 (C-1), 91.0 (CCl_3), 77.3 (C-4), 71.1 (C-5), 70.3 (C-3), 67.2 (C-2), 62.5 (C-6), 61.6 (OCH_3), 38.9, 38.8, 38.7 ($\mathit{C}(CH_3)_3$), 27.1 ($C(\mathit{C}H_3)_3$). HRMS–ESI Calcd for $C_{24}H_{38}Cl_3NNaO_9$ $[M + Na]^+$ 612.1510, found 612.1514.

4-Chloro-4-deoxy-2,3,6-tri-*O*-pivaloyl-α-D-galactopyranosyl trichloroacetimidate (10). Galactopyranoside **18** (4.71 g, 8.09 mmol) was treated with zinc (6.35 g, 12 equiv), AcONa (5.98 g, 9.0 equiv) and AcOH (80 mL, 0.1 M) as described above for the conversion of glycoside **16** to the corresponding hemiacetal. Work-up and flash chromatography (EtOAc/hexanes, 2:8), as described above, gave the corresponding 4-chloro hemiacetal (2.83 g, 6.24 mmol, 77%) pure as a white foam. It was dissolved in CH_2Cl_2 (78 mL) and allowed to react with trichloroacetonitrile (3.13 mL, 5.0 equiv) and DBU (231 μL, 0.25 equiv), as described above for the preparation of

trichloroacetimidate **9**. Work-up (as described above) and flash chromatography (EtOAc/hexanes, 1:9, 0.1% Et_3N) gave trichloroacetimidate **10** pure (2.86 g, 4.81 mmol, 59% over two steps) as a white foam. $[\alpha]_D$ = +115 (*c* 1.1, CH_2Cl_2). ^{1}H NMR (400 MHz, $CDCl_3$, 295 K): δ 8.66 (s, 1H, NH), 6.58 (d, 1H, *J* = 3.6 Hz, H-1), 5.50 (dd, 1H, *J* = 3.7, 10.6 Hz, H-2), 5.37 (dd, 1H, *J* = 3.7, 10.6 Hz, H-3), 4.63 (bd, 1H, H-4), 4.52 (m, 1H, H-5), 4.28 (dd, 1H, *J* = 7.3, 11.8 Hz, H-6a), 4.19 (dd, 1H, *J* = 4.4, 11.8 Hz, H-6b), 1.17 (3s, 27H, 3 x $C(CH_3)$). ^{13}C NMR (100 MHz, $CDCl_3$, 295 K): δ 177.6 (C=O), 160.5 (C=NH), 93.1 (C-1), 91.0 (CCl_3), 69.5 (C-3), 68.3 (C-5), 66.2 (C-2), 63.7 (C-6), 58.1 (C-4), 38.9, 38.8, 38.7 ($\mathit{C}(CH_3)_3$), 27.1, 27.0 ($C(\mathit{C}H_3)_3$). HRMS–ESI Calcd for $C_{23}H_{35}Cl_4NNaO_8$ $[M + Na]^+$ 616.1014, found 616.1019.

4-Deoxy-4-fluoro-2,3,6-tri-*O*-pivaloyl-α-D-galactopyranosyl trichloroacetimidate (11). Galactopyranoside **19** (3.20 g, 5.65 mmol) was treated with Zn (4.44 g, 12 equiv), AcONa (4.17 g, 9.0 equiv) and AcOH (57 mL), as described above for the conversion of glycoside **16** to the corresponding hemiacetal. Work-up and flash chromatography (EtOAc/hexanes, 2:8) as described above, gave the corresponding 4-fluoro hemiacetal (2.18 g, 4.99 mmol, 88%) pure as a colourless foam. This was dissolved in CH_2Cl_2 (62 mL) and allowed to react with trichloroacetonitrile (2.50 mL, 5.0 equiv) and DBU (187 μL, 0.25 equiv), as described above for the preparation of trichloroacetimidate **9**. Work-up, as described above, and flash chromatography (EtOAc/hexanes, 5:95, 0.1% Et_3N) gave trichloroacetimidate **11** pure (1.99 g, 3.43 mmol, 61% over two steps) as a white foam. $[\alpha]_D$ = +109 (*c* 1.1, CH_2Cl_2). ^{1}H NMR (400 MHz, $CDCl_3$, 295 K): δ 8.65 (s, 1H, NH), 6.60 (d, 1H, *J* = 3.5 Hz, H-1), 5.46 (dd, 1H, *J* = 3.6, 10.0 Hz, H-2), 5.35 (ddd, 1H, *J* = 2.5, 10.8 Hz, $J_{H,F}$ = 25.9 Hz, H-3), 4.95 (bdd, 1H, *J* = 2.4 Hz, $J_{H,F}$ = 50.3 Hz, H-4), 4.30 (m, 1H, H-5), 4.27 (m, 2H, H-6ab), 1.16 (3s, 27H, 3 x $C(CH_3)_3$). ^{13}C NMR (100 MHz, $CDCl_3$, 295 K): δ 177.9, 177.7, 177.2 (C=O), 160.6 (C=NH), 93.2 (C-1), 91.0

(CCl_3), 86.5 (d, $J_{C,F}$ = 185.9 Hz, C-4), 69.6 (d, $J_{C,F}$ = 18.2 Hz, C-5), 67.9 (d, $J_{C,F}$ = 17.5 Hz, C-3), 66.4 (C-2), 62.0 (C-6), 39.0, 38.8, 38.7 ($C(CH_3)_3$), 27.1, 27.0 ($C(CH_3)_3$). HRMS–ESI Calcd for $C_{23}H_{35}Cl_3FNNaO_8$ $[M + Na]^+$ 600.1310, found 600.1317.

2,2,2-Trichloroethyl (α,β)-D-galactopyranoside (14). A mixture of D-galactose (10.0 g, 55.6 mmol), Ac_2O and pyridine (100 mL, 1:1) was stirred for 2 h at 50 °C. Solvents were co-evaporated with toluene (100 mL × 3) and the residue was diluted with CH_2Cl_2 (750 mL) and washed with 2 M aq HCl (250 mL) and saturated aq $NaHCO_3$ (250 mL). The aq layers were re-extracted with CH_2Cl_2 (200 mL), and the combined organic layers were dried and concentrated. The peracetylated galactose (21.7 g, 55.6 mmol) was dissolved in CH_2Cl_2 (140 mL) and $HOCH_2CCl_3$ (21.3 mL, 222 mmol, 4 equiv), the solution was heated to reflux, and $BF_3·OEt_2$ (20.9 mL, 169 mmol, 3 equiv) was added dropwise over 5 minutes. The reaction mixture was heated under reflux for 20 h, allowed to cool down to rt, diluted with CH_2Cl_2 (400 mL) and washed with aq saturated $NaHCO_3$ (250 mL × 3). The aq layers were re-extracted with CH_2Cl_2 (100 mL × 3), and the combined organic phases were dried and concentrated. Flash chromatography (EtOAc/hexanes, 25:75) gave the 2,2,2-trichloroethyl galactopyranoside intermediate as a pale yellow syrup (20.83 g, 43.4 mmol, 78%), which was then dissolved in a methanolic solution of MeONa (220 mL, 0.2 M). The solution was stirred at rt for 1 h, deionized with DOWEX® H^+ and the resin was filtered off and washed with MeOH (50 mL). The combined filtrate and washing were concentrated to give the anomeric mixture of the known [2] trichloroethyl glycoside **14α,β** pure as slightly orange foam (13.4 g, 43.4 mmol, 78%). ^1H NMR showed that the α/β ratio was 9:1. ^1H NMR for the major α anomer (400 MHz, CD_3OD, 295 K): δ 5.09 (d, 1H, J = 3.3 Hz, H-1), 4.34, 4.22 (2d AB_{system}, 2H, J = 11.5 Hz, CH_2CCl_3), 3.94 (m, 1H, H-5), 3.92 (bd, 1H, J = 2.7 Hz, H-4), 3.85 (dd, 1H, J = 3.4, 10.2 Hz, H-2), 3.80 (dd, 1H, J = 3.0,

10.2 Hz, H-3), 3.73 (dd, 1H, *J* = 7.0, 11.6 Hz, H-6a), 3.68 (dd, 1H, *J* = 5.2, 11.5 Hz, H-6b). ^{13}C NMR (100 MHz, CD_3OD, 295 K): δ 101.1 (C-1), 91.0 (CCl_3) 80.6 (*C*H_2CCl_3), 73.5 (C-3), 71.2 (C-5), 71.0 (C-4), 70.0 (C-2), 62.7 (C-6). HRMS–ESI Calcd for $C_8H_{13}Cl_3NaO_6$ $[M + Na]^+$ 332.9675, found 332.9688.

2,2,2-Trichloroethyl 2,3,6-tri-*O*-pivaloyl-α-D-galactopyranoside (15). The anomeric mixture of galactoside **14** (7.0 g, 22.7 mmol) was dissolved in a 1:1 mixture of CH_2Cl_2 and pyridine (60 mL) and the solution was cooled to –10 °C. Pivaloyl chloride (8.66 mL, 3.1 equiv) was added dropwise over 10 min and the reaction was allowed to slowly warm up to rt and stirred for an additional 12 h. Water (150 mL) was added and the organic layer was separated and co-concentrated with toluene (100 mL × 3). The residual oil was diluted with CH_2Cl_2 (200 mL) and washed with brine (200 mL), and the aq layer was re-extracted with CH_2Cl_2 (150 mL). The combined organic layers were dried, concentrated, and flash chromatography (EtOAc/hexanes, 2:8) gave the α anomer **15** pure as a colourless glass (8.2 g, 14.6 mmol, 64%). $[\alpha]_D$ = +66 (*c* 3.1, CH_2Cl_2). ^1H NMR (400 MHz, $CDCl_3$, 296 K): δ 5.39 (d, 1H, *J* = 3.8 Hz, H-1), 5.33 (dd, 1H, *J* = 3.1, 10.7 Hz, H-3), 5.21 (dd, 1H, *J* = 3.8, 10.7 Hz, H-2), 4.33–4.24 (m, 2H, H-6ab), 4.19 (d, 1H, *J* = 11.4 Hz, C*H*$HCCl_3$), 4.16 (m, 1H, H-5), 4.13 (d, 1H, *J* = 3.1 Hz, H-4), 4.07 (d, 1H, *J* = 11.4 Hz, C*H*$HCCl_3$), 1.19, 1.16 (3s, 27H, 3 x $C(CH_3)_3$). ^{13}C NMR (100 MHz, $CDCl_3$, 296 K) : δ 178.3, 178.0, 177.3 (C=O), 96.2 (C-1), 95.9 (CCl_3), 78.6 (*C*H_2CCl_3), 69.6 (C-3), 68.8 (C-5), 67.8 (C-4), 67.6 (C-2), 63.2 (C-6), 38.9, 38.8, 38.8 (*C*$(CH_3)_3$), 27.2, 27.1, 26.9 (C(*C*$H_3)_3$). HRMS–ESI Calcd for $C_{23}H_{41}Cl_3NO_9$ $[M + NH_4]^+$ 580.1847, found 580.1848.

2,2,2-Trichloroethyl 4-*O*-methyl-2,3,6-tri-*O*-pivaloyl-α-D-galactopyranoside (16). Galactopyranoside **15** (8.20 g, 14.5 mmol) was dissolved in DMF (100 mL) and MeI (45 mL, 50 equiv). Sodium hydride (700 mg, 60% dispersion in mineral oil, 1.2 equiv) was added and the

reaction was left at rt for 1 h. The reaction mixture was diluted with water (250 mL) and the aq layer was extracted with CH_2Cl_2 (75 mL × 3). The combined organic layers were washed with brine (250 mL); the brine layer was re-extracted with with CH_2Cl_2 (75 mL × 3) and the combined organic layers were dried and concentrated. Flash chromatography (EtOAc/hexanes, 2:8) gave 4-methoxy galactoside **16** (6.02 g, 10.4 mmol, 72%) as colourless glass. $[\alpha]_D$ = +74 (*c* 2.4, CH_2Cl_2). ^{1}H NMR (400 MHz, $CDCl_3$, 296 K): δ 5.36 (d, 1H, *J* = 3.7 Hz, H-1), 5.32 (dd, 1H, *J* = 3.0, 10.8 Hz, H-3), 5.18 (dd, 1H, *J* = 3.7, 10.8 Hz, H-2), 4.21–4.17 (m, 2H, H-6ab), 4.16 (d, 1H, *J* = 11.4 Hz, C*H*HCCl$_3$), 4.11 (m, 1H, H-5), 4.03 (d, 1H, *J* = 11.4 Hz, C*H*HCCl$_3$), 3.74 (bd, 1H, *J* = 3.0 Hz, H-4), 3.46 (s, 3H, OCH_3), 1.19, 1.17, 1.42 (3s, 27H, 3 x $C(CH_3)_3$). ^{13}C NMR (100 MHz, $CDCl_3$, 296 K): δ 178.0, 177.8, 177.6 (C=O), 96.2 (C-1), 95.9 (CCl_3), 78.5 (*C*H$_2$CCl$_3$), 77.4 (C-4), 70.3 (C-3), 69.2 (C-5), 68.1 (C-2), 62.8 (C-6), 61.5 (OCH_3), 38.9, 38.8, 38.7 (*C*(CH$_3$)$_3$), 27.2 (C(*C*H$_3$)$_3$). HRMS–ESI Calcd for $C_{24}H_{43}Cl_3NO_9$ $[M + NH_4]^+$ 594.2003, found 594.2010.

2,2,2-Trichloroethyl 2,3,6-tri-*O*-pivaloyl-α-D-glucopyranoside (17). Galactopyranoside **15** (15.2 g, 27 mmol) was dissolved in CH_2Cl_2 (135 mL) and pyridine (15.0 mL, 7.0 equiv). The mixture was cooled to −20 °C and Tf_2O (9.08 mL, 2 equiv) was added dropwise over 10 min. The reaction was allowed to warm to rt, stirred for 30 min, diluted with CH_2Cl_2 (300 mL), and washed with 2 M HCl (300 mL) and saturated aq $NaHCO_3$ (300 mL). The aq layers were re-extracted with CH_2Cl_2 (100 mL × 3) and the combined organic phases were dried and concentrated. The crude triflate was dissolved in DMF (500 mL), $NaNO_2$ (18.6 g, 10.0 equiv) was added and the reaction was stirred at 50 °C for 12 h. The solvent was evaporated and the residue was dissolved in CH_2Cl_2 (300 mL) and washed with brine (300 mL). The aq layer was re-extracted with CH_2Cl_2 (100 mL × 3), the combined organic layers were dried and

concentrated, and flash chromatography (EtOAc/hexanes, 2:8) gave glucopyranoside **17** (10.9 g, 19.4 mmol, 72%) pure as a white foam. $[\alpha]_D$ = +58 (*c* 1, CH_2Cl_2). ^{1}H NMR (400 MHz, $CDCl_3$, 295 K): δ 5.38 (t, 1H, *J* = 9.7 Hz, H-3), 5.33 (d, 1H, *J* = 3.8 Hz, H-1), 4.83 (dd, 1H, *J* = 3.8, 10.1 Hz, H-2), 4.40–4.32 (m, 2H, H-6ab), 4.20, 4.07 (2d AB_{system}, 2H, *J* = 11.5 Hz, CH_2CCl_3), 3.95 (m, 1H, H-5), 3.50 (bt, 1H, *J* = 9.6 Hz, H-4), 3.04 (bs, 1H, OH), 1.22, 1.19, 1.17 (3s, 27H, 3 x $C(CH_3)_3$). ^{13}C NMR (100 MHz, $CDCl_3$, 295 K): δ 177.3 (C=O), 95.4 (C-1), 95.4 (CCl_3), 78.1 ($\mathit{C}H_2CCl_3$), 72.2 (C-3), 70.6 (C-5), 69.6 (C-4), 69.4 (C-2), 62.2 (C-6), 38.4, 38.3 ($\mathit{C}(CH_3)_3$) 26.7, 26.6 ($C(\mathit{C}H_3)_3$). HRMS–ESI Calcd for $C_{23}H_{41}Cl_3NO_9$ $[M + NH_4]^+$ 563.1603, found 563.1581.

2,2,2-Trichloroethyl 4-chloro-4-deoxy-2,3,6-tri-*O*-pivaloyl-α-D-galactopyranoside (18). Glucopyranoside **17** (6.74 g, 12 mmol) was dissolved in a 2:1 mixture of $CHCl_3$ and pyridine (120 mL), and the mixture was cooled to −40 °C. Sulfuryl chloride (2.91 mL) was added dropwise over 10 min, and the reaction mixture was allowed to slowly warm up to rt and stirred for 12 h. The mixture was co-concentrated with toluene (100 mL × 3), and the crude residue was diluted in CH_2Cl_2 (300 mL), and washed with 2 M aq HCl (300 mL) and aq saturated $NaHCO_3$ (300 mL). The aq layers were re-extracted with CH_2Cl_2 (100 mL × 3), the combined organic layers were dried and concentrated, and flash chromatography (EtOAc/hexanes, 1:9) of the residue gave the 4-chloro galactoside **18** (5.23 g, 9.36 mmol, 78%) pure as a white foam. $[\alpha]_D$ = +90 (*c* 3.3, CH_2Cl_2). ^{1}H NMR (400 MHz, $CDCl_3$, 295 K): δ 5.42 (d, 1H, *J* = 3.8 Hz, H-1), 5.35 (dd, 1H, *J* = 3.7, 10.6 Hz, H-3), 5.22 (dd, 1H, *J* = 3.8, 10.6 Hz, H-2), 4.59 (dd, 1H, *J* = 1.0, 3.7 Hz, H-4), 4.41 (m, 1H, H-5), 4.27 (dd, 1H, *J* = 7.5, 11.6 Hz, H-6a), 4.20 (d, 1H, *J* = 11.3 Hz, $C\mathit{H}HCCl_3$), 4.17 (dd, 1H, *J* = 4.2, 11.7 Hz, H-6b), 4.06 (d, 1H, *J* = 11.4 Hz, $C\mathit{H}HCCl_3$), 1.19, 1.17 (3s, 27H, 3 x $C(CH)_3$). ^{13}C NMR (100 MHz, $CDCl_3$, 295 K): δ 177.9, 177.7, 177.6 (C=O), 96.1 (C-1), 95.7 (CCl_3), 78.6 ($\mathit{C}H_2CCl_3$), 68.2 (C-3), 67.7 (C-5), 67.2 (C-2), 63.9 (C-6), 58.5 (C-

4), 38.9, 38.8, 38.7 ($C(CH_3)_3$), 27.1, 27.1, 27.0 ($C(CH_3)_3$). HRMS–ESI Calcd for $C_{23}H_{40}Cl_4NO_8$ $[M + NH_4]^+$ 598.1508, found 598.1498.

2,2,2-Trichloroethyl 4-deoxy-4-fluoro-2,3,6-tri-*O*-pivaloyl-α-D-galactopyranoside (19). Glucopyranoside **17** (3.98 g, 7.06 mmol) was dissolved in CH_2Cl_2 (40 mL) and pyridine (8.0 mL, 14.0 equiv), the mixture was cooled to −20 °C, and Tf_2O (9.08 mL, 2 equiv) was added dropwise over 10 min. The reaction mixture was allowed to slowly warm to rt over 1 h, stirred for a further 30 min, diluted with CH_2Cl_2 (70 mL), and washed with 2 M aq HCl (70 mL) and saturated aq $NaHCO_3$ (70 mL). The aq layers were re-extracted with CH_2Cl_2 (30 mL × 3) and the combined organic layers were dried and concentrated. The crude triflate was dissolved in THF (140 mL), a solution of TBAF in THF (1.0 M, 10.2 mL, 1.4 equiv) was added, and the mixture was stirred for 1 h at rt. Solvents were evaporated, the residue was dissolved in CH_2Cl_2 (75 mL), and washed with aq saturated $NaHCO_3$ (75 mL) and 2 M aq HCl (75 mL). The aq layers were re-extracted with CH_2Cl_2 (30 mL × 3), the combined organic layers were dried and concentrated, and flash chromatography (EtOAc/hexanes, 1:9) of the residue gave the 4-fluoro galactoside **19** (3.31 g, 5.85 mmol, 83%) pure as a white foam. $[\alpha]_D$ = +135 (*c* 0.3, CH_2Cl_2). ^{1}H NMR (400 MHz, $CDCl_3$, 295 K): δ 5.42 (d, 1H, J = 3.6 Hz, H-1), 5.30 (ddd, 1H, J = 2.6, 10.8 Hz, $J_{H,F}$ = 26.4 Hz, H-3), 5.18 (dd, 1H, J = 3.6, 10.3 Hz, H-2), 4.90 (dd, 1H, J = 2.6 Hz, $J_{H,F}$ = 50.8 Hz, H-4), 4.24 (m, 2H, H6ab), 4.21 (m, 1H, H-5), 4.18 (d, 1H, J = 11.4 Hz, $CHHCCl_3$), 4.07 (d, 1H, J = 11.4 Hz, $CHHCCl_3$), 1.18, 1.16 (2s, 27H, 3 x $C(CH_3)_3$). ^{13}C NMR (100 MHz, $CDCl_3$, 295 K): δ 177.9, 177.7 (C=O), 96.2 (C-1), 86.6 (d, $J_{C,F}$ = 186.4 Hz, C-4), 78.7 (CCl_3), 67.9 (2d, $J_{C,F}$ = 17.4 Hz, $J_{C,F}$ = 18.2 Hz, C-3, C-5), 67.3 (CH_2CCl_3, C-2), 62.1 (C-6), 38.9, 38.8 ($C(CH_3)_3$), 27.1, 27.0, 26.9 ($C(CH_3)_3$). HRMS–ESI Calcd for $C_{23}H_{40}Cl_3FNO_8$ $[M + NH_4]^+$ 582.1804, found 582.1817.

Methyl 2-acetamido-6-*O*-benzyl-2-deoxy-4-*O*-(4-*O*-methyl-2,3,6-tri-*O*-pivaloyl-β-D-galactopyranosyl)-β-D-glucopyranoside (23). Thiourea (151 mg, 10.0 equiv) was added to a solution of disaccharide **20** (165 mg, 0.199 mmol) in pyridine/EtOH (1:1, 5.0 mL), and the mixture was stirred at 80 °C for 2 h. Solvents were evaporated, and the residue was co-concentrated with toluene (3 × 10 mL), dissolved in CH_2Cl_2 (40 mL), and washed with 2 M aq HCl (30 mL) and saturated aq $NaHCO_3$ (30 mL). The aq layers were re-extracted with CH_2Cl_2 (3 × 10 mL), and the combined organic layers were dried, and concentrated. Flash chromatography (EtOAc/hexanes, 8:2) of the residue gave disaccharide acceptor **23** (127 mg, 0.169 mmol, 85%) pure as a colourless glass. $[\alpha]_D = -8$ (*c* 2.6, CH_2Cl_2). ^{1}H NMR (400 MHz, $CDCl_3$, 296 K): δ 7.30 (m, 5H, H_{arom}), 5.80 (d, 1H, *J* = 7.9 Hz, NH), 5.24 (dd, 1H, *J* = 8.0, 10.4 Hz, H-2’), 4.77 (dd, 1H, *J* = 3.0, 10.4 Hz, H-3’), 4.69 (d, 1H, *J* = 12.1 Hz, C*H*HPh), 4.61 (d, 1H, *J* = 7.3 Hz, H-1), 4.42 (d, 1H, *J* = 12.1 Hz, C*H*HPh), 4.26 (d, 1H, *J* = 8.0 Hz, H-1’), 4.19 (m, 2H, H-6ab’), 3.89 (m, 1H, H-3), 3.68–3.43 (m, 7H, H-2, H-4, H-5, H-6ab, H-4’, H-5’), 3.44, 3.43 (2s, 6H, 2 x OCH_3), 1.98 (s, 3H, $C(O)CH_3$), 1.18, 1.17, 1.12 (3s, 27H, 3 x $C(CH_3)_3$). ^{13}C NMR (100 MHz, $CDCl_3$ 296 K): δ 178.0, 177.7, 176.5, 170.6 (C=O), 138.0, 128.5, 128.0, 127.9 (Ar), 101.1 (C-1), 99.8 (C-1’), 77.6 (C-4), 76.4 (C-4’), 74.0 (C-5), 73.5 (CH_2Ph), 73.3 (C-3’), 72.6 (C-5’), 70.8 (C-3), 69.1 (C-2’), 68.3 (C-6), 62.0 (C-6’), 61.6, 56.7 (OCH_3), 55.7 (C-2), 38.9, 38.7 ($C(CH_3)_3$), 27.2, 27.1, 27.1 ($C(CH_3)_3$), 23.5 ($C(O)CH_3$). HRMS–ESI Calcd for $C_{38}H_{59}NNaO_{14}$ $[M + Na]^+$ 776.3833, found 776.3853.

Methyl 2-acetamido-6-*O*-benzoyl-4-*O*-(4-chloro-4-deoxy-2,3,6-tri-*O*-pivaloyl-β-D-galactopyranosyl)-2-deoxy-β-D-glucopyranoside (24). Removal of the chloroacetate in disaccharide **21** (103 mg, 0.121 mmol) using thiourea (92 mg, 10.0 equiv) was accomplished as described above for the preparation of disaccharide acceptor **23**. Work up of the reaction was

carried out as described above, and flash chromatography (EtOAc/hexanes, 6:4) of the residue gave disaccharide acceptor **24** (65 mg, 0.0842 mmol, 70%) pure as a colourless glass. $[\alpha]_D$ = +21 (*c* 0.5, CH_2Cl_2). ^{1}H NMR (400 MHz, $CDCl_3$, 300 K): δ 8.02–7.35 (d, 5H, H_{arom}), 5.73 (bd, 1H, *J* = 7.4 Hz, NH), 5.36 (dd, 1H, *J* = 8.1, 10.0 Hz, H-2'), 4.92 (dd, 1H, *J* = 3.9, 10.2 Hz, H-3'), 4.68 (d, 1H, *J* = 7.4 Hz, H-1), 4.65 (d, 1H, *J* = 8.0 Hz, H-1'), 4.53 (dd, 1H, *J* = 2.6, 11.7 Hz, H-6a), 4.46–4.43 (m, 2H, H-6b, H-4'), 4.28 (dd, 1H, *J* = 2.6, 11.7 Hz, H-6a'), 4.21 (dd, 1H, *J* = 4.7, 11.7 Hz, H-6b'), 4.04 (m, 1H, H-3), 3.98 (m, 1H, H-5'), 3.95 (d, 1H, *J* = 2.2 Hz, OH), 3.77 (m, 1H, H-5), 3.62 (t, 1H, *J* = 8.3 Hz, H-4), 3.52 (bq, 1H, *J* = 7.8 Hz, H-2), 3.44 (s, 3H, OCH_3), 2.01 (s, 3H, $C(O)CH_3$), 1.17, 1.16, 1.14 (3s, 27H, 3 x $C(CH_3)_3$). ^{13}C NMR (100 MHz, $CDCl_3$, 300 K): δ 177.9, 177.6, 170.7 (C=O), 133.3, 129.7, 129.6, 128.5 (Ar), 101.0 (C-1), 100.8 (C-1'), 79.6 (C-4), 72.2 (C-5), 71.7 (C-5'), 71.6 (C-3'), 71.1 (C-3), 68.2 (C-2'), 63.4 (C-6), 63.2 (C-6'), 57.1 (C-4'), 56.7 (OCH_3), 56.0 (C-2), 38.9, 38.8, 38.7 ($C(CH_3)$), 27.1, 27.0, 27.0 ($C(CH_3)_3$), 23.6 ($C(O)CH_3$). HRMS–ESI Calcd for $C_{37}H_{55}ClNO_{14}$ $[M + H]^+$ 772.3311, found 772.3308.

Methyl 2-acetamido-6-*O*-benzoyl-2-deoxy-4-*O*-(4-deoxy-4-fluoro-2,3,6-tri-*O*-pivaloyl-β-D-galactopyranosyl)-β-D-glucopyranoside (25). Removal of the chloroacetate in disaccharide **22** (143 mg, 0.172 mmol) by using thiourea (131 mg, 10.0 equiv) was accomplished as described above for the preparation of disaccharide acceptor **23**. Work up of the reaction was carried out as described above, and flash chromatography (EtOAc/hexanes, 7:3) of the residue gave disaccharide acceptor **25** (92 mg, 0.122 mmol, 71%) pure as a colourless glass. $[\alpha]_D$ = +20 (*c* 0.6, CH_2Cl_2). ^{1}H NMR (400 MHz, $CDCl_3$, 295 K): δ 8.01–7.20 (d, 5H, H_{arom}), 5.73 (d, 1H, *J* = 7.8 Hz, NH), 5.34 (t, 1H, *J* = 8.2 Hz, H-2'), 4.88 (ddd, 1H, *J* = 2.4, 12.4 Hz, $J_{H,F}$ = 26.5 Hz, H-3'), 4.75 (bd, 1H, $J_{H,F}$ = 50.9 Hz, H-4'), 4.69 (d, 1H, *J* = 7.5 Hz, H-1), 4.64 (d, 1H, *J* = 8.0 Hz, H-1'), 4.55 (dd, 1H, *J* = 2.4, 11.6 Hz, H-6a), 4.45 (dd, 1H, *J* = 5.4, 11.6 Hz, H-6b), 4.27 (d, 2H, *J* = 6.2

Hz, H-6ab'), 4.02 (m, 1H, H-3), 3.90 (bd, 1H, J = 2.0 Hz, OH), 3.87–3.77 (m, 2H, H-5, H-5'), 3.63 (bt, 1H, J ~ 8.0 Hz, H-4), 3.53 (bq, 1H, J ~ 8.0 Hz, H-2), 3.44 (s, 3H, OCH_3), 2.02 (s, 3H, $C(O)CH_3$), 1.18, 1.16, 1.14 (3s, 27H, 3 x $C(CH_3)_3$). ^{13}C NMR (100 MHz, $CDCl_3$, 295 K): δ 178.0, 177.7, 176.6, 170.7, 166.1 (C=O), 133.3, 129.7, 129.6, 128.5 (Ar), 101.0 (C-1), 100.3 (C-1'), 85.4 (d, $J_{C,F}$ = 186.9 Hz, C-4'), 79.4 (C-4), 72.2 (C-5), 71.6 (d, $J_{C,F}$ = 18.3 Hz, C-5'), 71.2 (d, $J_{C,F}$ = 17.9 Hz, C-3'), 71.0 (C-3), 68.4 (C-2'), 63.4 (C-6), 61.7 (C-6'), 56.7 (OCH_3), 55.9 (C-2), 38.9, 38.8, 38.7 ($\mathit{C}(CH_3)_3$), 27.6, 27.1, 27.0 ($C(\mathit{C}H_3)_3$), 23.5 ($C(O)\mathit{C}H_3$). HRMS–ESI Calcd for $C_{37}H_{55}FNO_{14}$ $[M + H]^+$ 756.3607, found 756.3623.

References

1. Hendel, J. L.; Cheng, A.; Auzanneau, F.-I. *Carbohydr. Res.* **2008,** *343,* 2914–2923. doi:10.1016/j.carres.2008.08.025
2. Risbood, P. A.; Reed, L. A., III; Goodman, L. *Carbohydr. Res.* **1981,** *88,* 245–251. doi:10.1016/S0008-6215(00)85538-X
