# Supplementary material for: Synthesis of 4” manipulated Lewis X trisaccharide analogues
Source: Beilstein J Org Chem. 2012 Jul 23;8:1134–43. doi: 10.3762/bjoc.8.126 (PMC3458731; doi:10.3762/bjoc.8.126)

Supporting Information

for

Synthesis of 4” manipulated Lewis X trisaccharide analogues

Christopher J. Moore and France-Isabelle Auzanneau*

Address: Department of Chemistry, University of Guelph, 50 Stone Rd. East, Guelph, Ontario, N1G 2W1, Canada

Email: France-Isabelle Auzanneau - fauzanne@uoguelph.ca

*Corresponding author

^{1}H NMR and ^{13}C NMR for compounds **3–5, 8–11**, **14–28**.

3, ^{1}H NMR (D_2O, 295 K, 400 MHz)

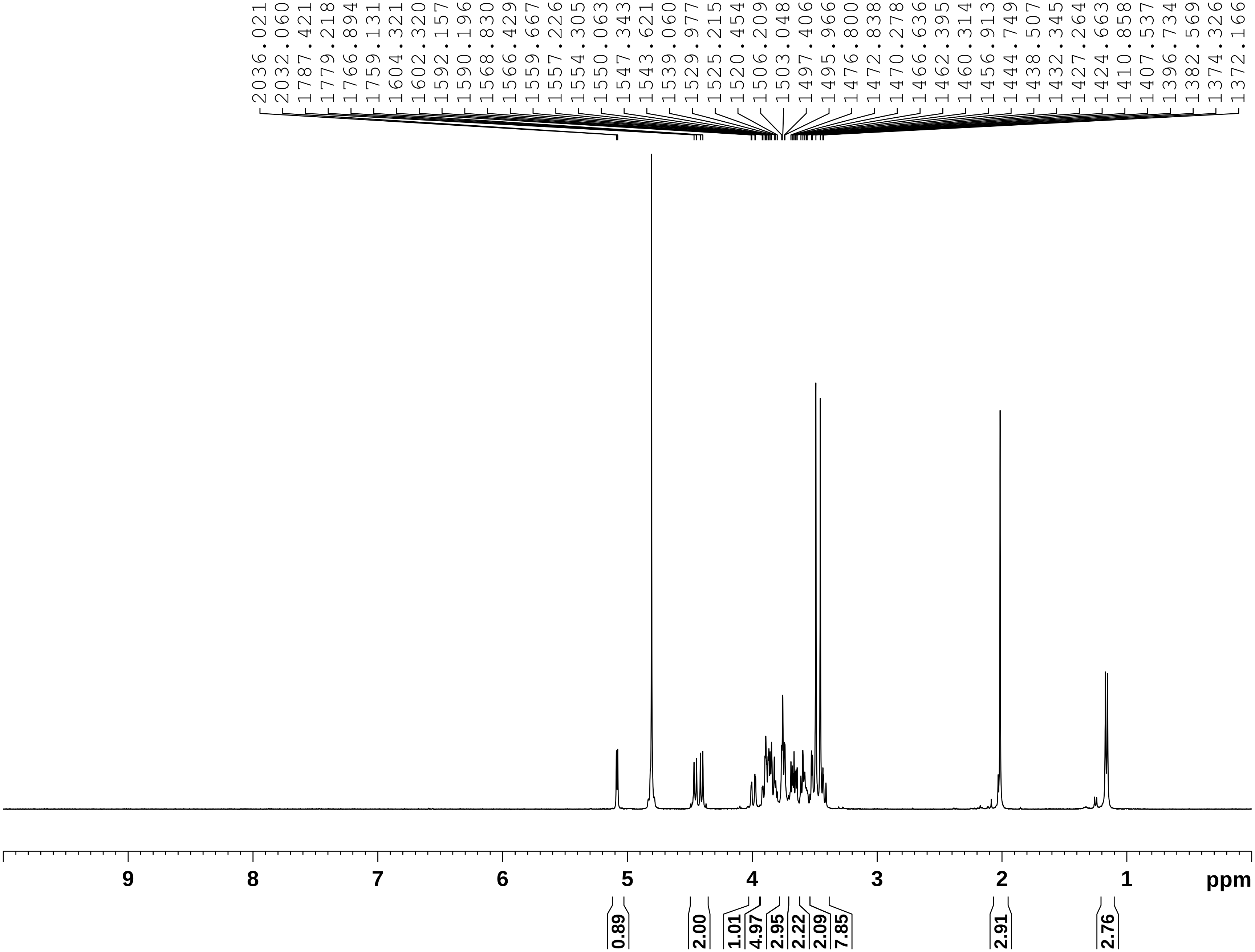

3, ^{13}C NMR (D_2O, 295 K, 100 MHz)

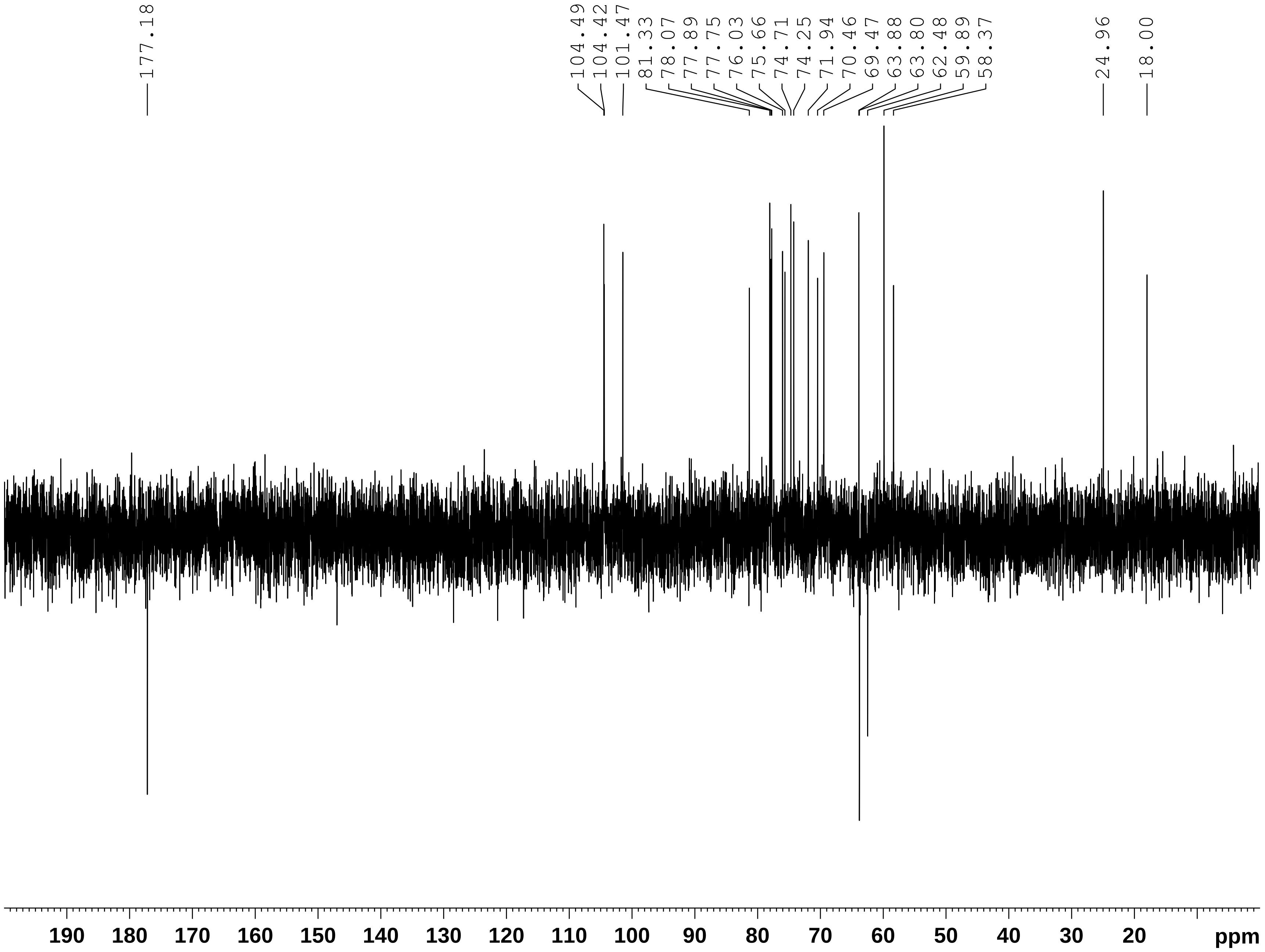

4, ^{1}H NMR (D_2O, 295 K, 400 MHz)

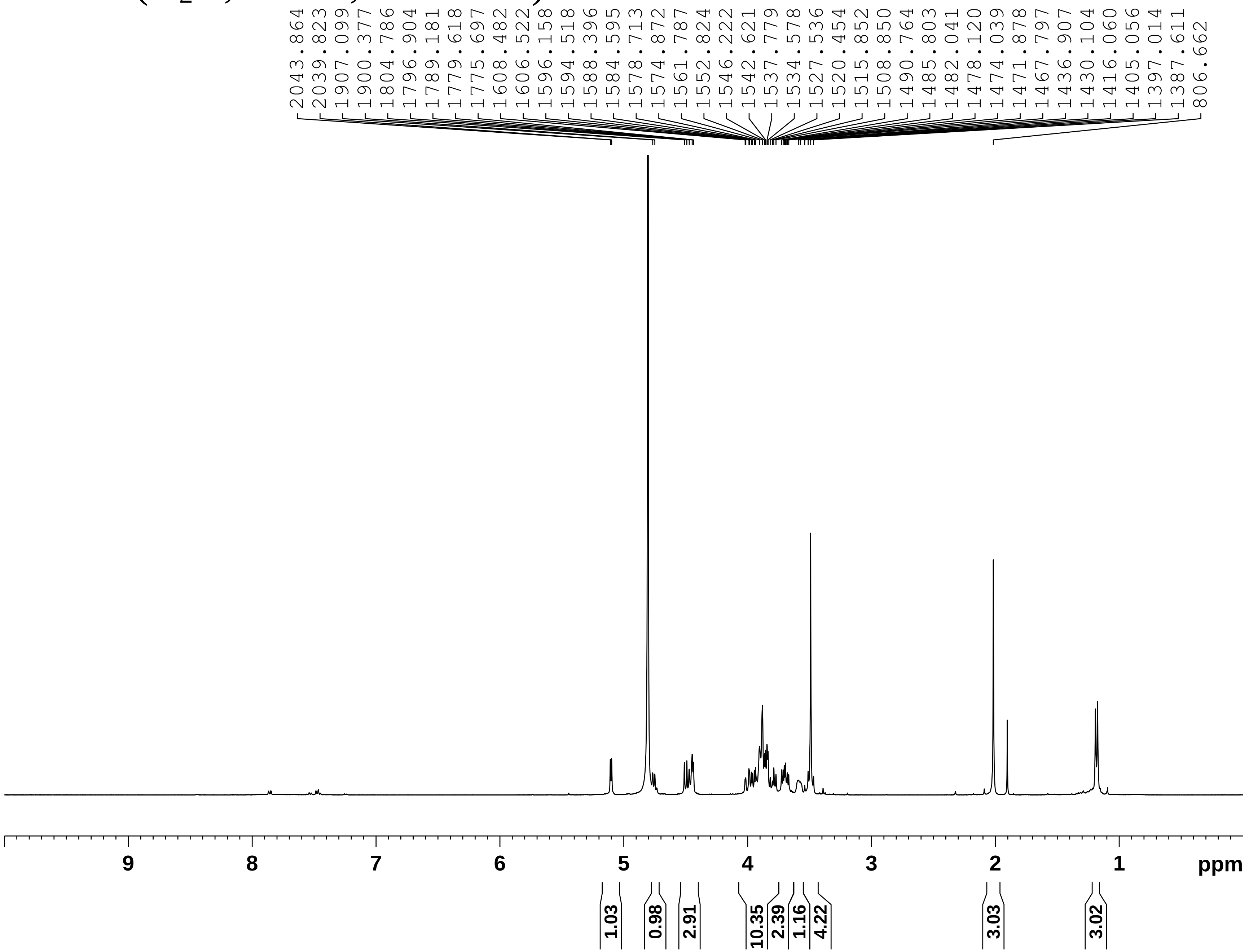

4, ^{13}C NMR (D_2O, 295 K, 100 MHz)

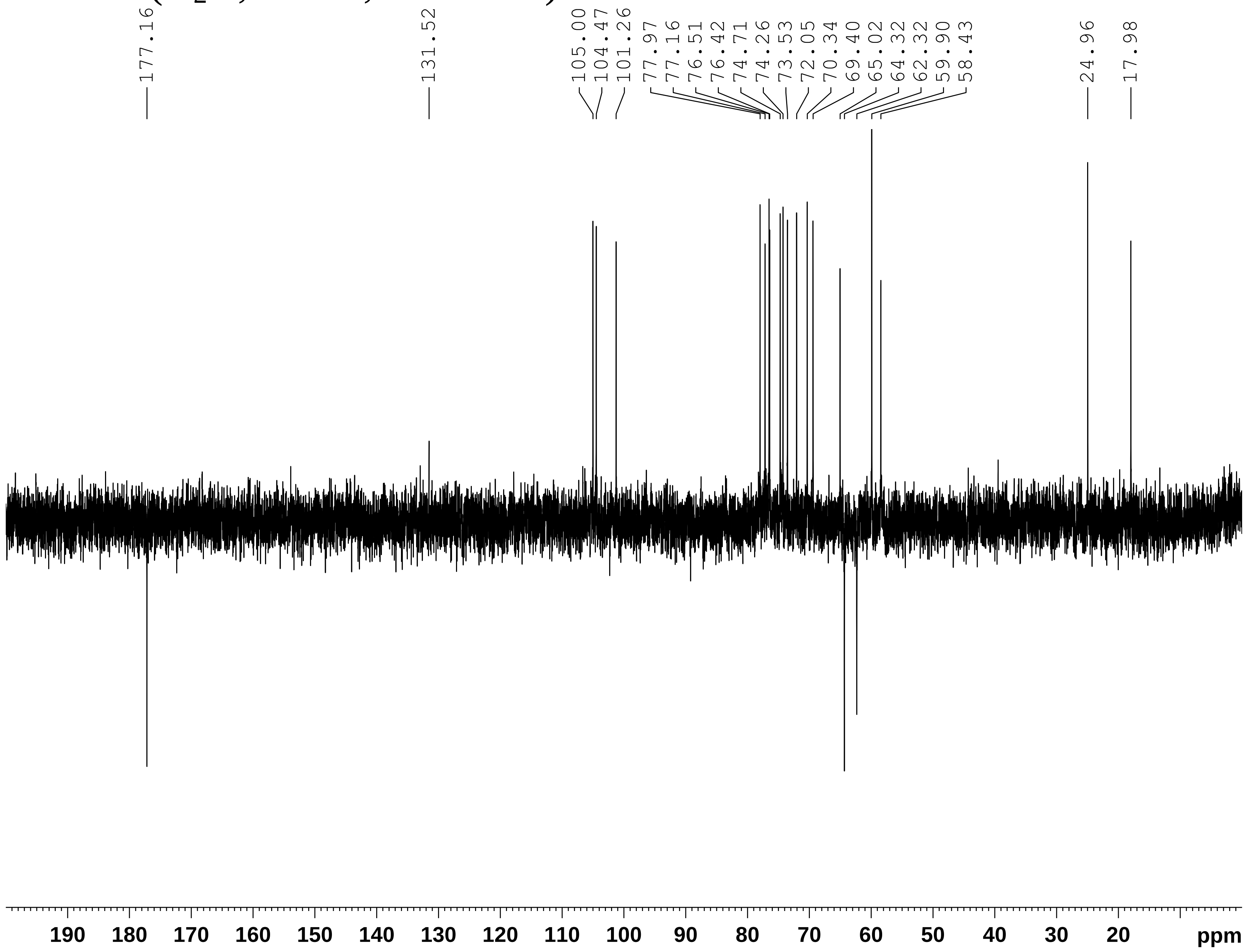

5, ^{1}H NMR (D_2O, 295 K, 400 MHz)

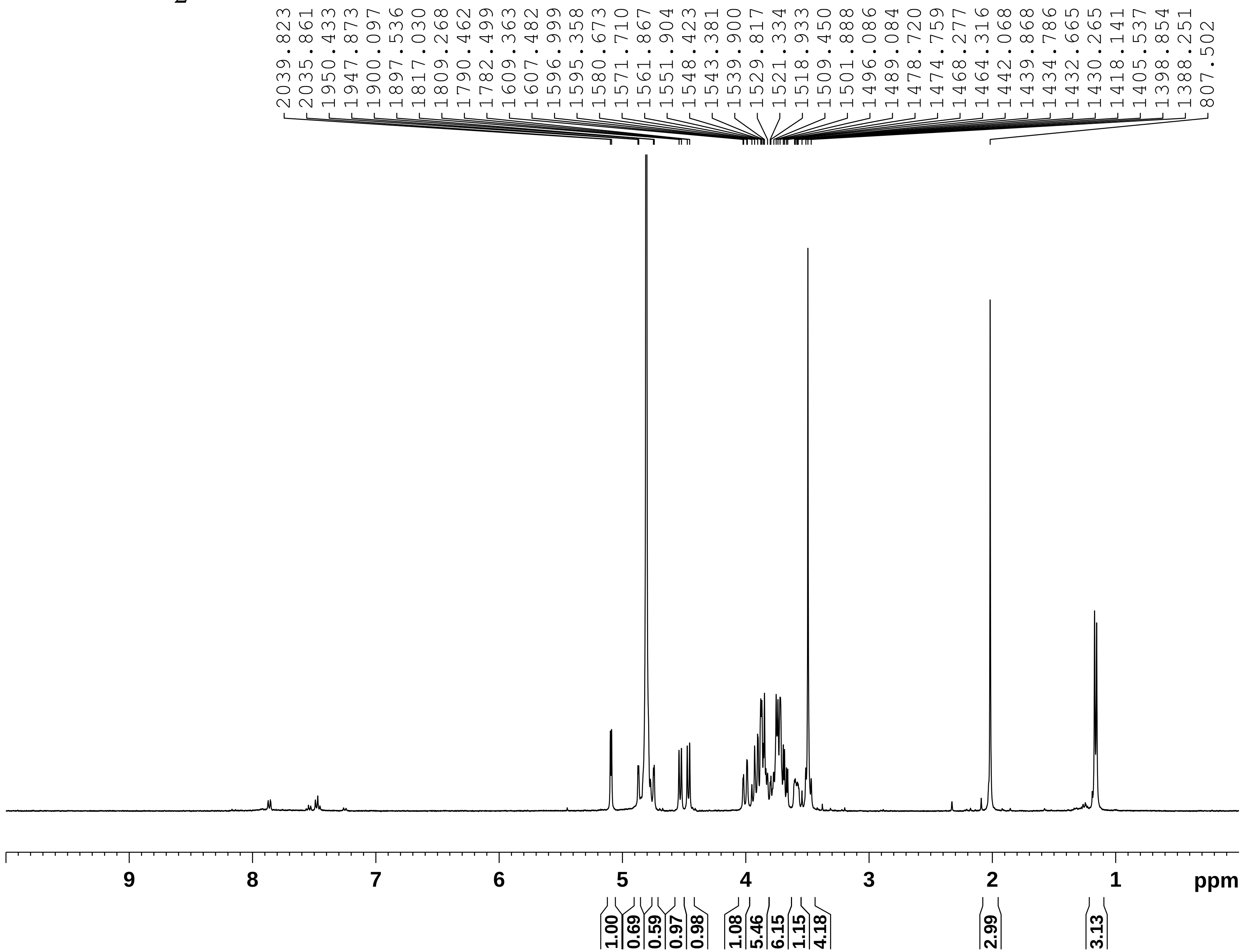

5, ^{13}C NMR (D_2O, 295 K, 100 MHz)

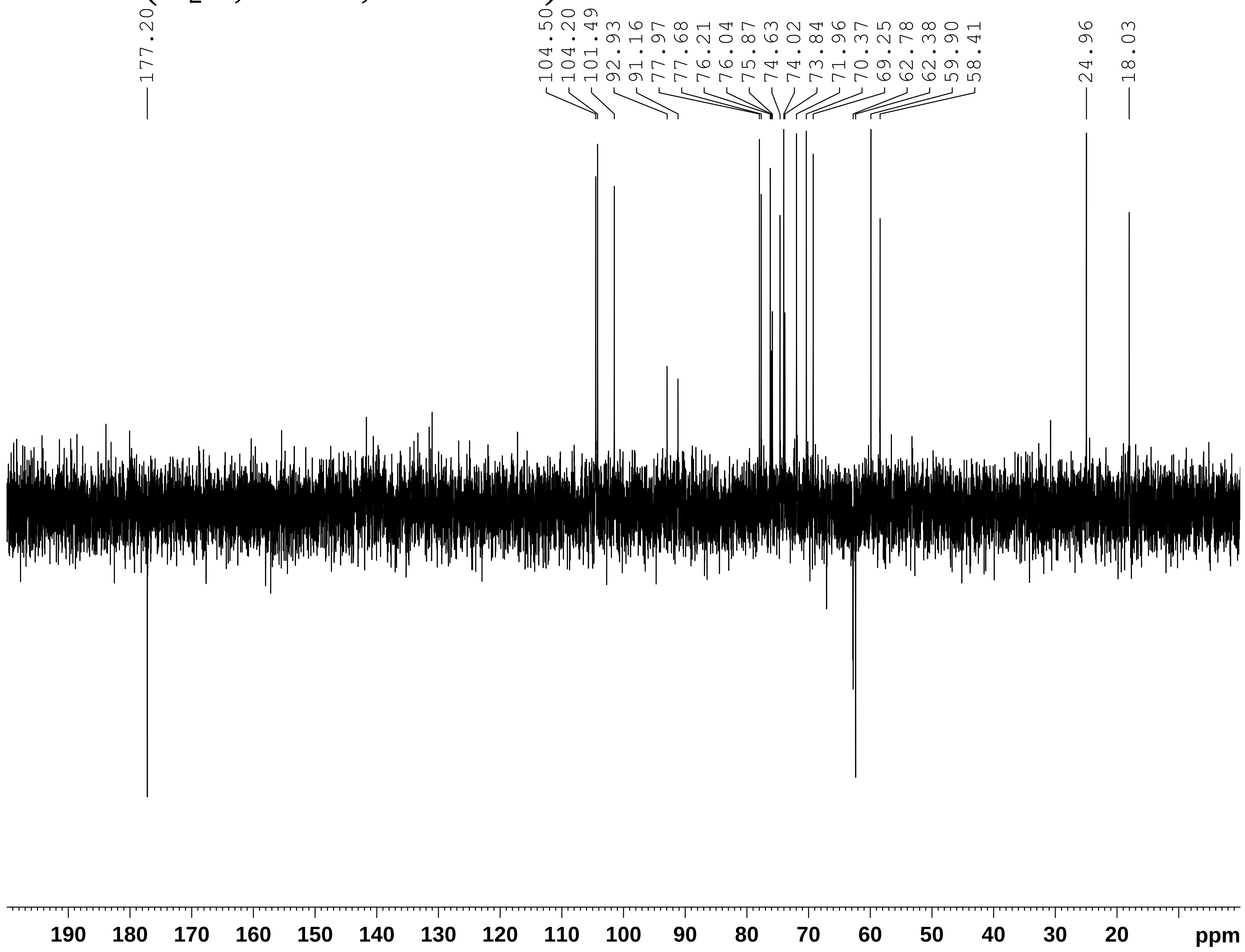

8, ^{1}H NMR (DMSO-d_6, 295 K, 400 MHz)

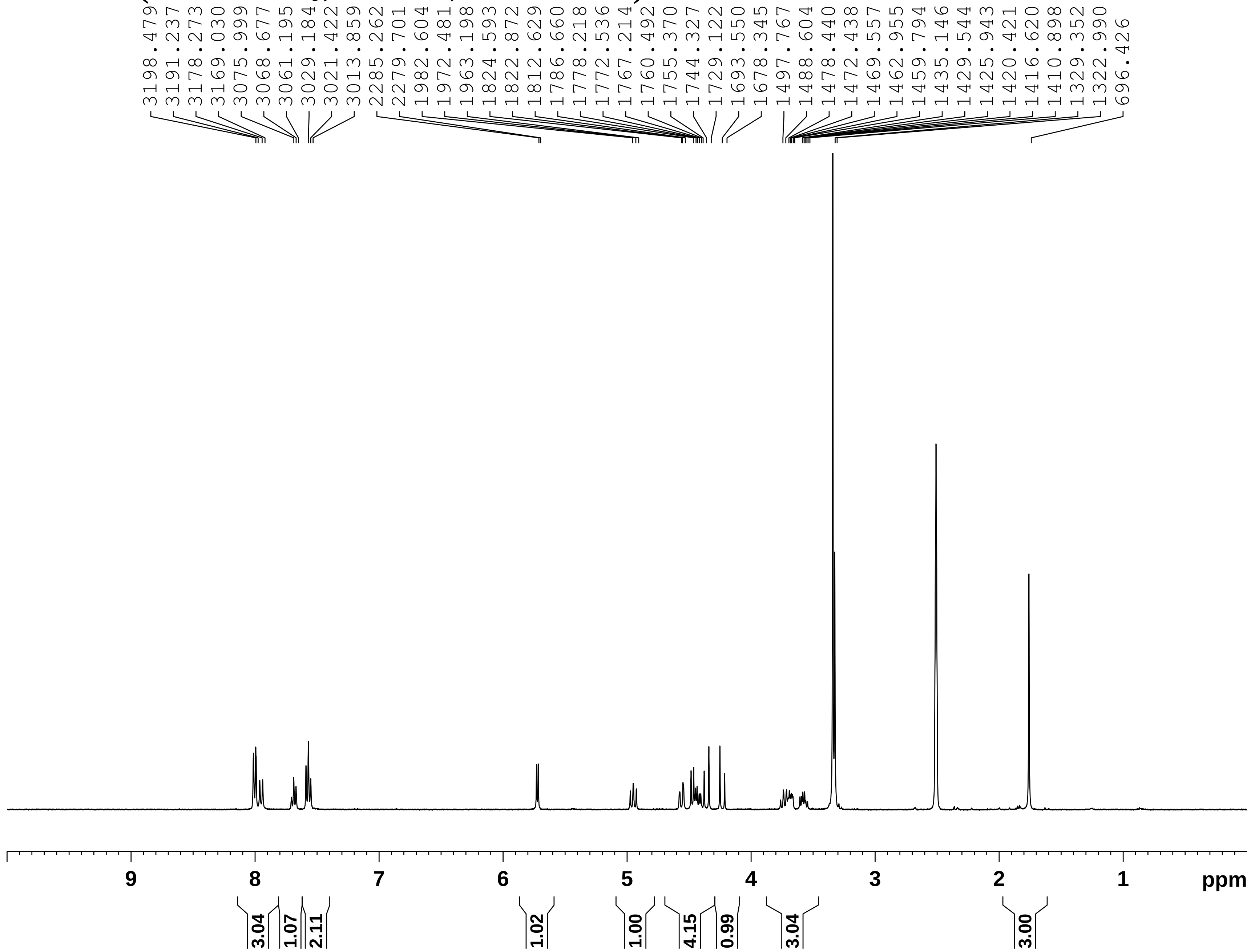

8, ^{13}C NMR (DMSO-d_6, 295 K, 100 MHz)

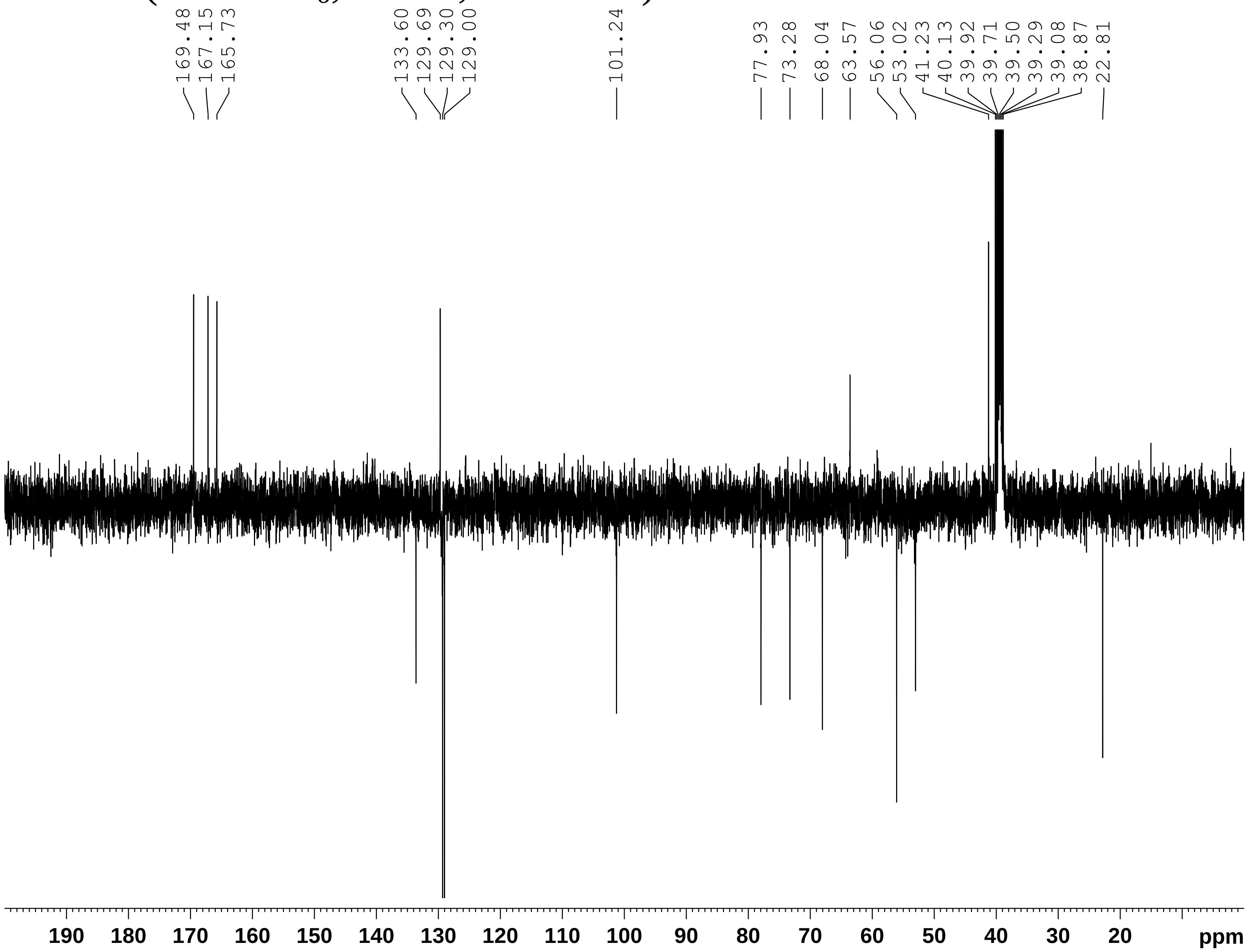

9, ^{1}H NMR ($CDCl_3$, 296 K, 400 MHz)

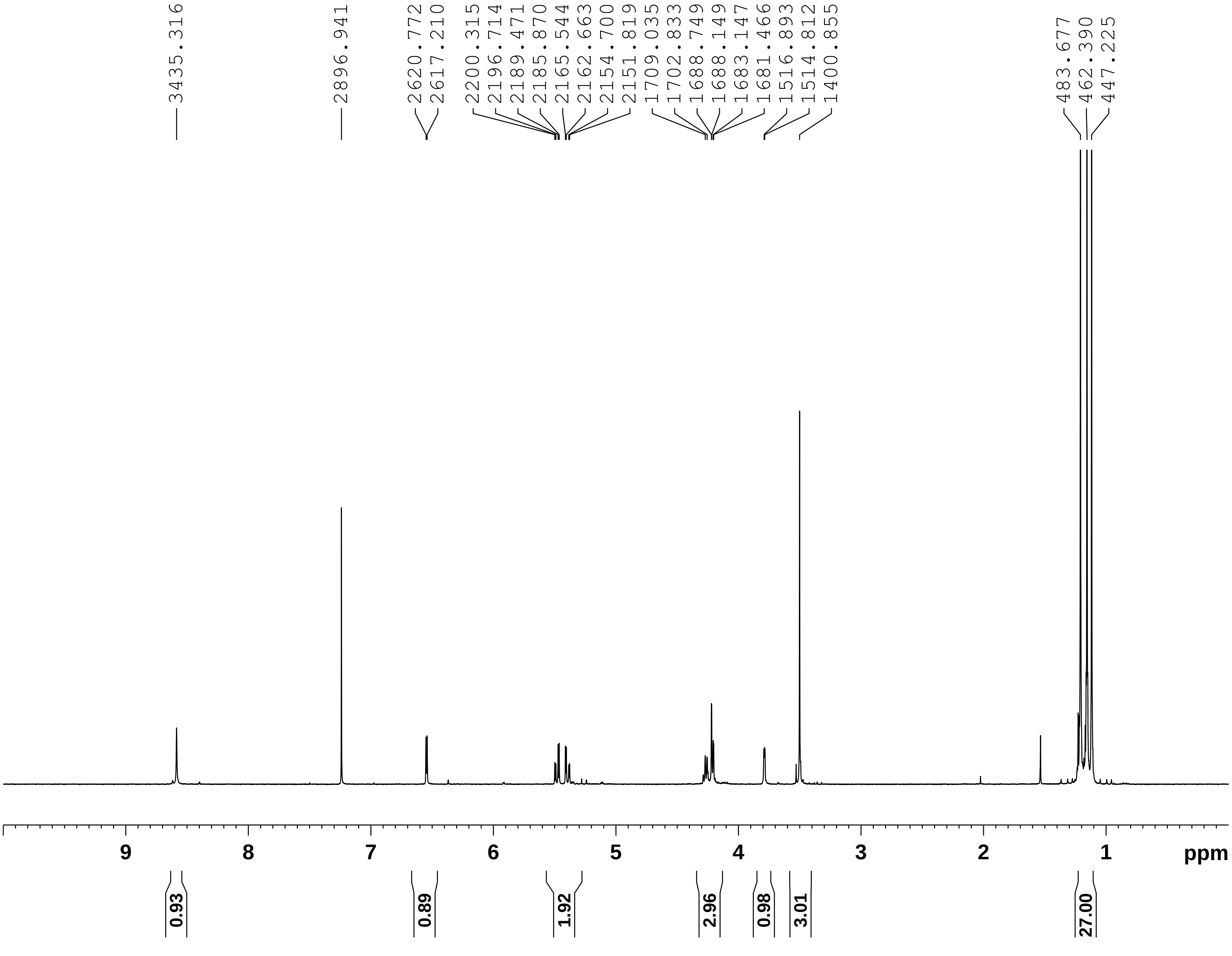

9, ^{13}C NMR ($CDCl_3$, 296 K, 100 MHz)

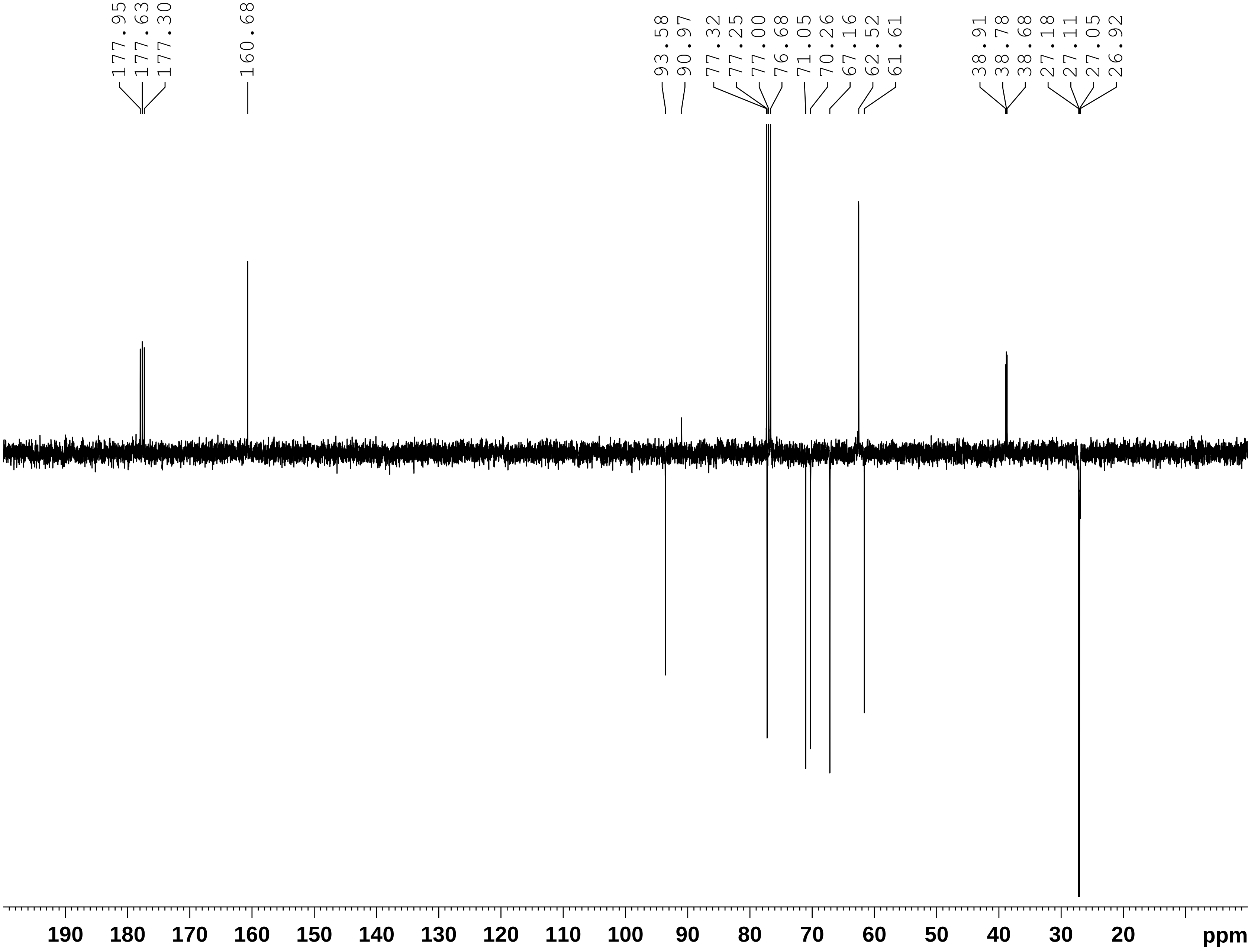

10, ^{1}H NMR ($CDCl_3$, 295 K, 400 MHz)

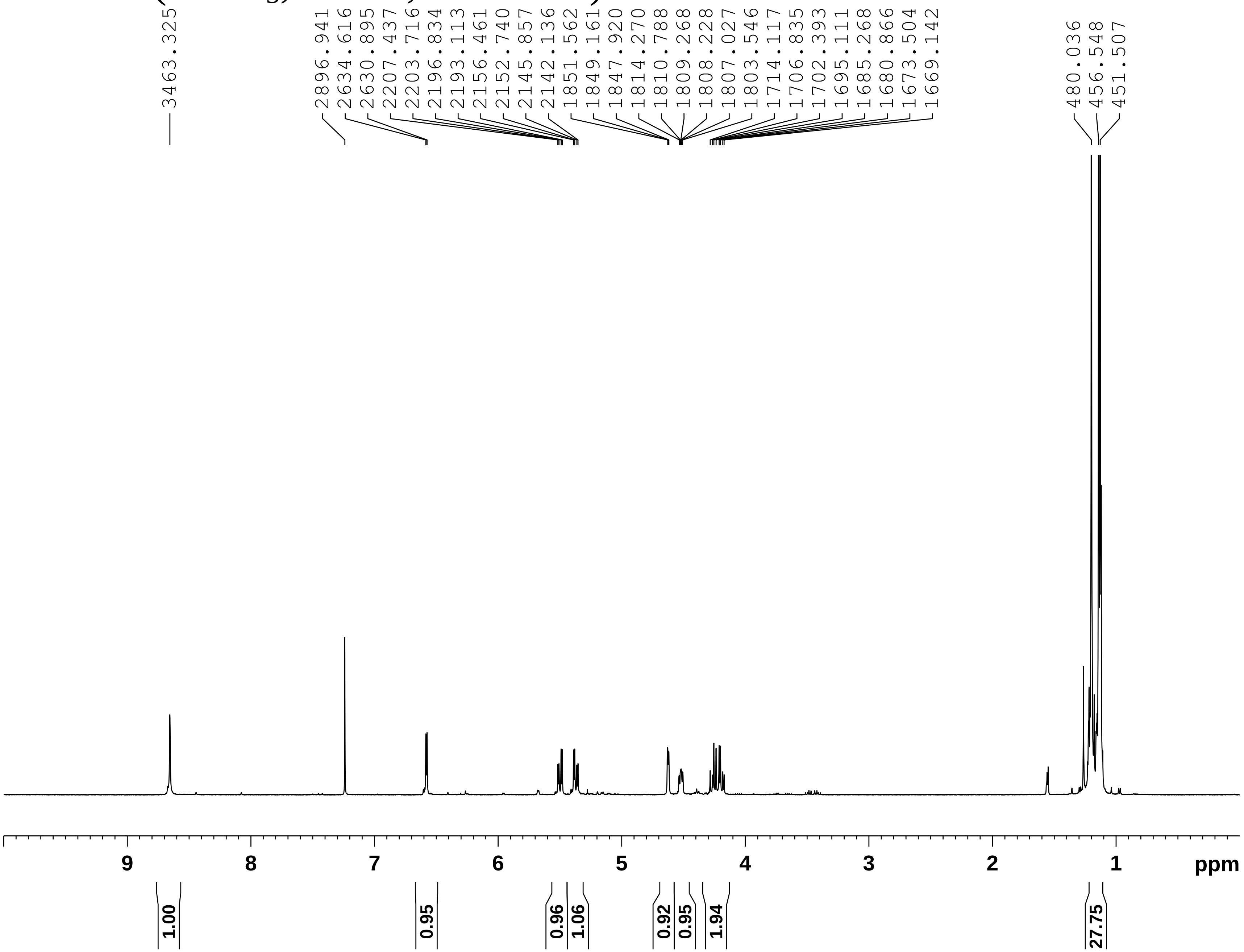

10, ^{13}C NMR ($CDCl_3$, 295 K, 100 MHz)

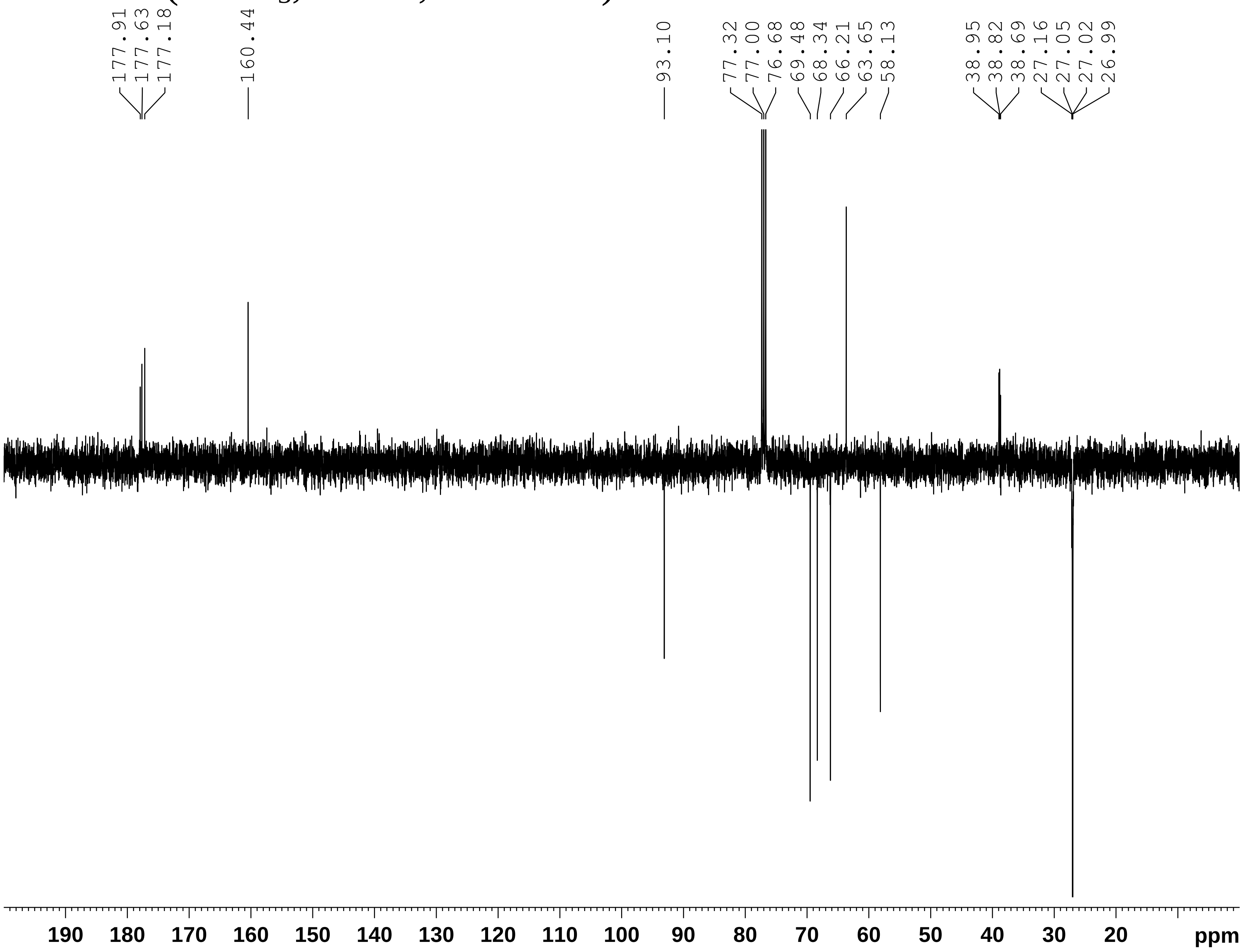

11, ^{1}H NMR ($CDCl_3$, 295 K, 400 MHz)

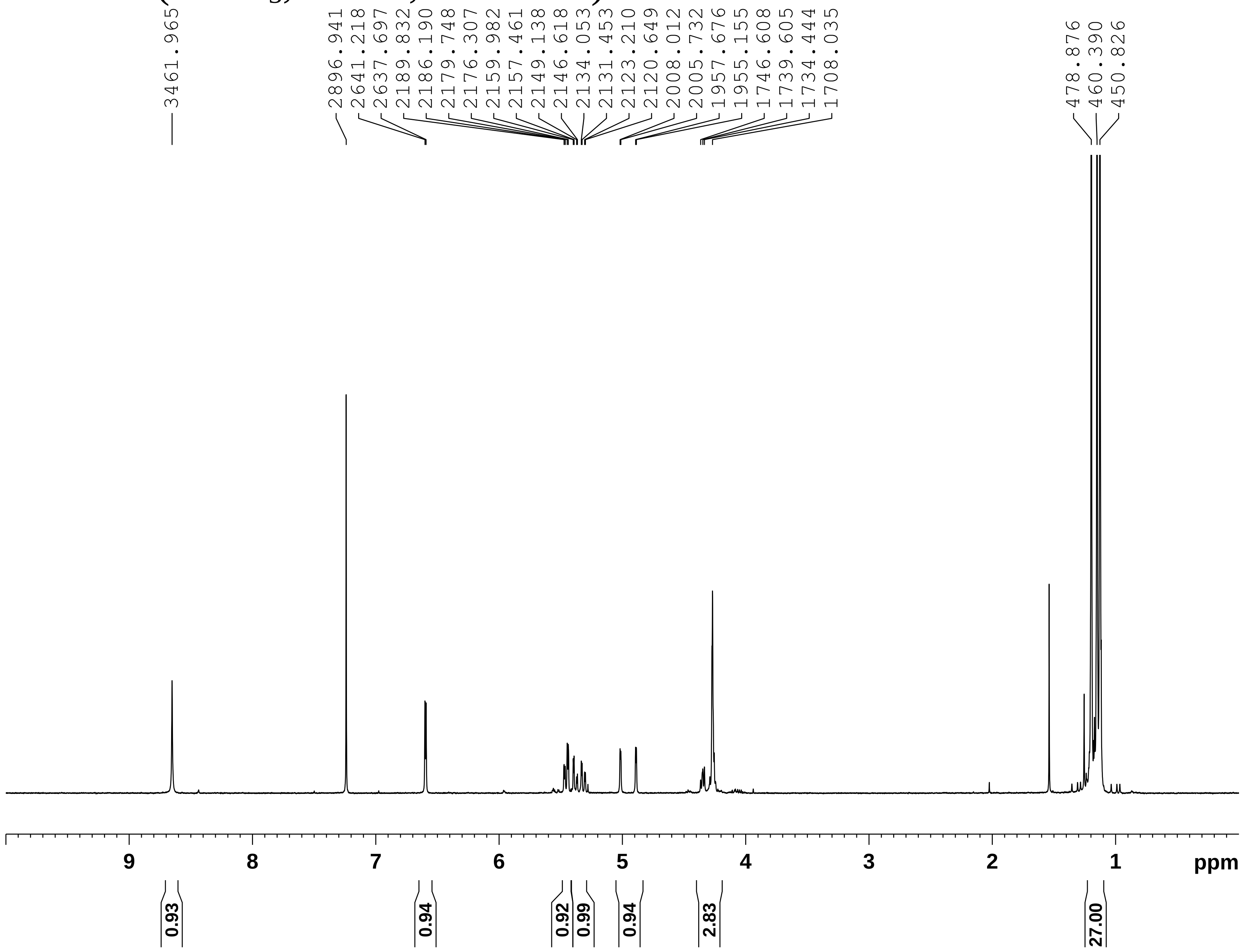

11, ^{13}C NMR ($CDCl_3$, 295 K, 100 MHz)

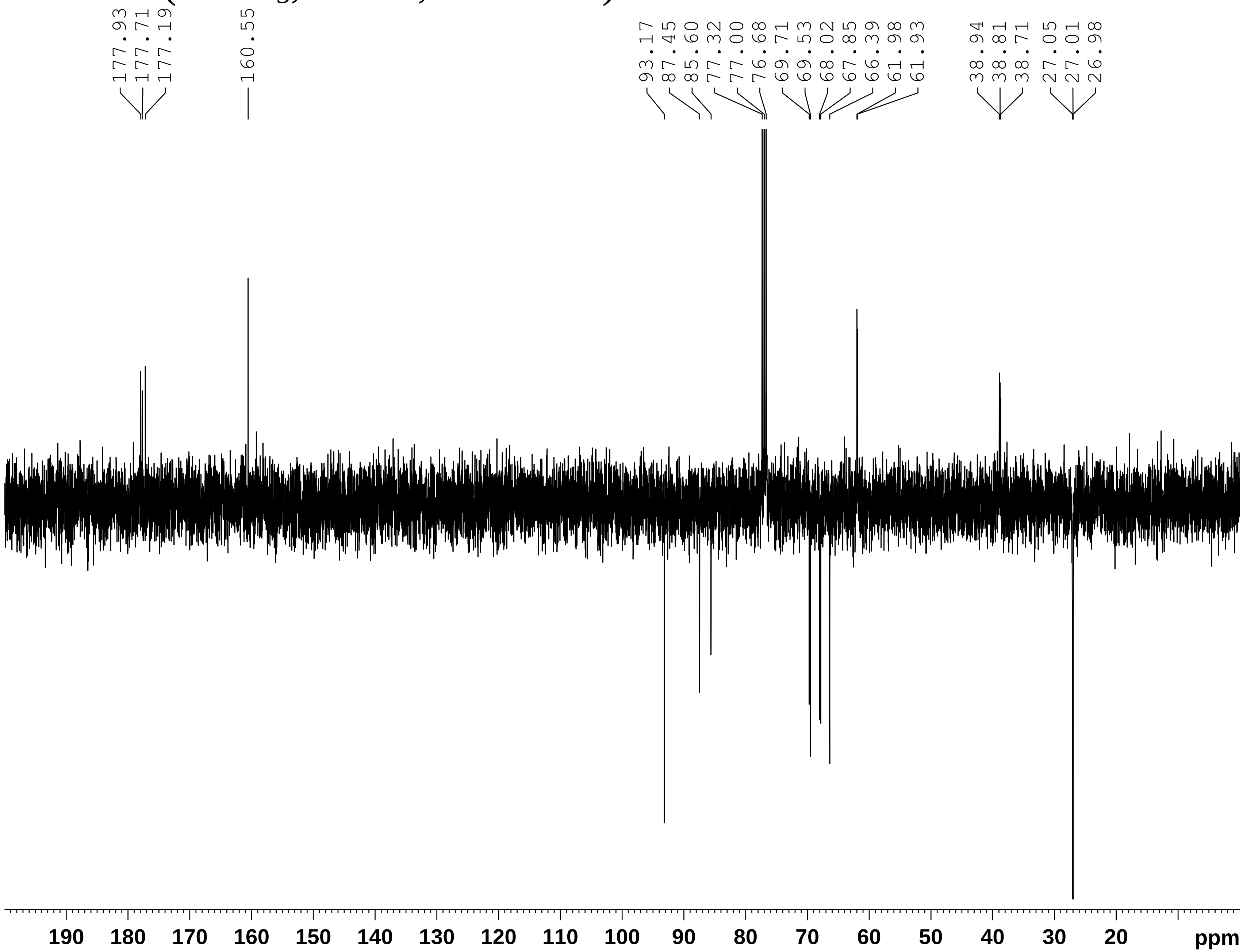

14, ^{1}H NMR (CD_3OD, 295 K, 400 MHz)

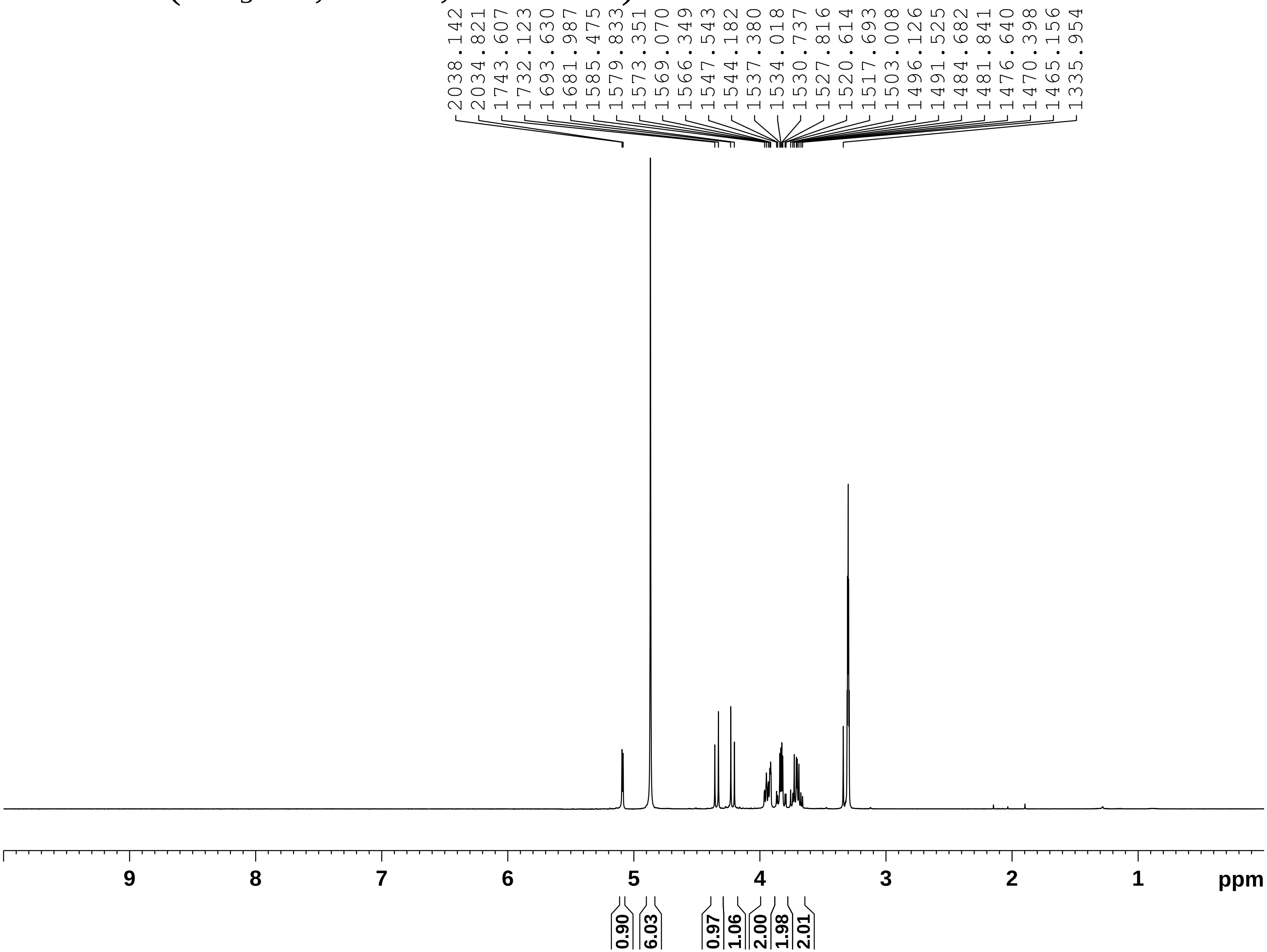

14, ^{13}C NMR (CD_3OD, 295 K, 100 MHz)

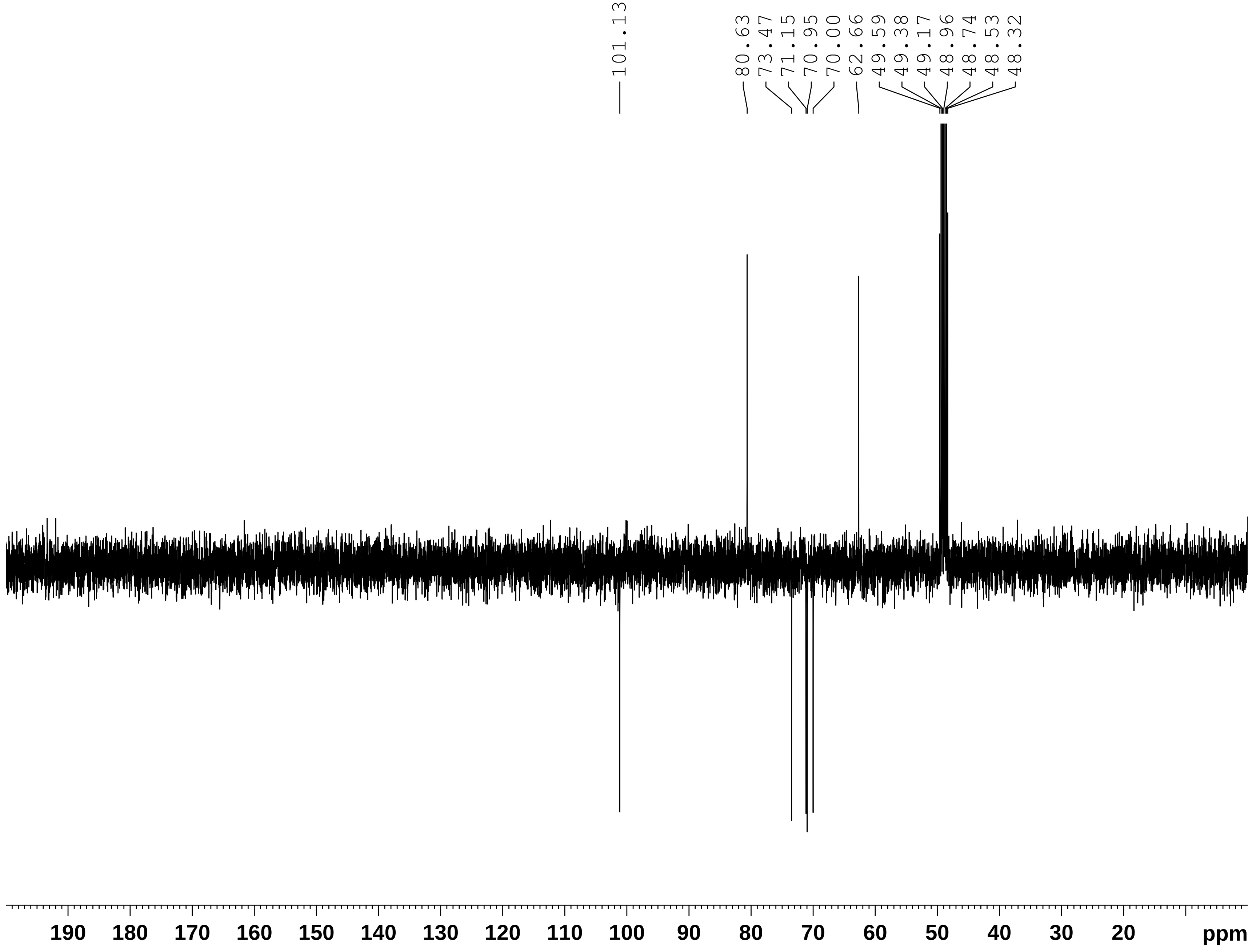

15, ^{1}H NMR ($CDCl_3$, 296 K, 400 MHz)

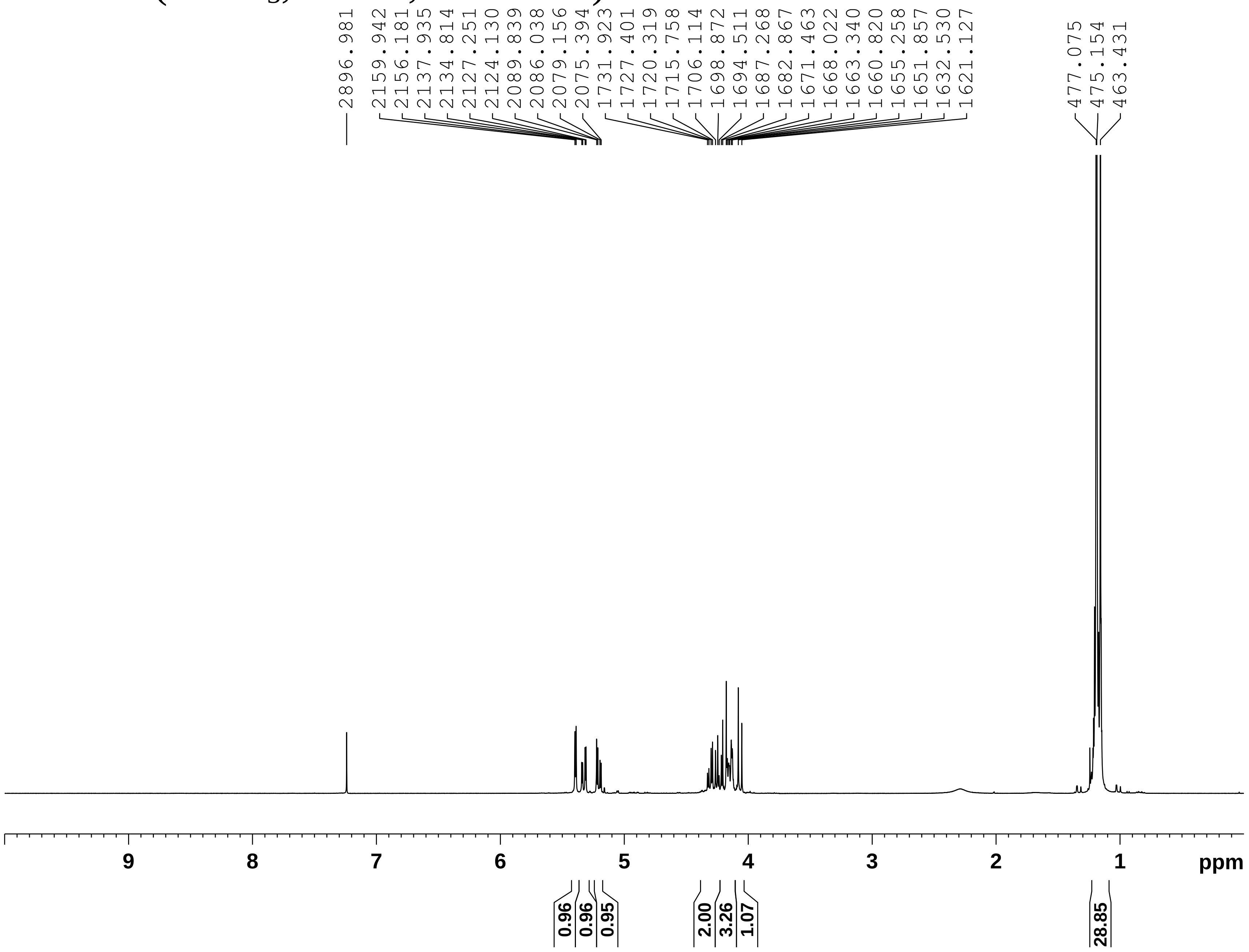

15, ^{13}C NMR ($CDCl_3$, 296 K, 100 MHz)

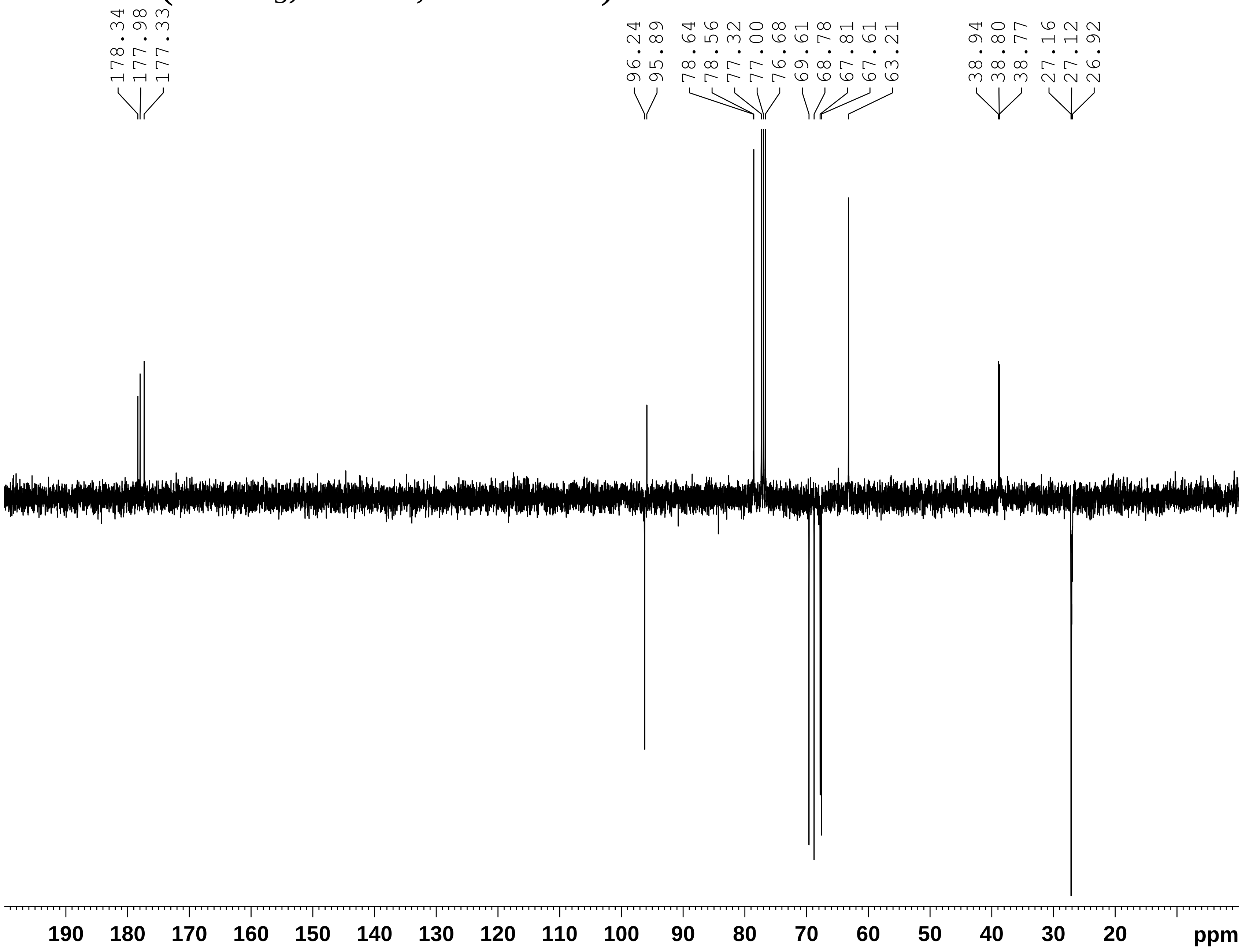

16, ^{1}H NMR ($CDCl_3$, 296 K, 400 MHz)

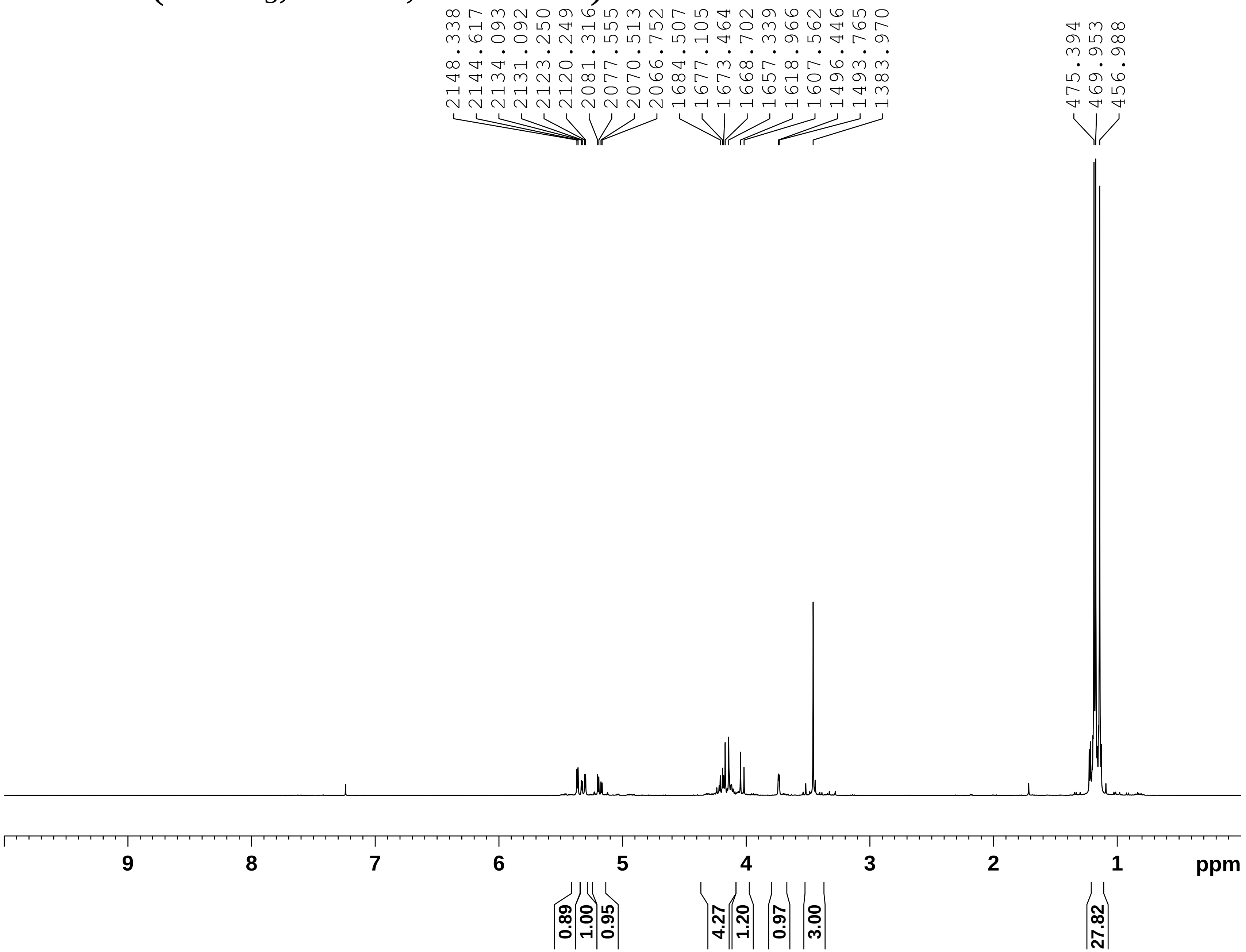

16, ^{13}C NMR ($CDCl_3$, 296 K, 100 MHz)

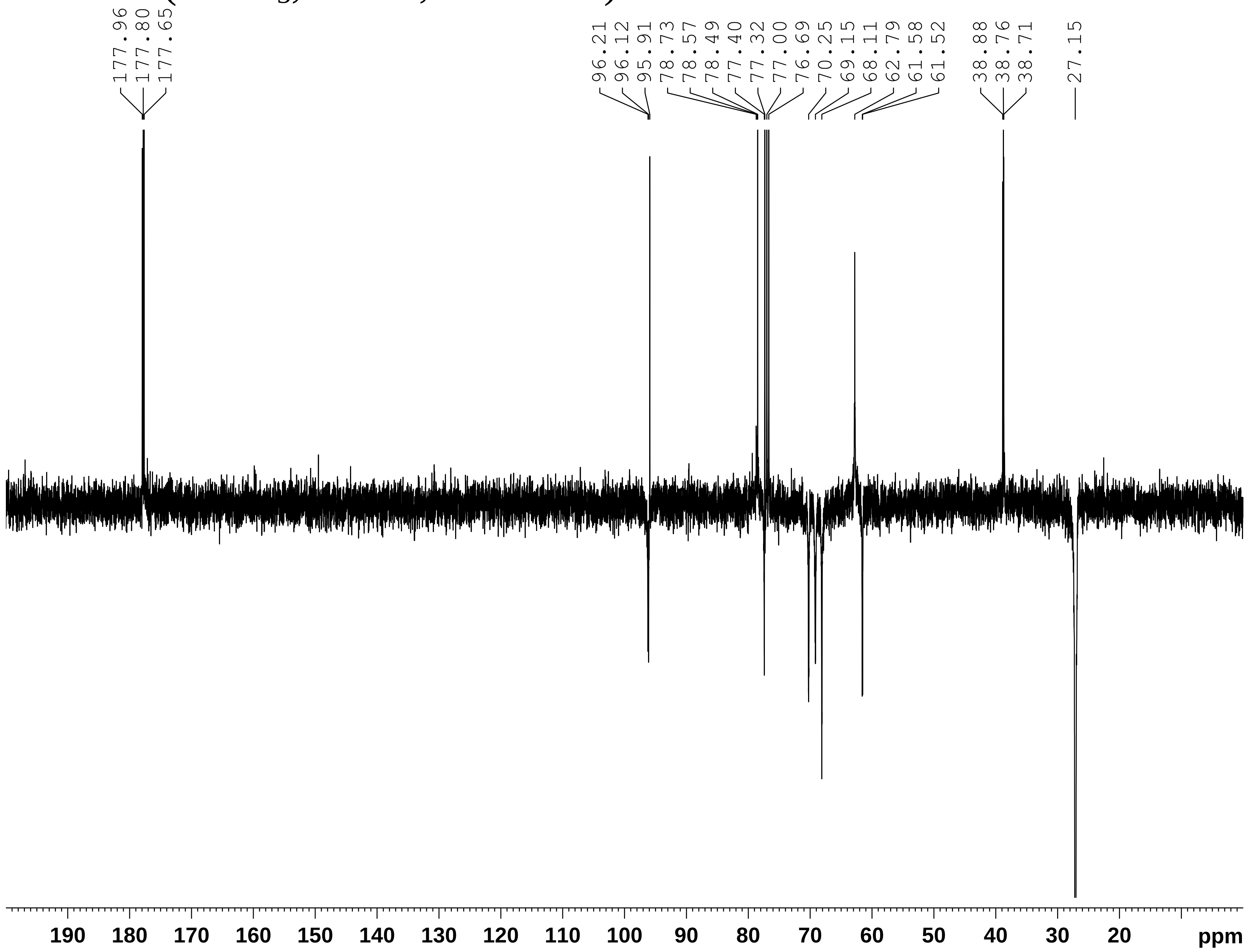

17, ^{1}H NMR ($CDCl_3$, 295 K, 400 MHz)

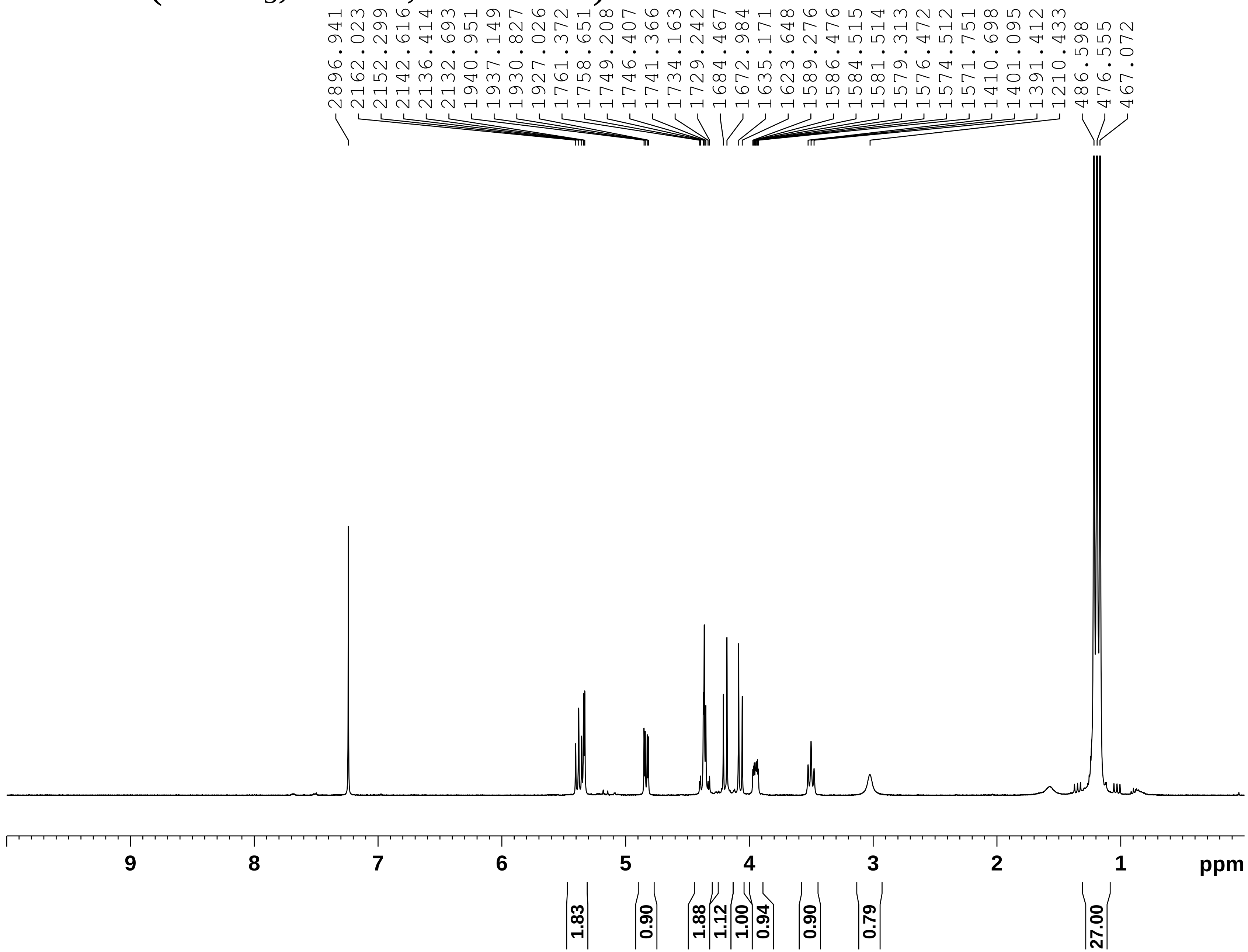

17, ^{13}C NMR ($CDCl_3$, 295 K, 100 MHz)

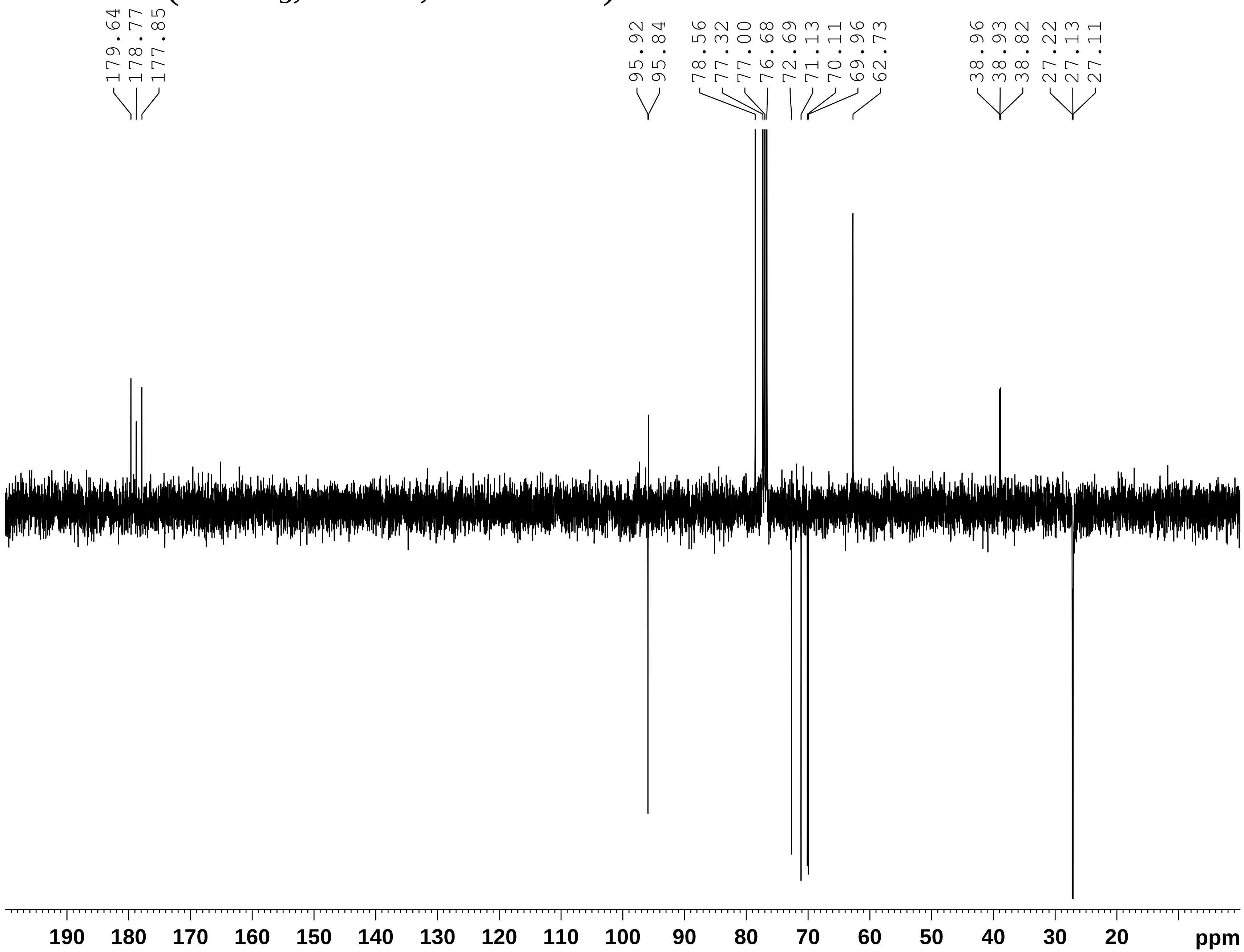

18, ^{1}H NMR ($CDCl_3$, 295 K, 400 MHz)

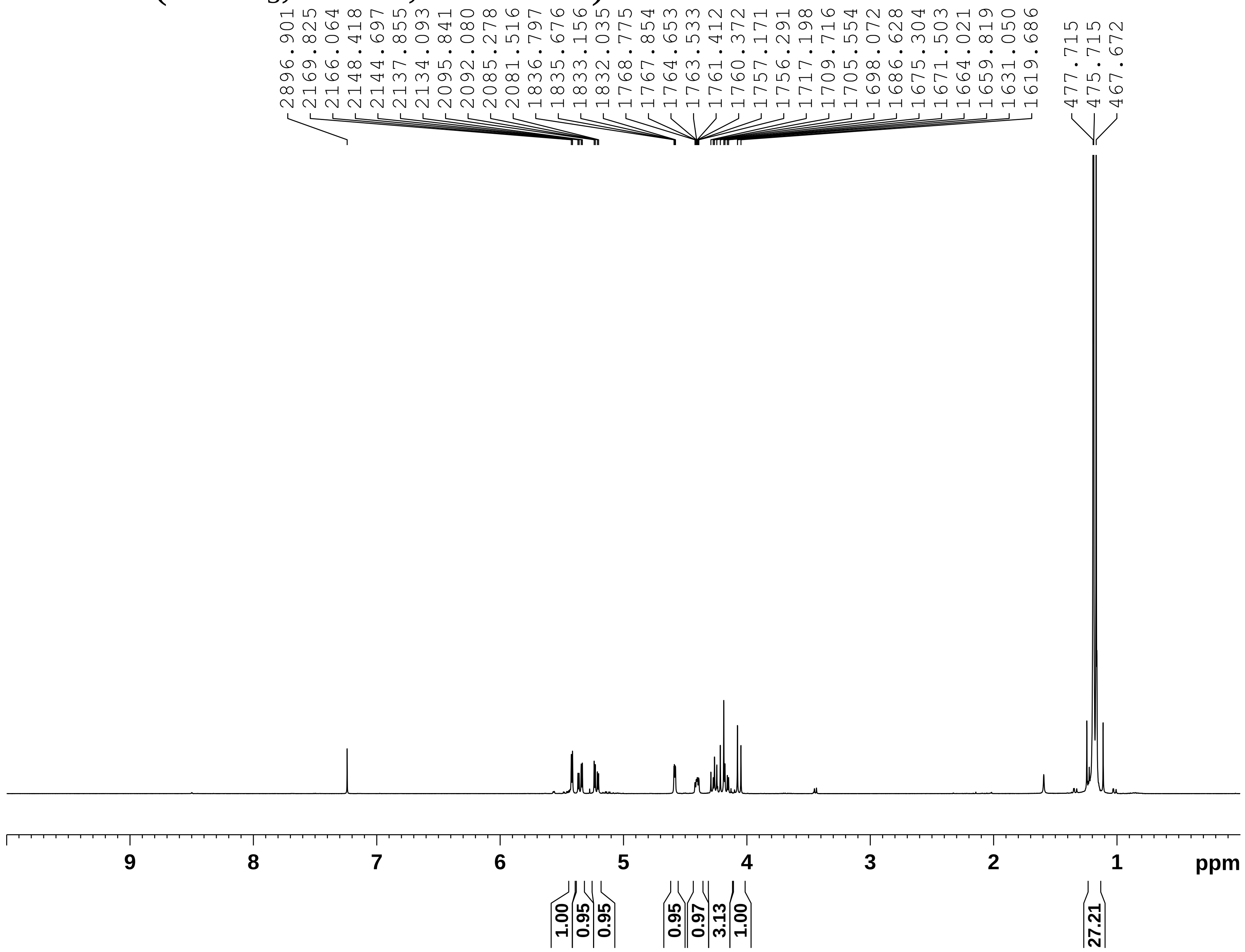

18, ^{13}C NMR ($CDCl_3$, 295 K, 100 MHz)

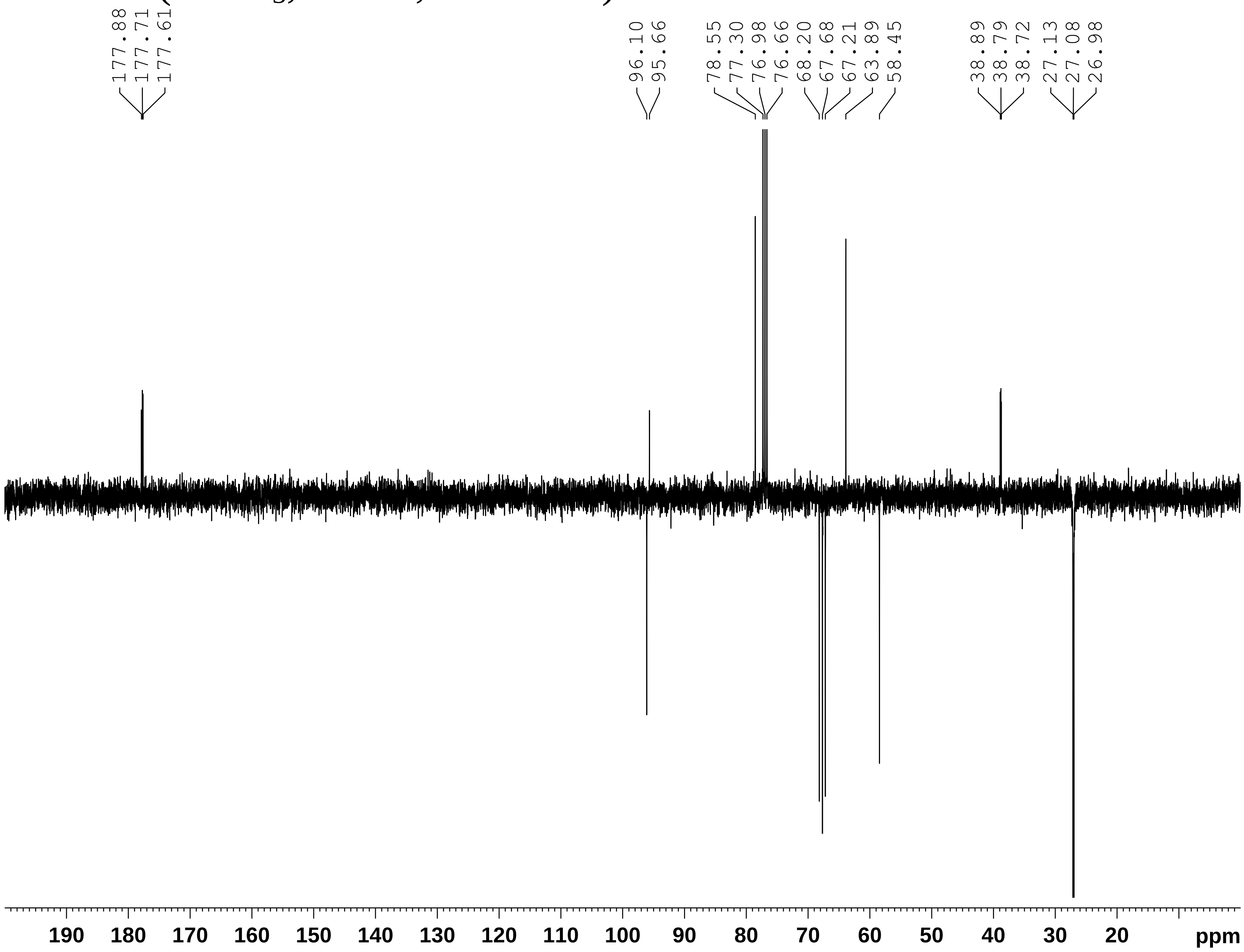

19, ^{1}H NMR ($CDCl_3$, 295 K, 400 MHz)

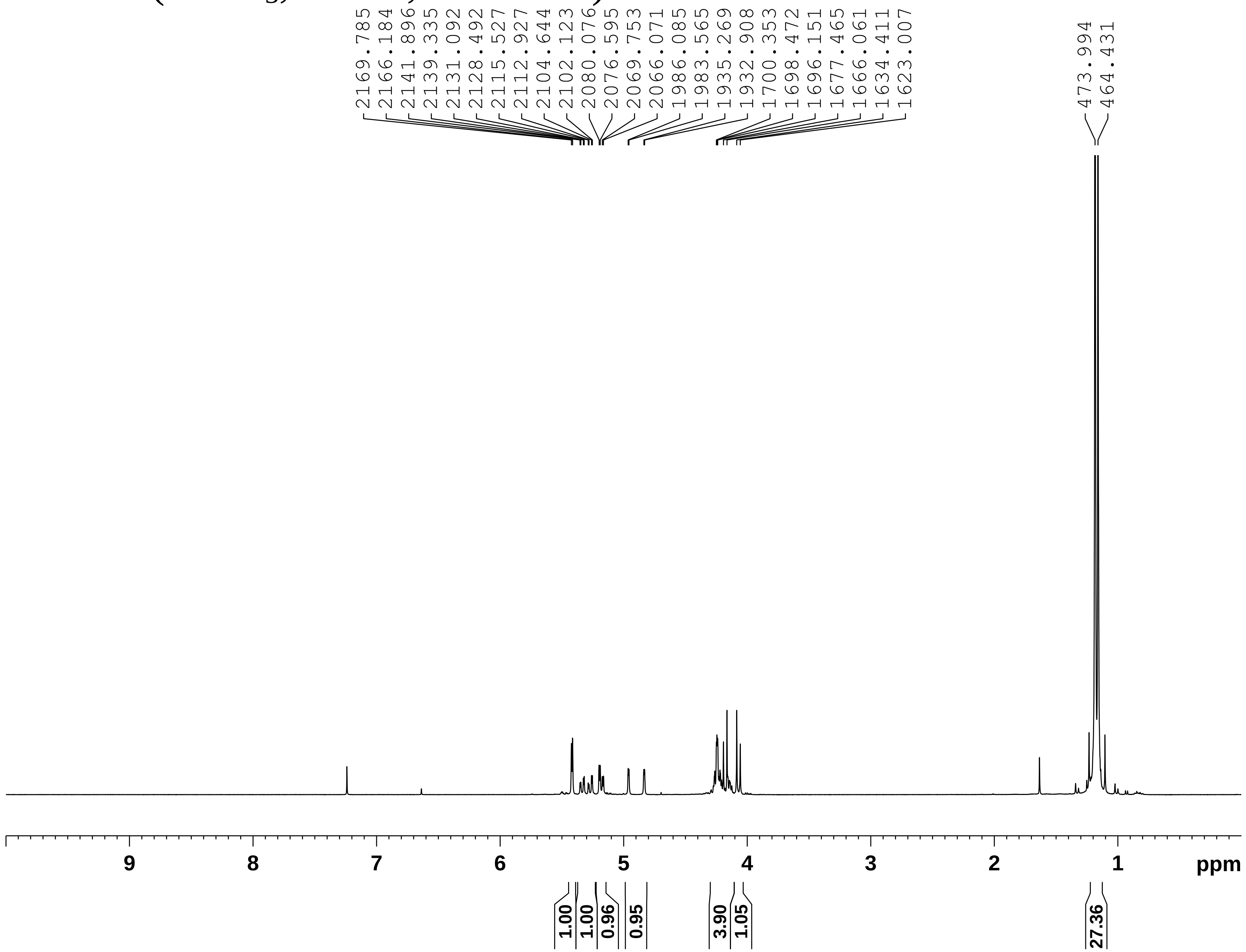

19, ^{13}C NMR ($CDCl_3$, 295 K, 100 MHz)

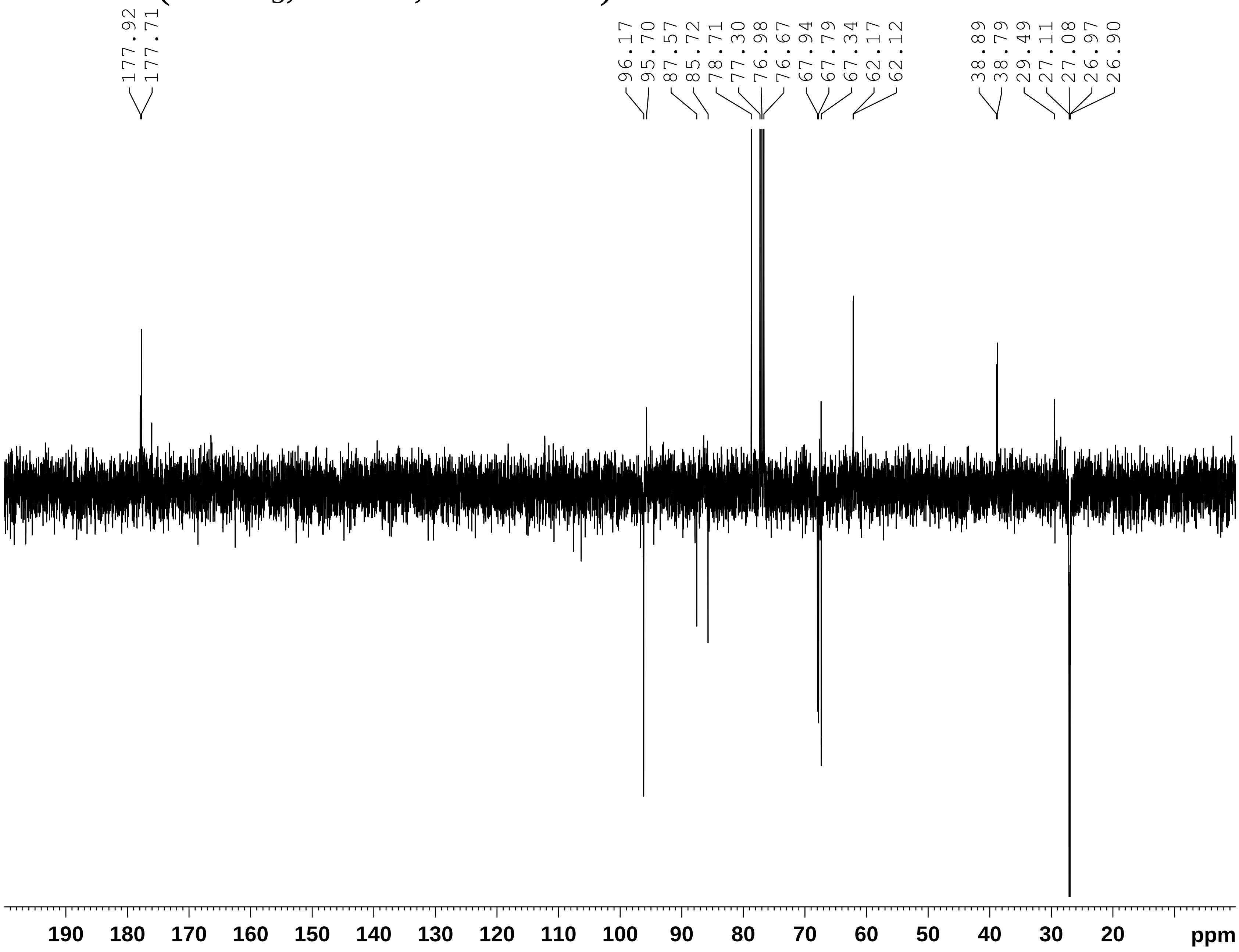

20, ^{1}H NMR ($CDCl_3$, 296 K, 400 MHz)

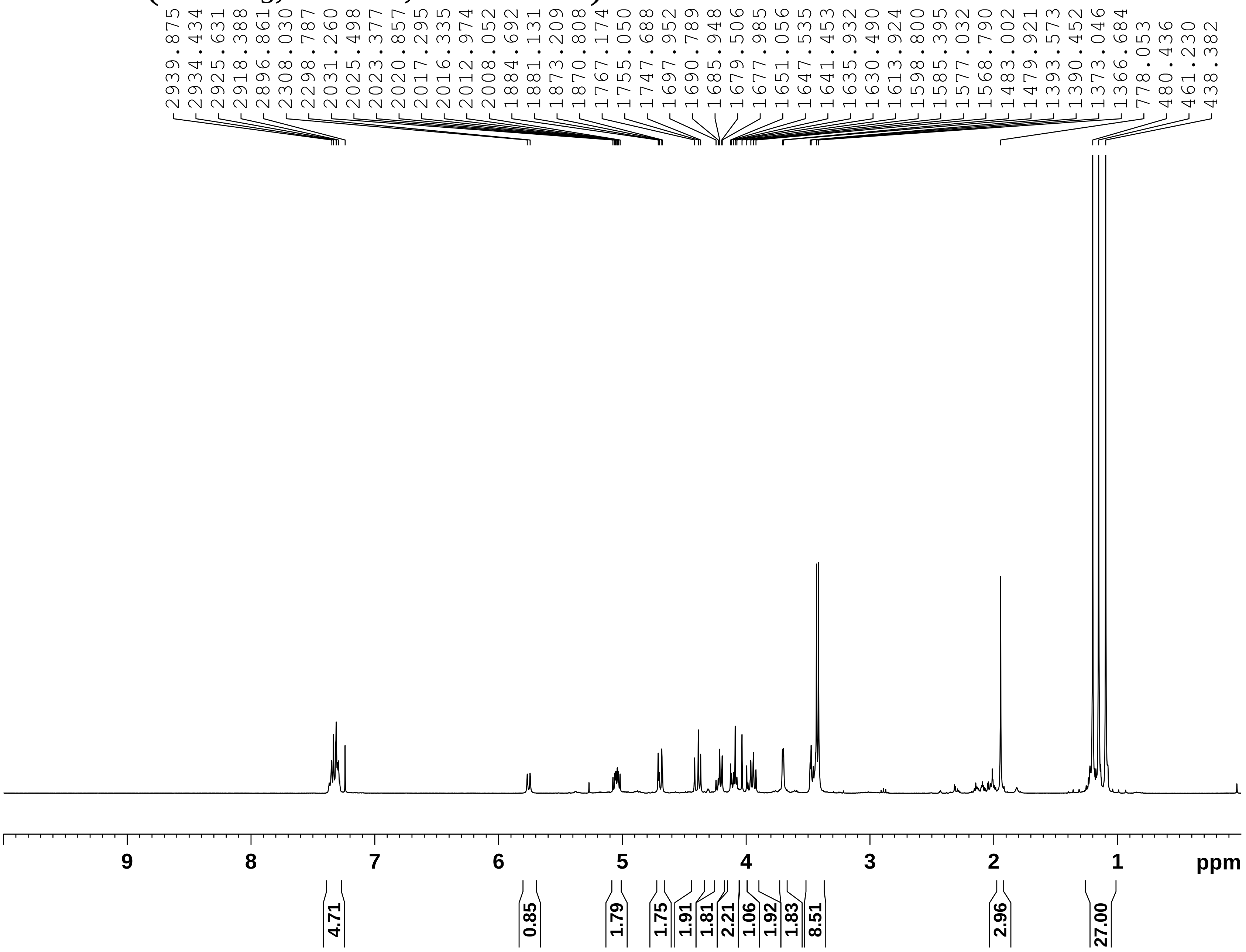

20, ^{13}C NMR ($CDCl_3$, 296 K, 100 MHz)

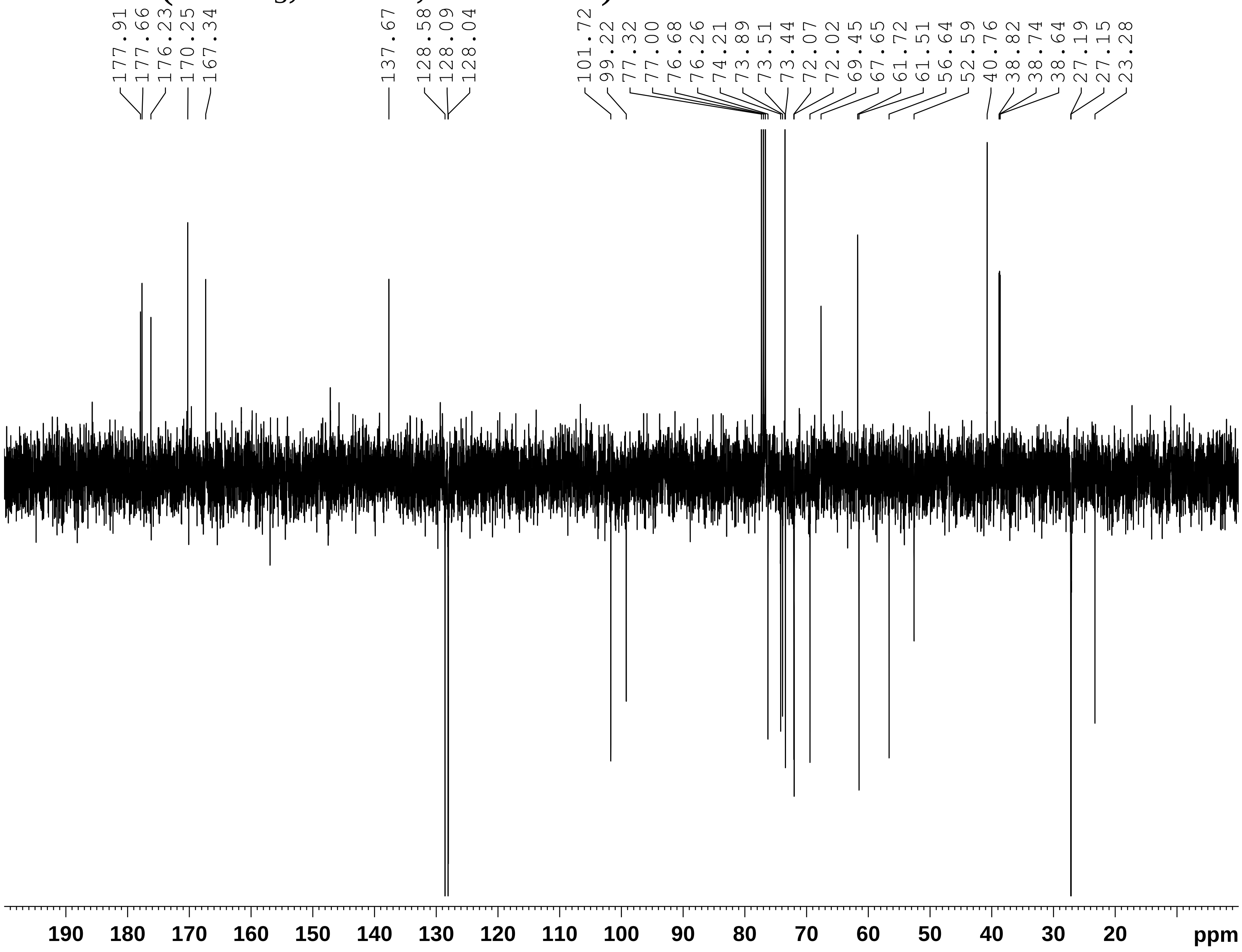

21, ^{1}H NMR ($CDCl_3$, 295 K, 400 MHz)

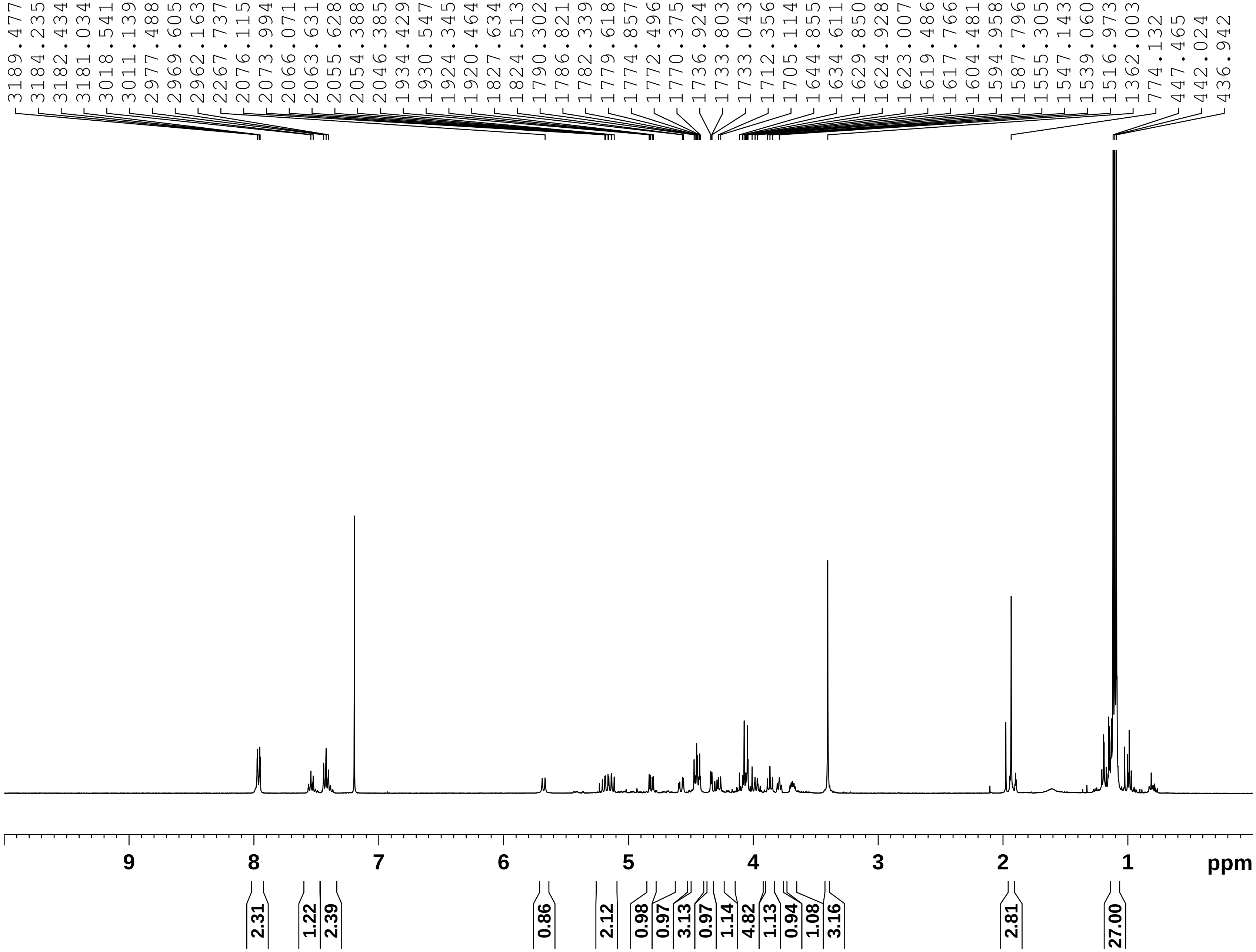

21, ^{13}C NMR ($CDCl_3$, 295 K, 100 MHz)

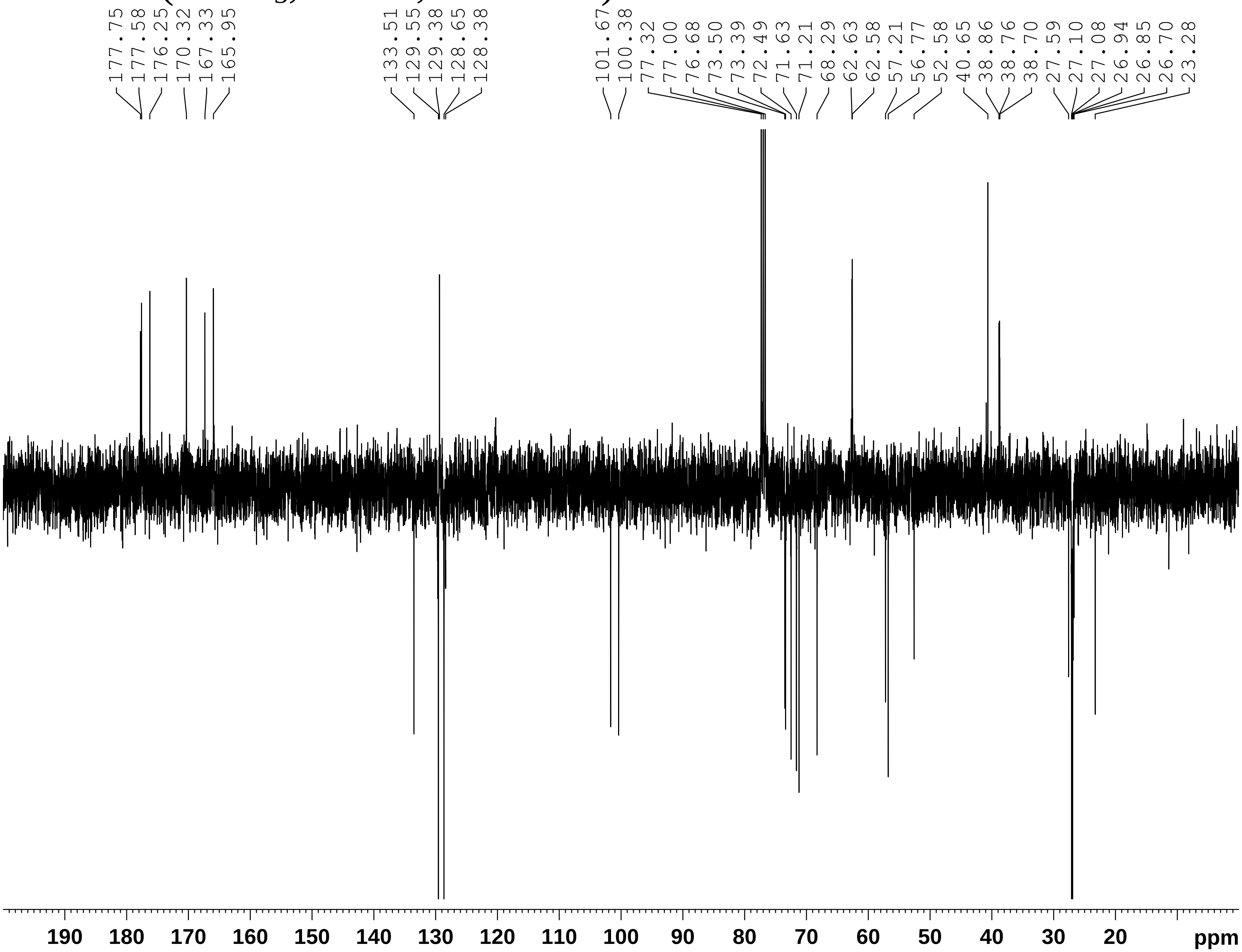

22, ^{1}H NMR ($CDCl_3$, 295 K, 400 MHz)

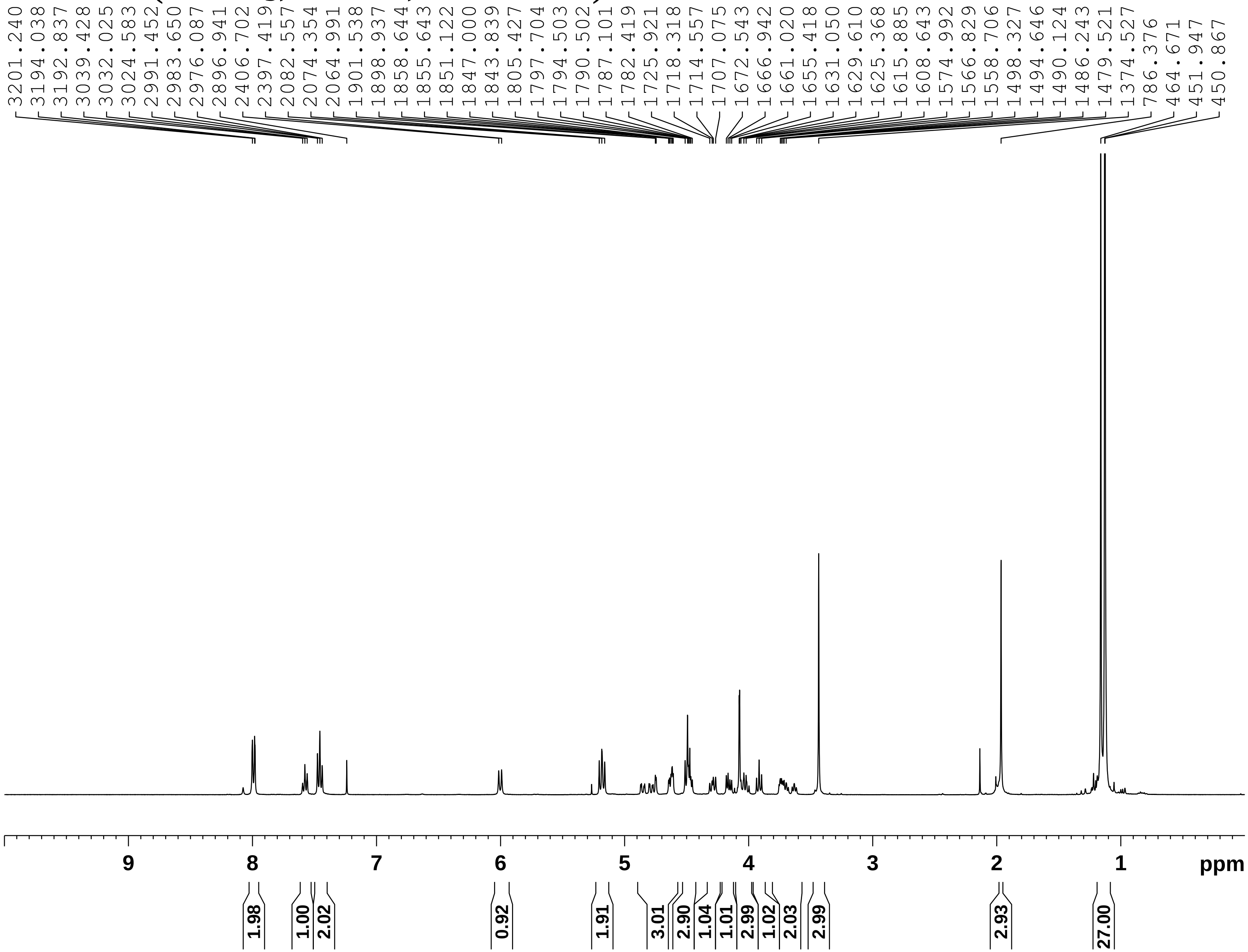

22, ^{13}C NMR ($CDCl_3$, 295 K, 100 MHz)

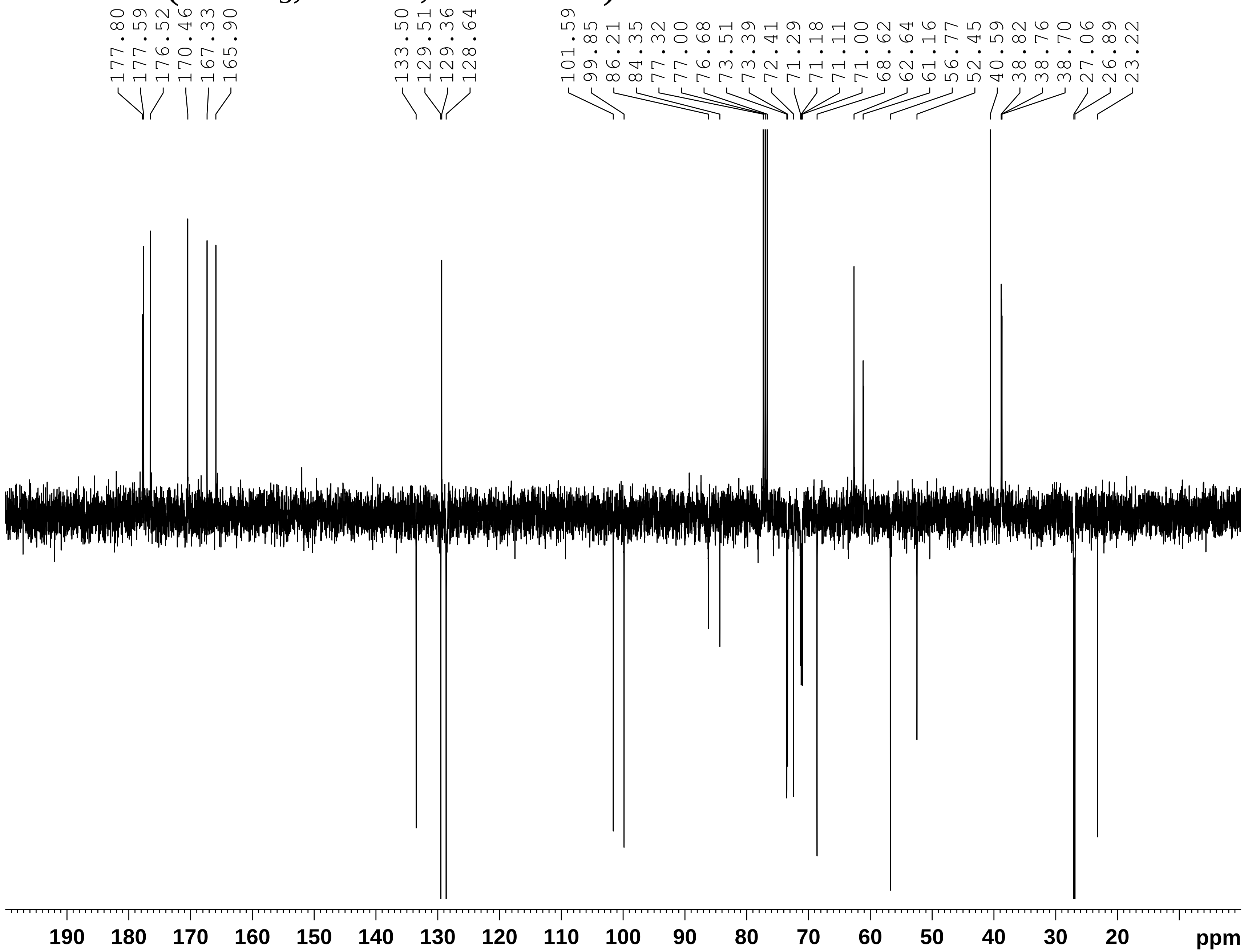

23, ^{1}H NMR ($CDCl_3$, 296 K, 400 MHz)

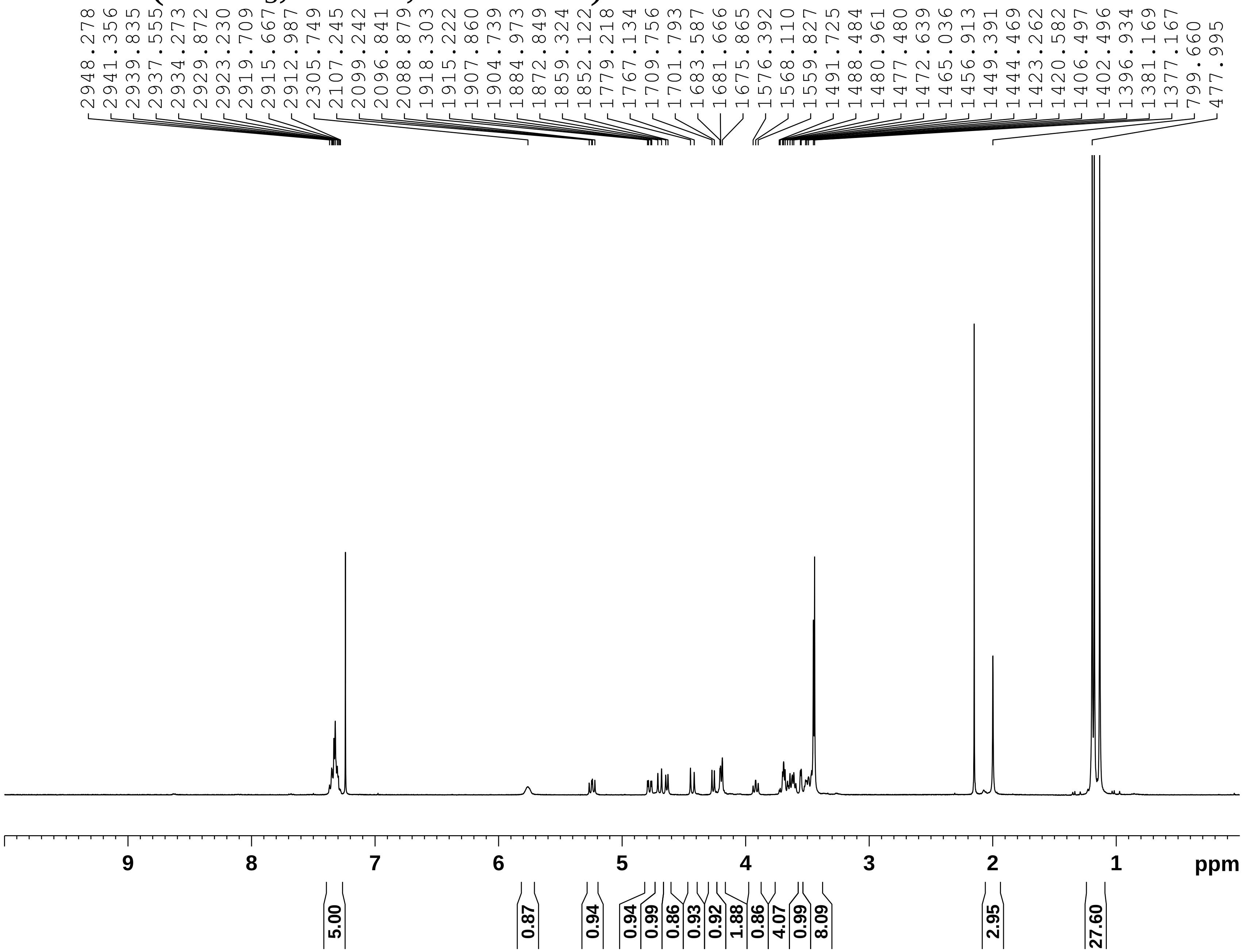

23, ^{13}C NMR ($CDCl_3$, 296 K, 100 MHz)

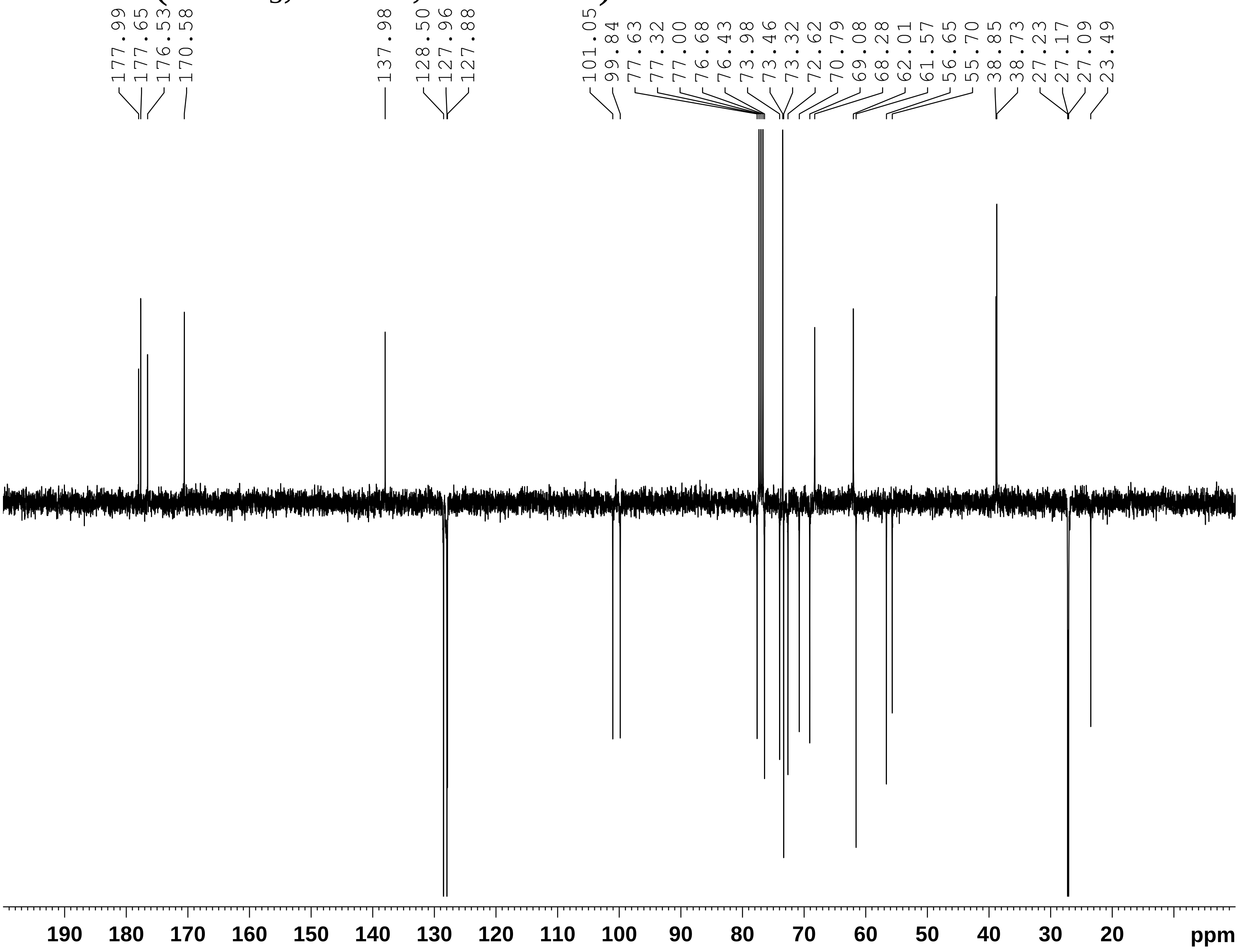

24, ^{1}H NMR ($CDCl_3$, 300 K, 400 MHz)

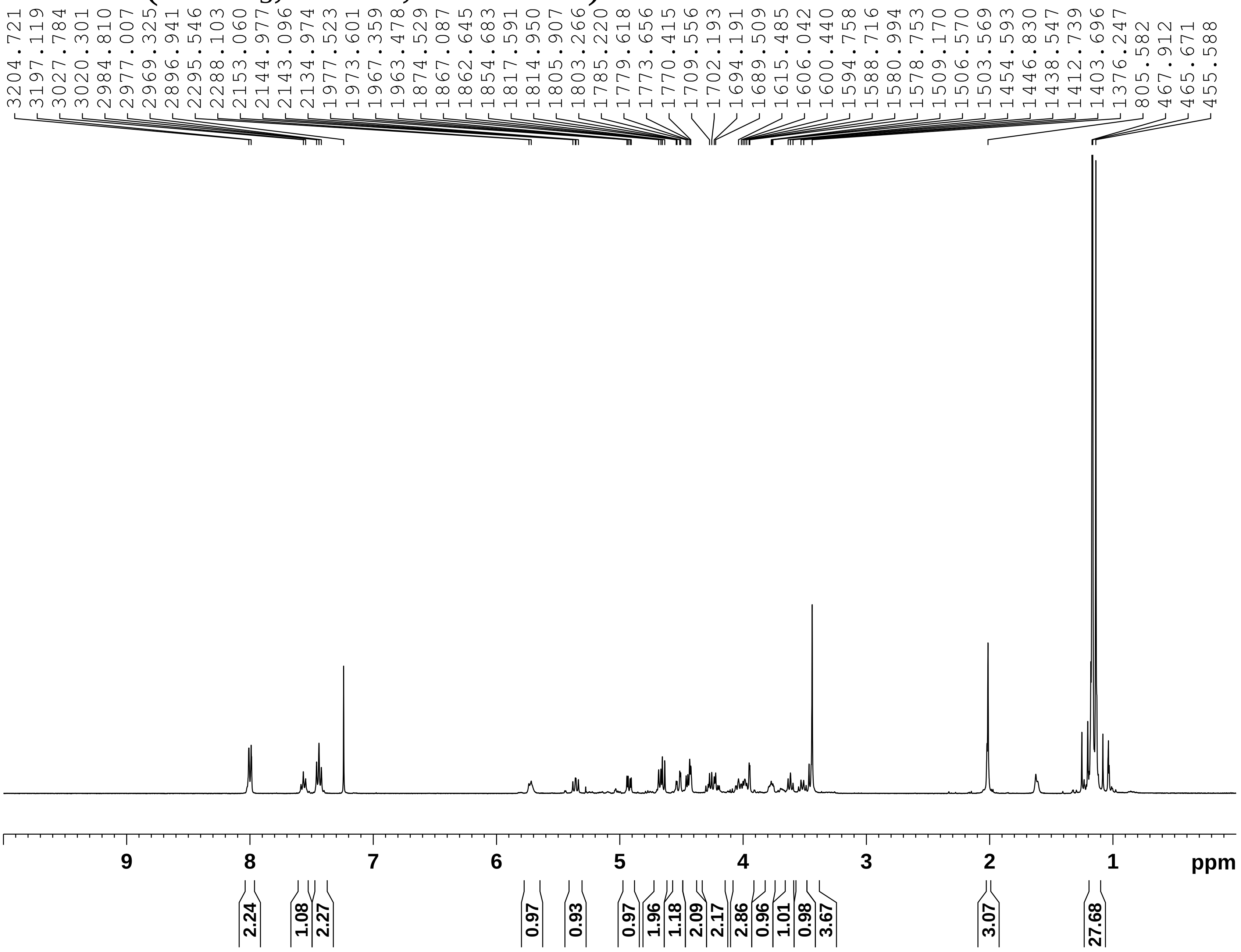

24, ^{13}C NMR ($CDCl_3$, 300 K, 100 MHz)

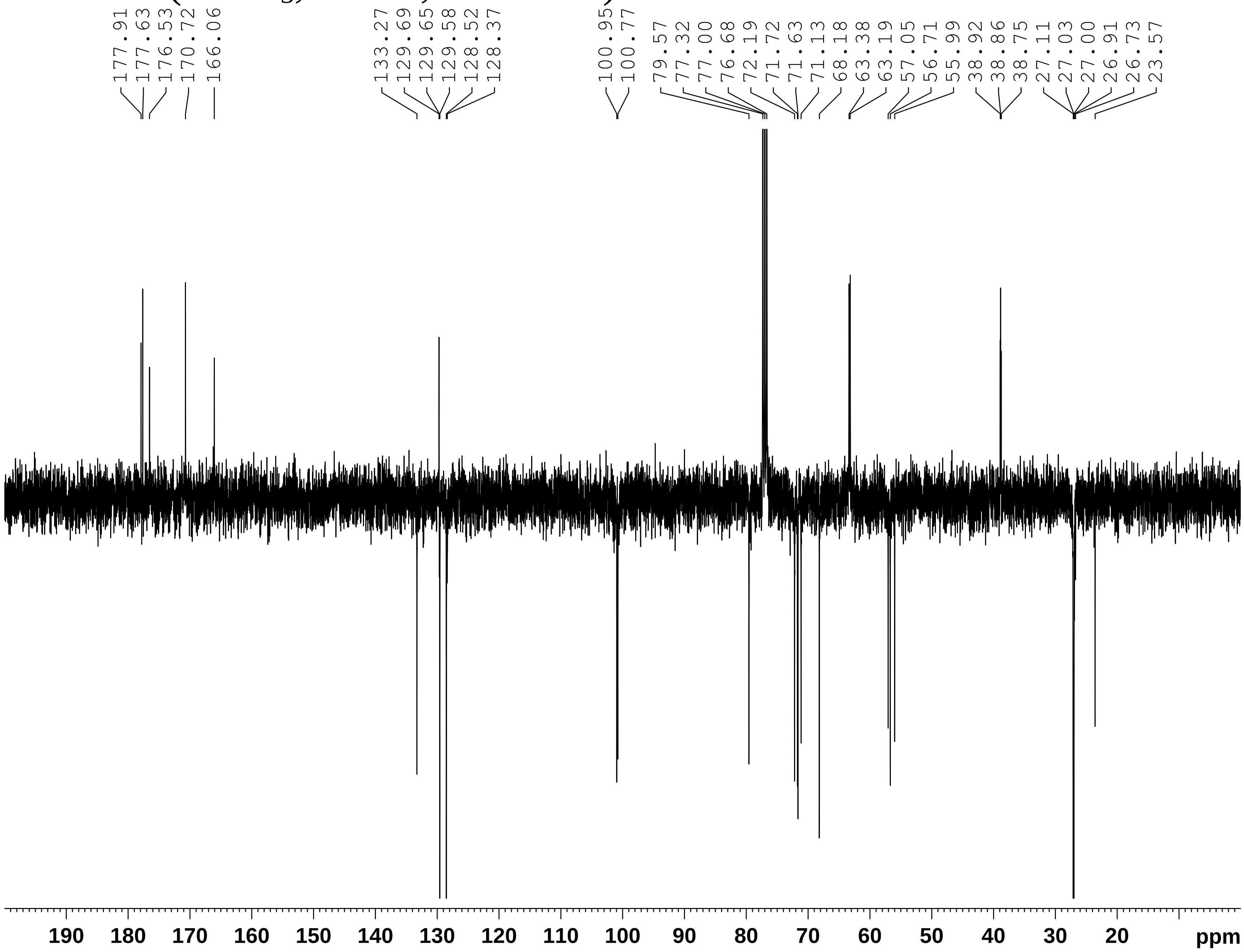

25, ^{1}H NMR ($CDCl_3$, 295 K, 400 MHz)

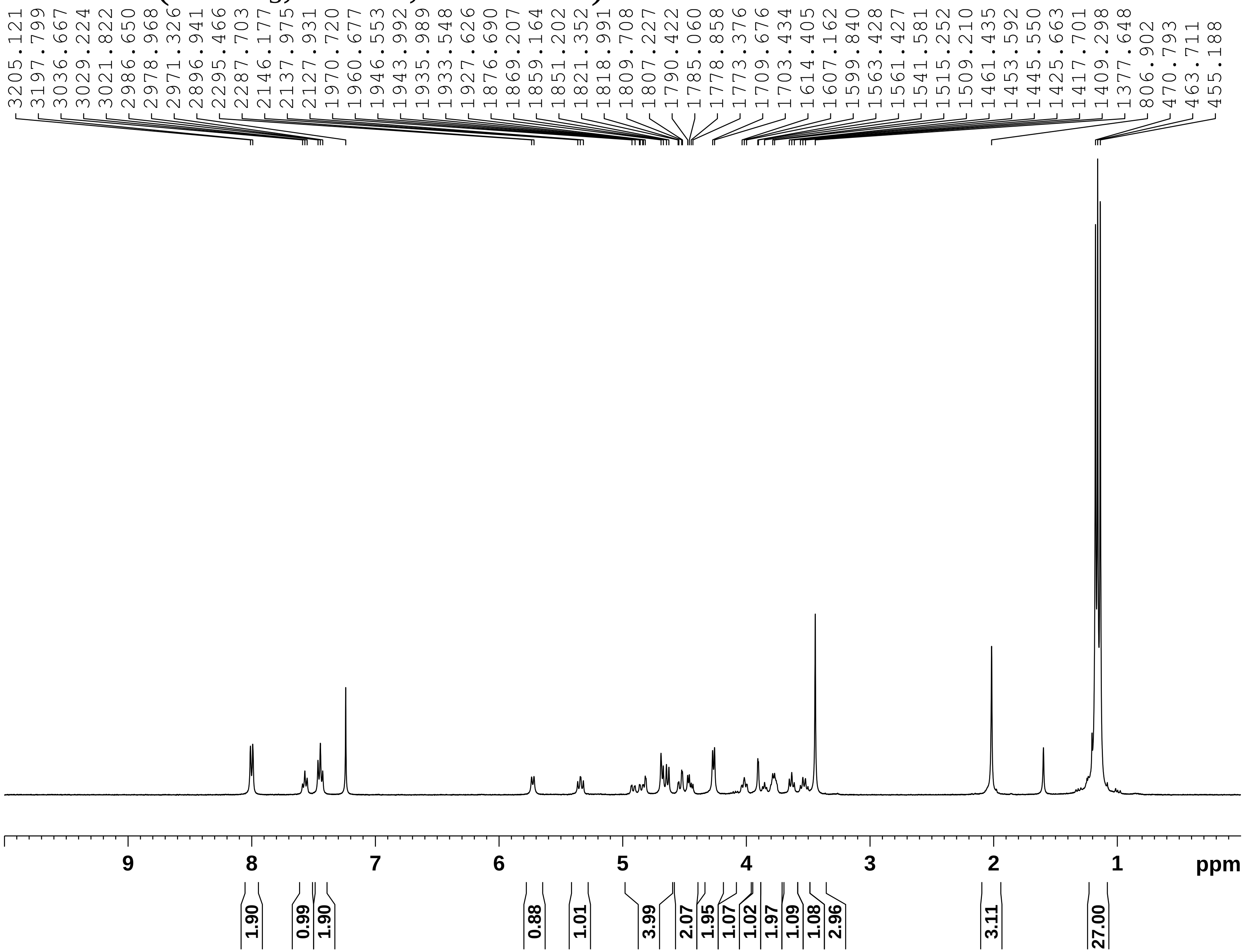

25, ^{13}C NMR ($CDCl_3$, 295 K, 100 MHz)

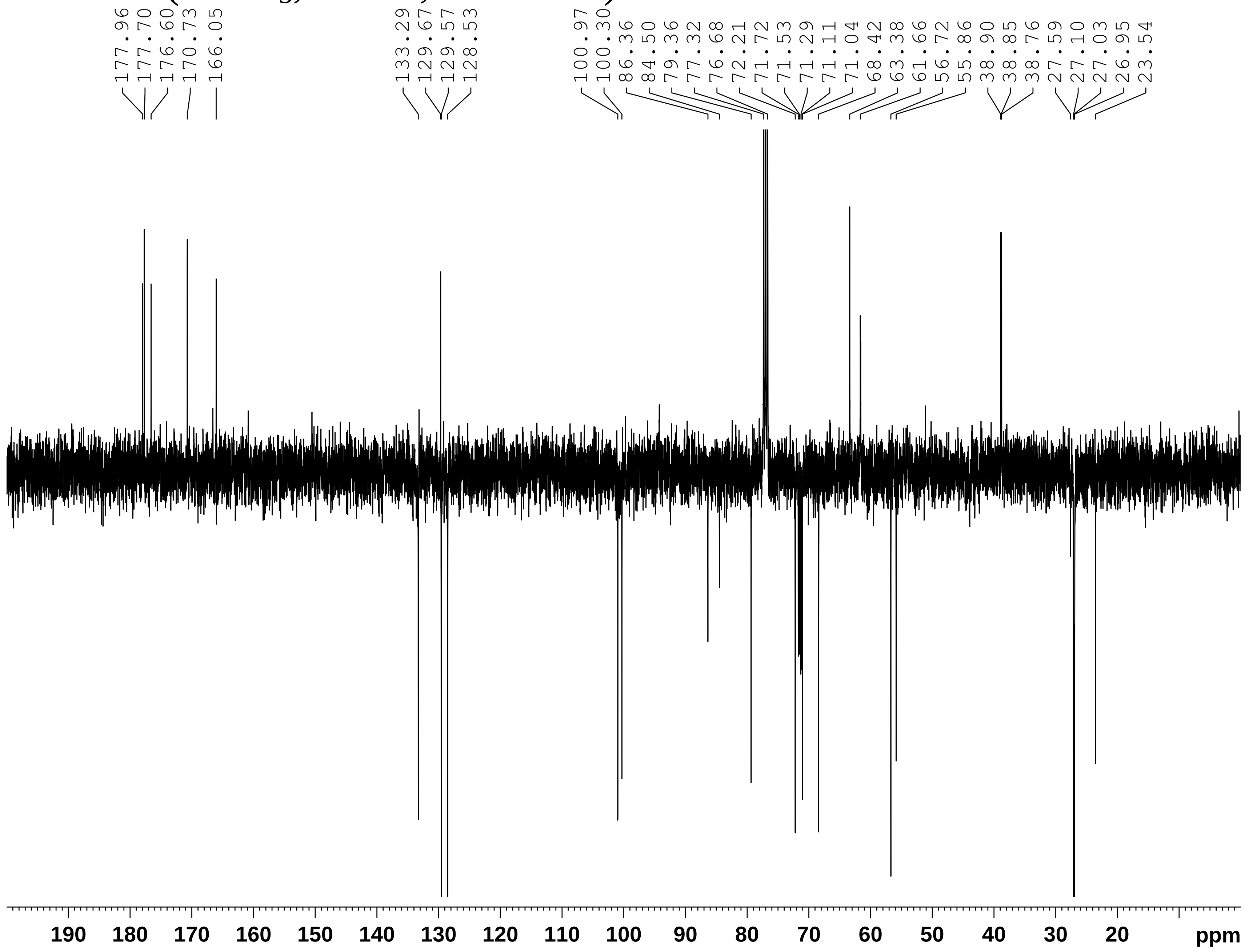

26, ^{1}H NMR ($CDCl_3$, 296 K, 400 MHz)

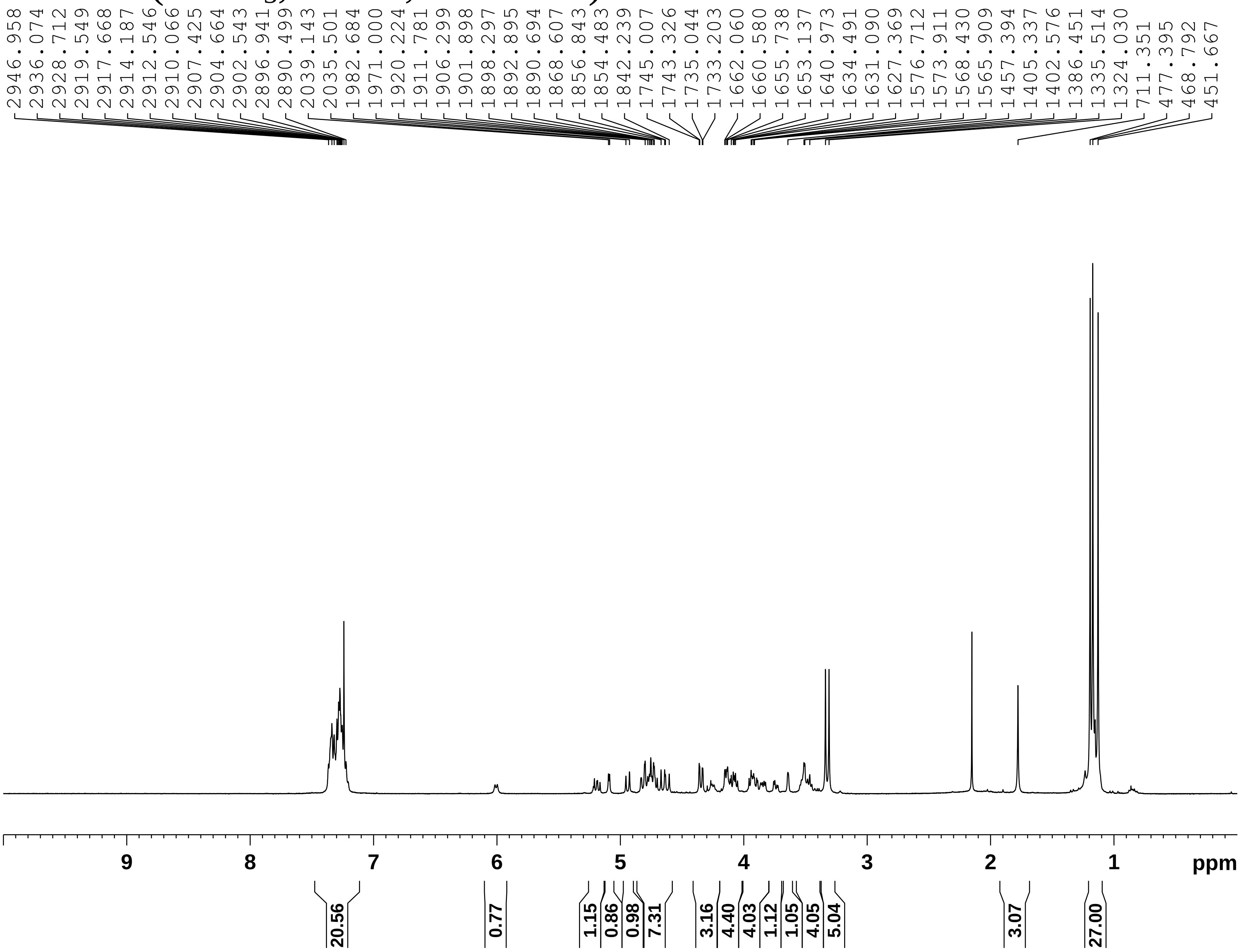

26, ^{13}C NMR ($CDCl_3$, 296 K, 100 MHz)

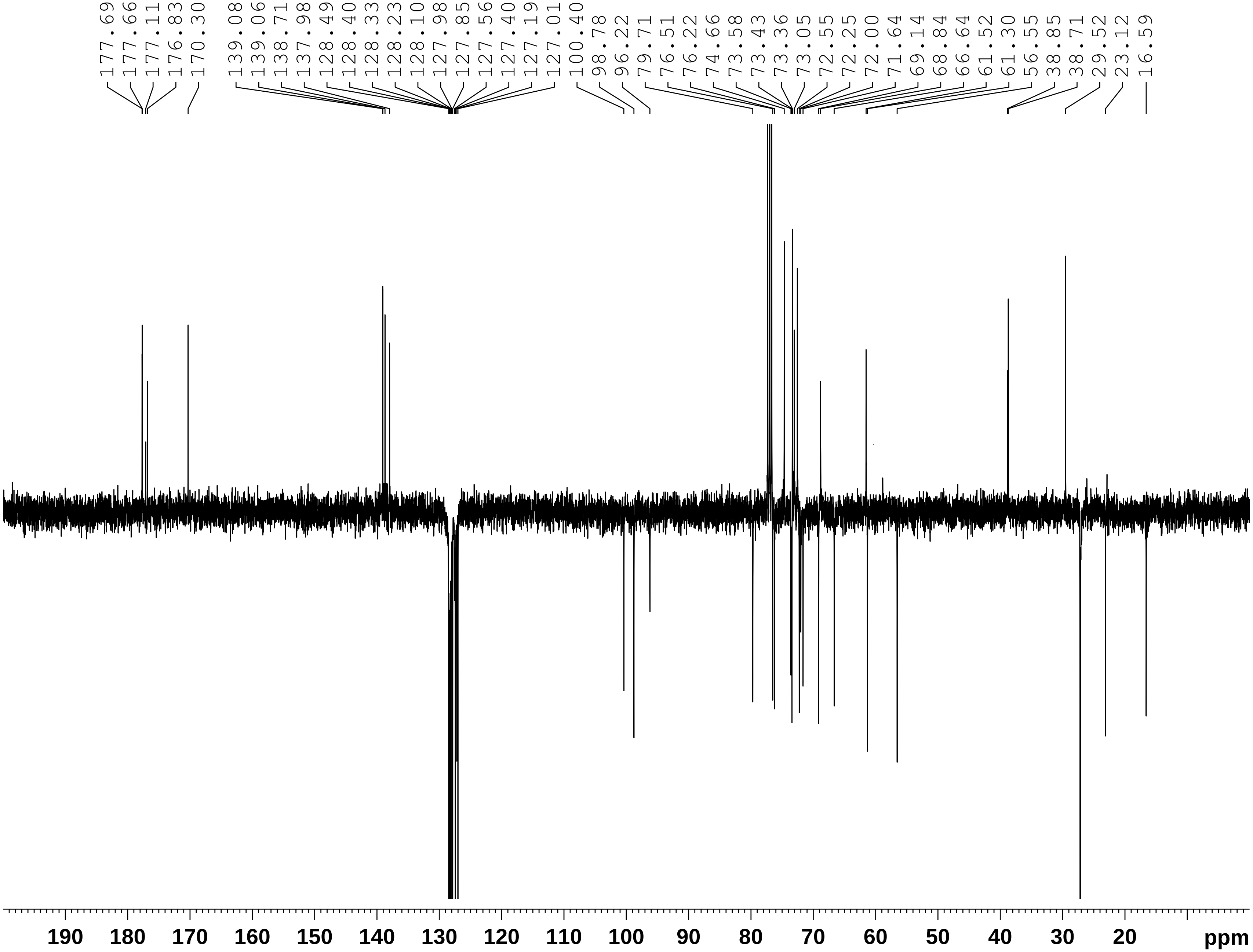

27, ^{1}H NMR ($CDCl_3$, 295 K, 400 MHz)

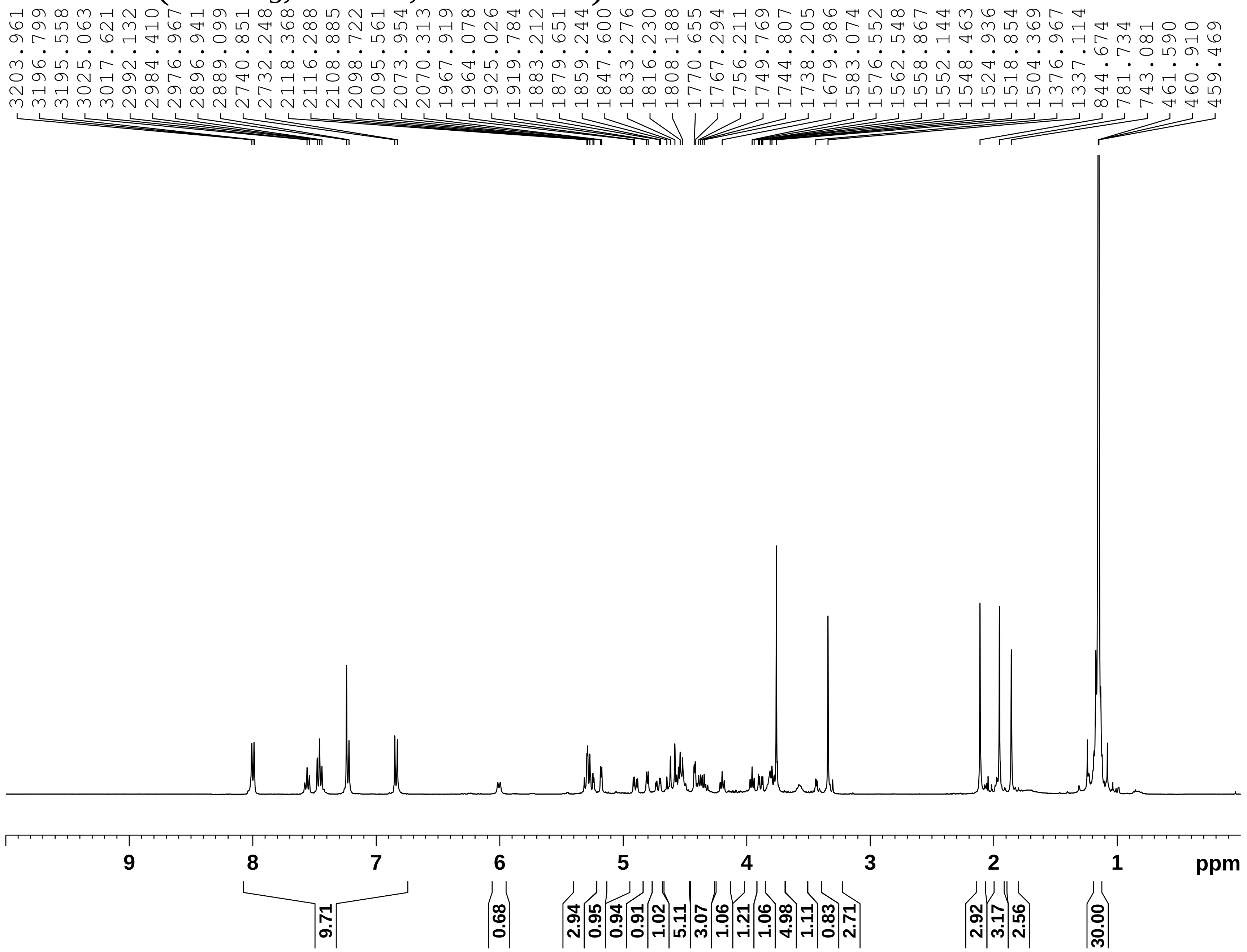

27, ^{13}C NMR ($CDCl_3$, 295 K, 100 MHz)

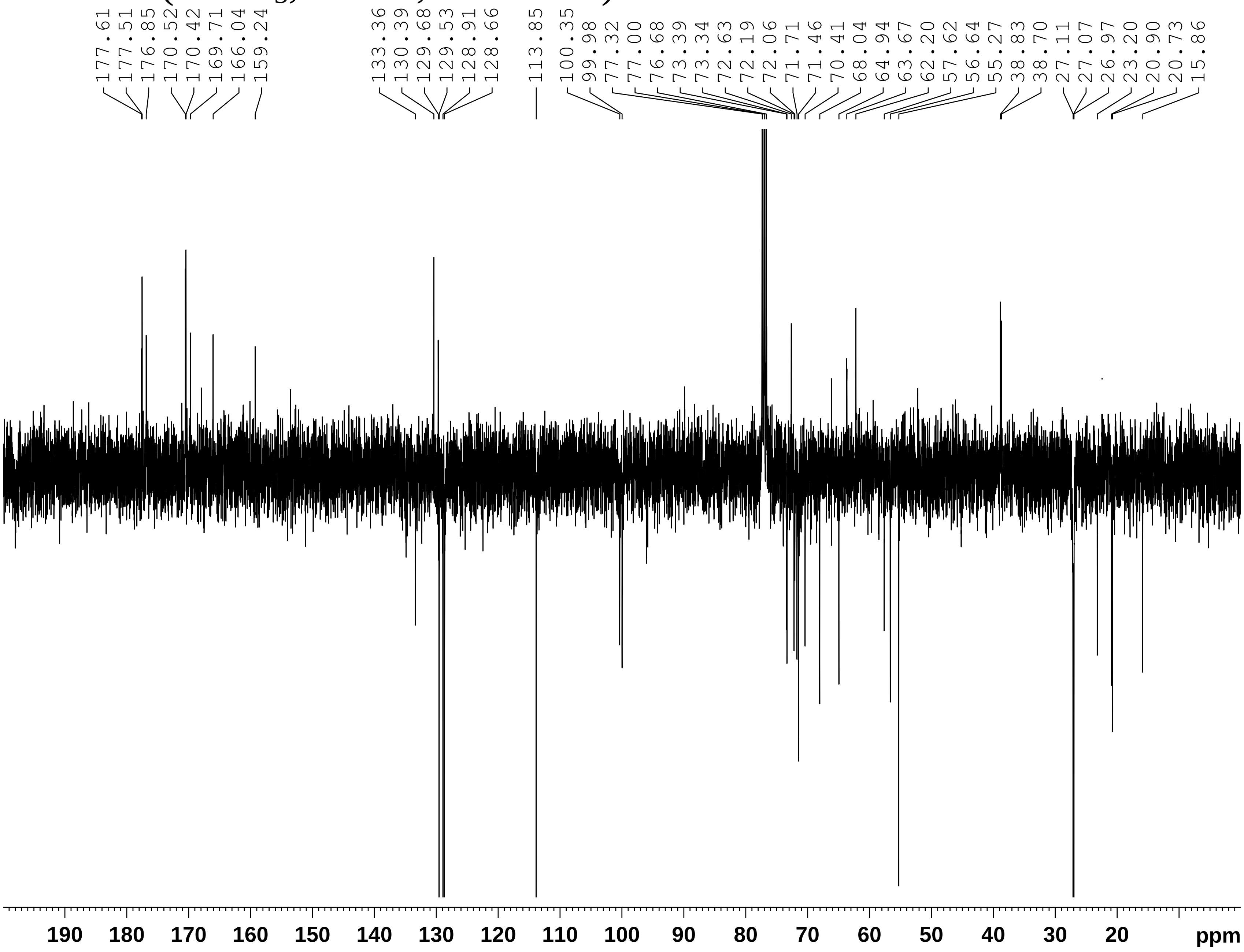

28, ^{1}H NMR ($CDCl_3$, 295 K, 400 MHz)

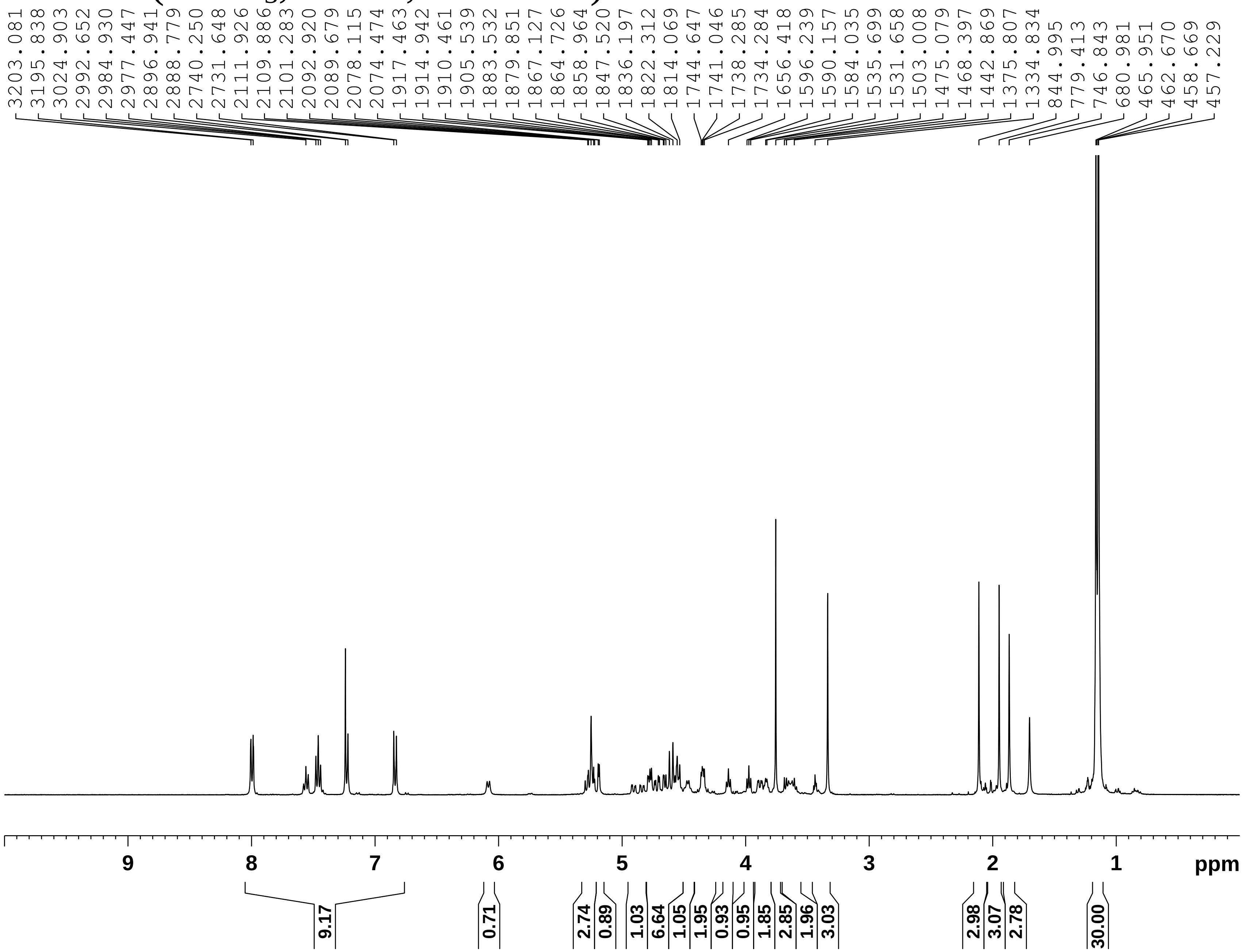

28, ^{13}C NMR ($CDCl_3$, 295 K, 100 MHz)

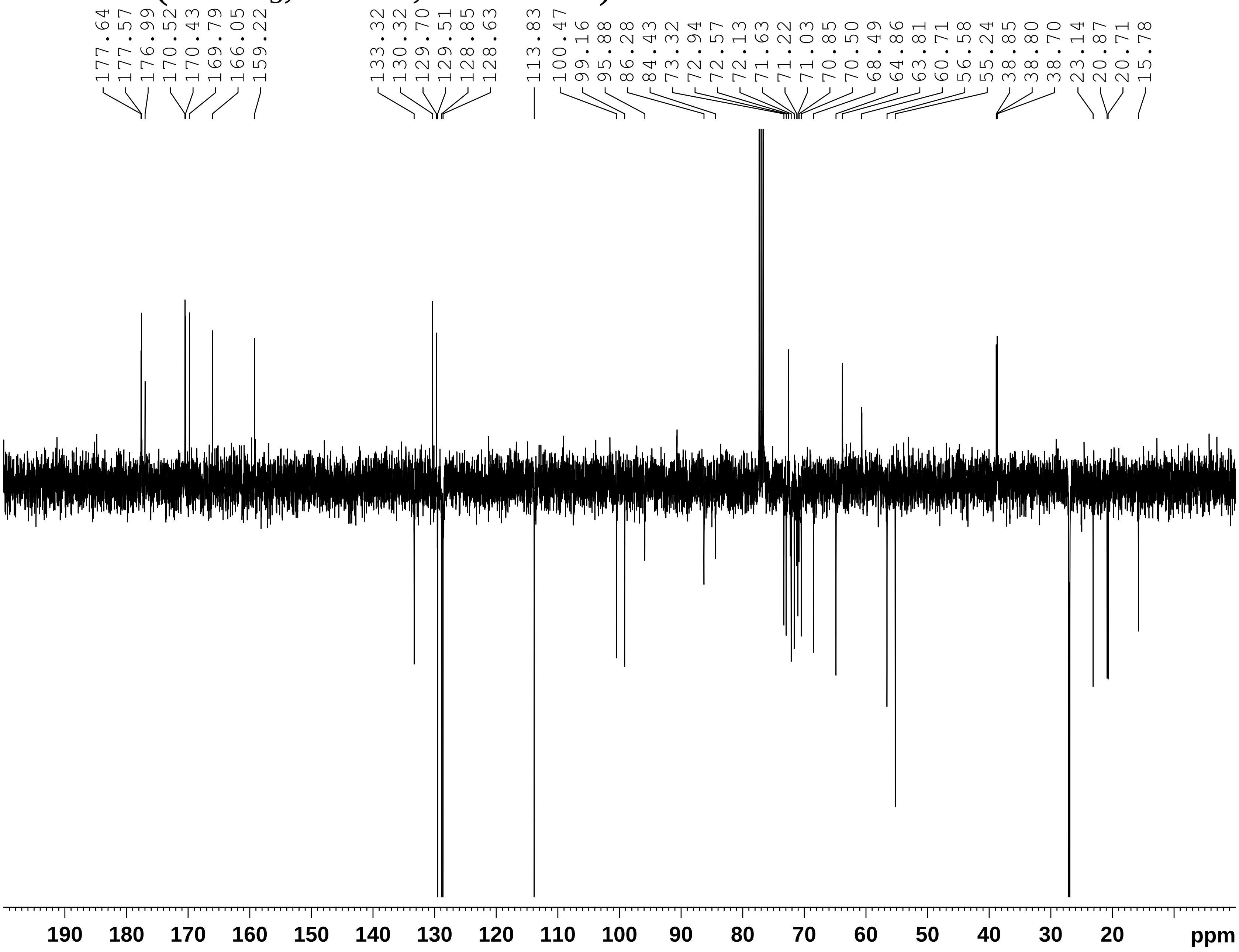

Supplement: File 2 — 1H NMR and 13C NMR for compounds 3–5, 8–11, 14–28. [file Beilstein_J_Org_Chem-08-1134-s002.pdf]
